# 3D Optical Coherence Tomography image processing in BISCAP: characterization of biofilm structure and properties

**DOI:** 10.1093/bioinformatics/btae041

**Published:** 2024-01-23

**Authors:** Diogo A C Narciso, Ana Pereira, Nuno O Dias, Manuel Monteiro, Luis F Melo, Fernando G Martins

**Affiliations:** CERENA—Centro Recursos Naturais e Ambiente, Department of Chemical Engineering, Instituto Superior Técnico, University of Lisbon, 1049-001 Lisbon, Portugal; LEPABE—Laboratory for Process Engineering, Environment, Biotechnology and Energy, Chemical Engineering Department, Faculty of Engineering, University of Porto, Porto 4200-465, Portugal; ALiCE—Associate Laboratory in Chemical Engineering, Chemical Engineering Department, Faculty of Engineering, University of Porto, Porto 4200-465, Portugal; LEPABE—Laboratory for Process Engineering, Environment, Biotechnology and Energy, Chemical Engineering Department, Faculty of Engineering, University of Porto, Porto 4200-465, Portugal; ALiCE—Associate Laboratory in Chemical Engineering, Chemical Engineering Department, Faculty of Engineering, University of Porto, Porto 4200-465, Portugal; LEPABE—Laboratory for Process Engineering, Environment, Biotechnology and Energy, Chemical Engineering Department, Faculty of Engineering, University of Porto, Porto 4200-465, Portugal; ALiCE—Associate Laboratory in Chemical Engineering, Chemical Engineering Department, Faculty of Engineering, University of Porto, Porto 4200-465, Portugal; LEPABE—Laboratory for Process Engineering, Environment, Biotechnology and Energy, Chemical Engineering Department, Faculty of Engineering, University of Porto, Porto 4200-465, Portugal; ALiCE—Associate Laboratory in Chemical Engineering, Chemical Engineering Department, Faculty of Engineering, University of Porto, Porto 4200-465, Portugal; LEPABE—Laboratory for Process Engineering, Environment, Biotechnology and Energy, Chemical Engineering Department, Faculty of Engineering, University of Porto, Porto 4200-465, Portugal; ALiCE—Associate Laboratory in Chemical Engineering, Chemical Engineering Department, Faculty of Engineering, University of Porto, Porto 4200-465, Portugal

## Abstract

**Motivation:**

BISCAP is a state-of-the-art tool for automatically characterizing biofilm images obtained from Optical Coherence Tomography. Limited availability of other software tools is reported in the field. BISCAP’s first version processes 2D images only. Processing 3D images is a problem of greater scientific relevance since it deals with the entire structure of biofilms instead of their 2D slices.

**Results:**

Building on the image-processing principles and algorithms proposed earlier for 2D images, these were adapted to the 3D case, and a more general implementation of BISCAP was developed. The primary goal concerns the extension of the initial methodology to incorporate the depth axis in 3D images; multiple improvements were also made to boost computational performance. The calculation of structural properties and visual outputs was extended to offer new insights into the 3D structure of biofilms. BISCAP was tested using 3D images of biofilms with different morphologies, consistently delivering accurate characterizations of 3D structures in a few minutes using standard laptop machines. Low user dependency is required for image analysis.

**Availability and implementation:**

BISCAP is available from https://github.com/diogonarciso/BISCAP. All images used in the tutorials and the validation examples are available from https://web.fe.up.pt/∼fgm/biscap3d.

## 1 Introduction

Biofilms are communities of microorganisms embedded in a self-produced matrix of Extracellular Polymeric Substances (EPS) commonly found in aqueous environments and usually adhered to surfaces. Biofilms are the most prevailing life forms on earth (Flemming *et al.* 2016) and have a significant economic impact across sectors like industry, medicine, and healthcare systems (Camara *et al.* 2022).

The EPS matrix, one of the most relevant features of biofilms, is a complex 3D porous structure that protects microorganisms from harsh external conditions, regulates several interactions within the consortium, and confers mechanical strength to biofilms (Flemming 2016, Quan *et al.* 2022). As such, understanding biofilm structure, namely biofilm–fluid interactions and their mechanical function, is paramount for most biofilm processes. As Wagner and Horn (2017) highlighted, the mesoscale offers a promising path to study biofilm structures ranging from 100 µm to several mm and complements the information gathered under microscale analysis (10–100 µm).

Several imaging techniques are commonly used for microscale biofilm studies, including Scanning Electron Microscopy (Wolf *et al.* 2002) or Confocal Laser Scanning Microscopy—CLSM (Beyenal *et al.* 2004). More recently, Optical Coherence Tomography (OCT) has become a popular tool for biofilm research at the mesoscale owing to its advantages over other techniques, namely because it is non-invasive, does not require sample staining, and allows excellent penetration depth (Haisch and Niessner 2007, Hou *et al.* 2019).

Measurements from OCT scans are based on the scattering of reflected light, from where normalized greyscale intensities (0 to 255) are assigned to all scanned positions. Biomass/background regions are depicted as predominantly white/black (high/low greyscale values), respectively, thus allowing clear visualization of biofilm structures.

The structural characterization of these systems, such as calculating biofilm thicknesses, is critical in biofilm research. Given the complexity of biofilm structures and the size of 2D/3D images, it is not practical nor objective to undertake this task manually. A range of automatic image-processing techniques has been developed for this purpose. This includes thresholding techniques (Otsu 1979), support interface detection (Dreszer *et al.* 2014), and continuity testing (Heydorn *et al.* 2000).

To the best of the authors’ knowledge, the only known fully automatic and generally available tool for 2D OCT image processing was proposed by Narciso *et al.* (2022), where Biofilm Imaging and Structure Classification Automatic Processor (BISCAP) is presented. BISCAP is a graphical user interface covering all aspects of image processing, including pixel thresholding and identification of biofilm contours. All fundamental principles driving these tasks are carefully framed, on which all processing algorithms are then built and shown to deliver remarkably accurate results.

While this represents a significant methodological step in the characterization of these systems, the complete characterization of 3D OCT biofilm images is a problem of greater significance to the biofilm field since these microbial structures are inherently 3D. In this work, the methodology of Narciso *et al.* (2022) is expanded to the 3D case to account for the additional depth axis in 3D images. As a result of the significant increase in processing complexity, a particular focus is given to developing computationally efficient algorithms to alleviate total processing times while keeping a low user dependency.

The rest of the article is organized as follows. Section 2 builds on the methodology developed for 2D images and discusses the necessary adaptations to address the 3D case. The application of the expanded methodology is illustrated in Section 3, and results are then discussed in Section 4.

## 2 Materials and methods

**Figure 1.**
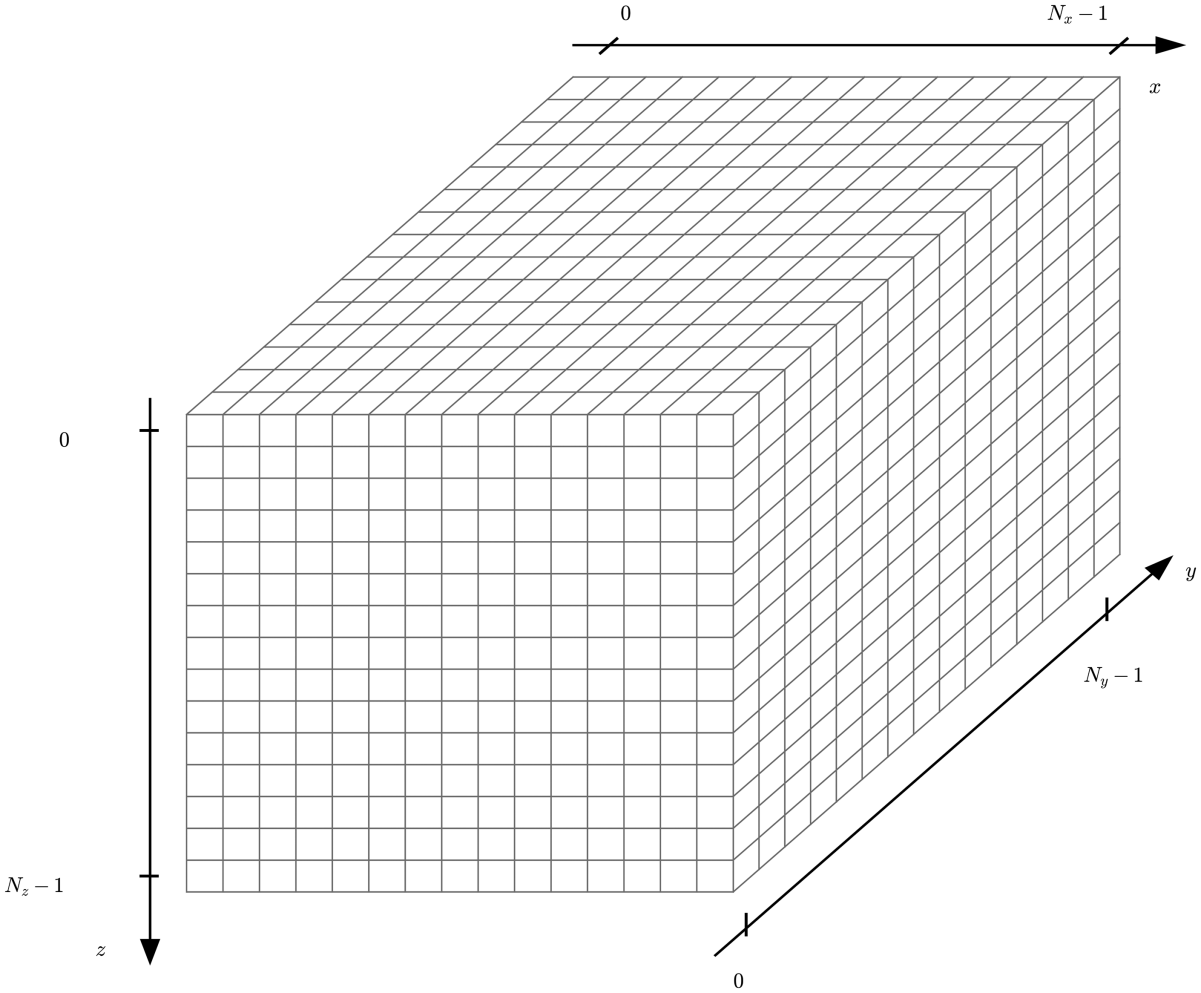
Geometry and axes of 3D OCT biofilm images.

A schematic representation of 3D images obtained from OCT scans is depicted in Fig. 1.

Consistently with the notation in Narciso *et al.* (2022), the vertical and horizontal axes are referred to as z and x, respectively, and include a total of N_z_ and N_x_ unique positions along the corresponding axes. An additional depth axis y of size N_y_ is also defined in 3D images. We refer to the N_y_ × N_z_ × N_x_ individual coordinates in Fig. 1 as ‘voxels’; each is assigned a greyscale intensity from OCT scans (Wagner and Horn 2017).

All image-processing tasks are implemented in Python (Van Rossum and Drake 2009). The first step is converting 3D images in ‘tiff’ formats to an equivalent matrix representation (Beyenal *et al.* 2004) in the Python environment. This is achieved via the OpenCV library (Bradski 2000). 3D arrays are obtained using numpy (Harris *et al.* 2020), and this library is also used to perform all image-processing steps. Matplotlib (Hunter 2007) was used to generate 3D surfaces of top interface voxels. The Tkinter library (Lundh 1999) was used to create/expand BISCAP and now allows the processing of 2D and 3D OCT images.

The same stages of image processing as proposed in Narciso *et al.* (2022) are adapted to the 3D case, namely (i) pre-processing, (ii) automatic processing, and (iii) post-processing. All image-processing principles highlighted in this article also apply to 3D images. A high-level discussion of these principles and how to adapt algorithms from 2D to 3D is presented in sequence. It looks specifically at incorporating the additional depth axis of 3D images into image processing. Supplementary Algorithms S1–S6 deliver the desired functionalities and may be read as Python code in the BISCAP project folder; detailed descriptions are also presented in Supplementary Appendix S1. The Supplementary User Manual provides detailed information on BISCAP from a user perspective. Table 1 summarizes all variables used in this work.

**Table 1.** Variables definition.

Symbol	Description
N_y_, N_z_, N_x_	Number of voxels in depth/vertical/horizontal axes.
y, z, x	Voxel position in depth/vertical/horizontal axes (y = 0, 1, …, N_y_−1, z = 0, 1, …, N_z_−1, x = 0, 1, …, N_x_−1).
i	Greyscale voxel intensity (i = 0, …, 255).
I^raw^, I^pre^	Greyscale voxel intensity matrices in raw/pre-processed images.
vx_len_	Vertical voxel length (µm).
z^bot^, z^top^ (y, x)	Complete set of coordinates at biofilm bottom/top interfaces.
i^void^	The representative intensity of the top region.
i^tresh^	Threshold intensity for voxel binarization.
S^bin^, S^str^, S^int^	Voxel classification matrices (Steps 3, 4, and 5).
p, m	Percentile/multiplication parameters for threshold intensity calculation (0 ≤ p ≤ 100, m > 1).
L_F_(y, x)	Biofilm thickness at given (y, x) (µm).
$\bar{L_{F}}$	Average biofilm thickness (µm).
R_α_	Biofilm roughness (µm).
R_α_^*^	Biofilm roughness coefficient.
C_p_	Compaction parameter.
Φ	Porosity.
y^bands^, x^bands^	The number of bands in depth/horizontal axes (parallel processing).

### 2.1 Pre-processing (Step 1)

Biofilm images comprise three distinct regions: ‘biofilm’, ‘bottom’ (support), and ‘top’ (bulk liquid phase). A graphical illustration of these regions is presented in Supplementary Appendix S1. The biofilm region includes all biofilm voxels and all background voxels fully enveloped by the biofilm structure. This region is typically included in a narrow horizontal band of voxels, also comprising small portions of the bottom and top regions. All voxels above and below this band are not significant to the characterization of biofilm structures and may be ignored with no consequence to calculating the structural parameters. In Step 1, images are trimmed to include only this central band of voxels. This is consistent with the approach used for 2D images where a lower and upper bound on z are selected ‘manually’. Since 3D images comprise, in fact, a total of N_y_ 2D images/slices, care must be taken to ensure that these bounds do not exclude any biofilm voxels. Supplementary Algorithm S1 trims the original biofilm image (I^raw^) to deliver the corresponding pre-processed image (I^pre^).

### 2.2 Automatic processing

#### 2.2.1 Step 2: bottom interface

A strategy comprising three algorithms was proposed by Narciso *et al.* (2022) to detect all pixels at the boundary between the biofilm and bottom regions and referred to as the ‘bottom interface’. It targets the identification of the ‘brightest’ pixels along the vertical axis for all x in the horizontal axis, from where an initial estimate and a series of smoothing steps generally deliver the position of this interface very accurately. Since 3D images comprise N_y_ slices along the depth axis, to identify all bottom interface voxels in the 3D case, it suffices to execute this strategy to all their slices at y = 0, 1, …, N_y_−1 via Supplementary Algorithm S2.

**Figure 2.**
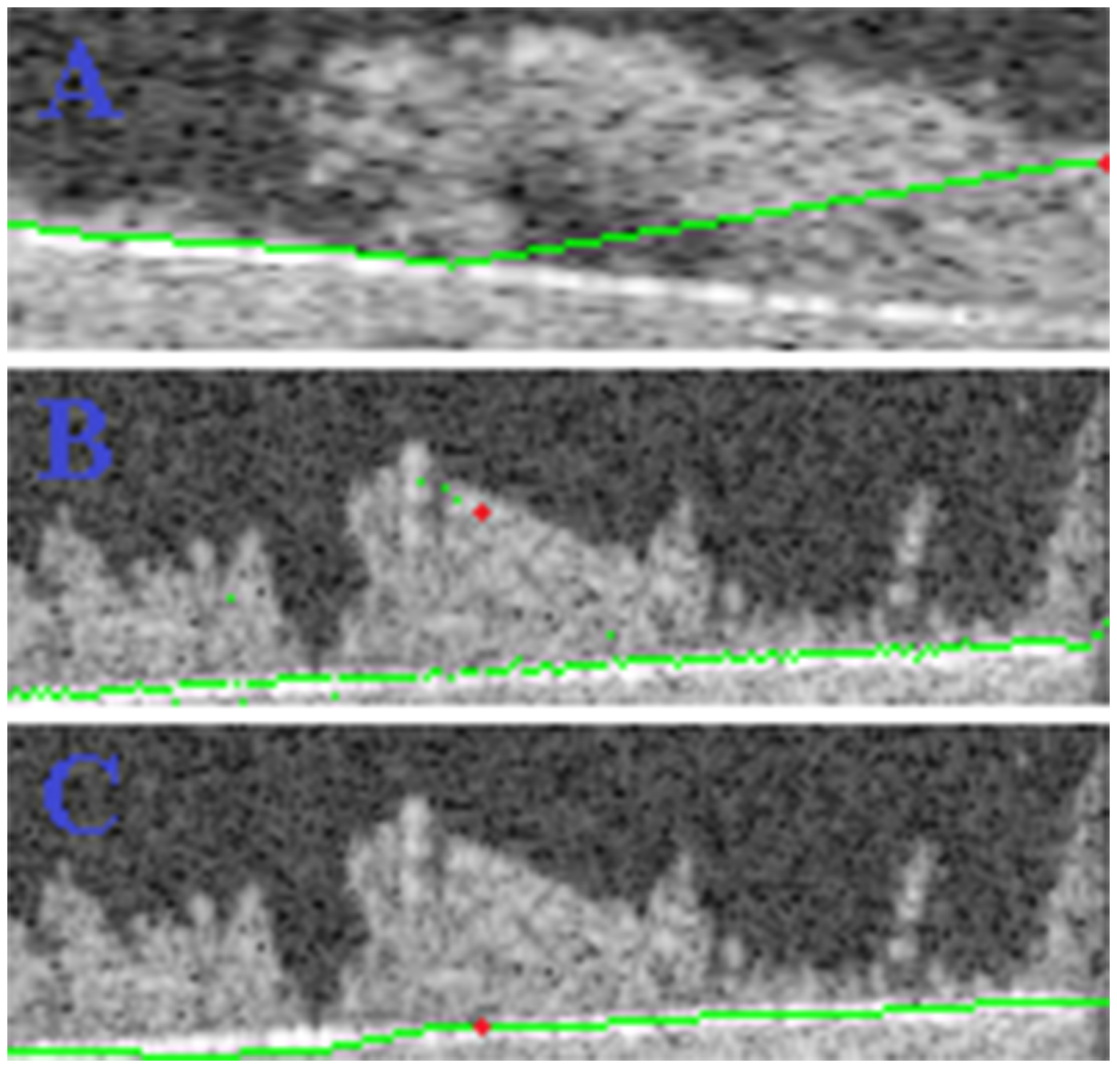
Bottom interface calculation in 3D images: smoothing in the depth axis. (**A**) The standard 2D strategy for bottom interface detection is applied to slice y = 3, where a deviation is observed at x > 40. (**B**) The interface is visualized at slice x = 179; voxel (3, 25, 179) is highlighted in both cases as a large dot. In this context, it is more apparent that this and other voxels do not accurately represent the bottom interface. (**C**) A final smoothing step is applied along the depth axis, where this and all remaining spurious points are flattened.

To improve the initial estimate, a final ‘smoothing’ step is implemented in 3D images: examining the bottom interface on constant x slices, this smoothing step is executed along the depth axis as schematically illustrated in Fig. 2.

#### 2.2.2 Step 3: voxel binarization

This step requires minimal adaptation from the 2D case. Firstly, a representative intensity of the void region (i^void^) is computed via the p-percentile of all intensities in a top band of voxels above the biofilm structure. In the 3D case, this includes all voxels in the depth axis of the defined band, additionally to the vertical and horizontal axes used in 2D, to ensure a representative estimate of i^void^ is obtained. The intensity threshold is calculated as i^thresh^ = i^void^ × m, where m is a pre-defined parameter (Supplementary Algorithm S3). Alternatively, i^void^ may be manually specified. For all voxels, if their intensities are above/below the calculated threshold, voxels are classified accordingly as biomass/background (Supplementary Algorithm S4).

#### 2.2.3 Step 4: biofilm structure

Consistently with Heydorn *et al.* (2000), biofilm voxels include the subset of biomass voxels, defining a continuous structure of voxels attached to the bottom interface. Note that this definition excludes any ‘floating’ biomass voxels where no continuous connection with the bottom interface can be detected. The initial layer of biofilm voxels is obtained by checking which voxels immediately above this interface are biomass.

Starting from this initial layer, all remaining biofilm voxels are then identified by checking, sequentially, the vicinity of all biofilm voxels in any given iteration to identify a new set of ‘unexplored’ neighbour biomass/biofilm voxels to ‘expand’ the biofilm region in the next iteration (if any). Biofilm continuity testing is implemented in Supplementary Algorithm S5 and proceeds over a finite number of iterations until no additional biofilm voxels are detected. In 3D images, a significant update to the calculation of all relevant neighbour voxels is necessary to accommodate for continuity testing over the depth axis: in this case, a total of 26 neighbour voxels exist in the vicinity of any voxel (y, z, and x) in the interior region of 3D images (excluding their outer edges). The geometry and calculation of neighbour voxels are significantly more complex in 3D and presented in greater detail in Supplementary Appendix S1.

#### 2.2.4 Step 5: top interface

All voxels in the top region neighbour to at least one biofilm voxel define the ‘top interface’. This set of voxels effectively establishes the interface between the biofilm and top regions, and their calculation is fundamental to the characterization of the internal properties of biofilms (e.g. porosity). These voxels are calculated based on an equivalent continuity test as presented in Step 4, except that in this case, continuity testing begins from an initial layer of background voxels above the biofilm structure and allows the expansion of background voxels in the top region only (Supplementary Algorithm S6). Two continuity models enabling the calculation of the top interface are now proposed and available for this step; their principles are enunciated below:

**Deep continuity:** top region is allowed to extend from all non-biofilm voxels, ‘including’ those at the top interface.

**Shallow continuity:** top region is allowed to extend from all non-biofilm voxels, ‘excluding’ those at the top interface.

**Figure 3.**
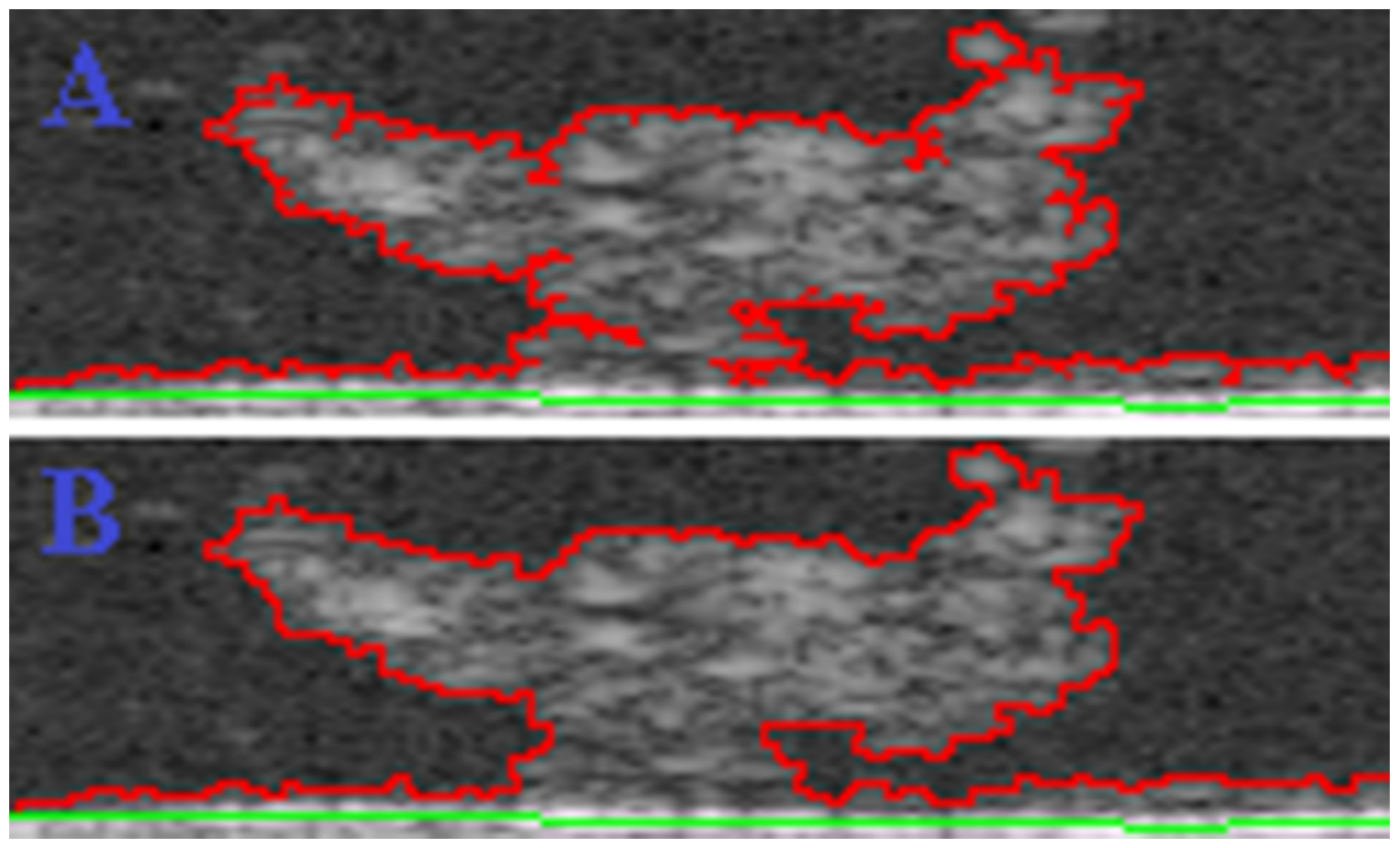
Deep (**A**) versus shallow (**B**) continuity in the top region.

The application of these two models is illustrated in Fig. 3 via a 2D example. The first model allows the top region to extend ‘deep’ into the biofilm structure in a network of narrow channels [as proposed in Narciso *et al.* (2022)]. The second model is more restrictive and preserves the ‘shallow’ external contours of the biofilm region; only larger channels of the top region are admissible in this case. These models enable two distinct and complementary approaches towards calculating biofilm porosity.

In the final processing step, all voxels in the biofilm and top regions are trivially segregated: the biofilm region includes all biofilm voxels and all unexplored (background) voxels after the continuity test is concluded.

#### 2.2.5 A closer look at continuity algorithms

Supplementary Algorithms S5 and S6 are the most computationally intensive in the proposed architecture, given the large number of operations required per voxel. This section outlines the critical algorithmic developments enabling their efficient execution.

In the first version of BISCAP, identifying all neighbour pixels in any given iteration was based on ‘stacking’ all relevant coordinates sequentially to an auxiliary vector. This potentially leads to repeating coordinates and thus requires removing duplicates at the end of all iterations. This approach is feasible in 2D images and quickly becomes prohibitive as the size of 3D images grows larger.

To deal with this limitation, more efficient versions of all continuity algorithms were developed in accordance with the principles highlighted earlier. A new and equivalent strategy to test voxel continuity is summarized in three steps:

1)  **Count:** for all voxels to be processed in the ‘current’ iteration, count the neighbour—‘unexplored’*—*voxels to be processed in the ‘next’ iteration (n).

2)  **Initialize next voxels:** define a matrix of zeros of size (n, 3).

3)  **Populate next voxels:** repeat the search in 1) and assign each of the identified n coordinates of neighbour voxels to this matrix.

To achieve this, voxels are given a temporary status in 1) to avoid double counting. An index iterator is used in 3) to ensure all voxels are assigned to each of the n rows of the matrix of coordinates. This equivalent strategy avoids vector stacking and offers a much faster route to classification, particularly in large images.

**Figure 4.**
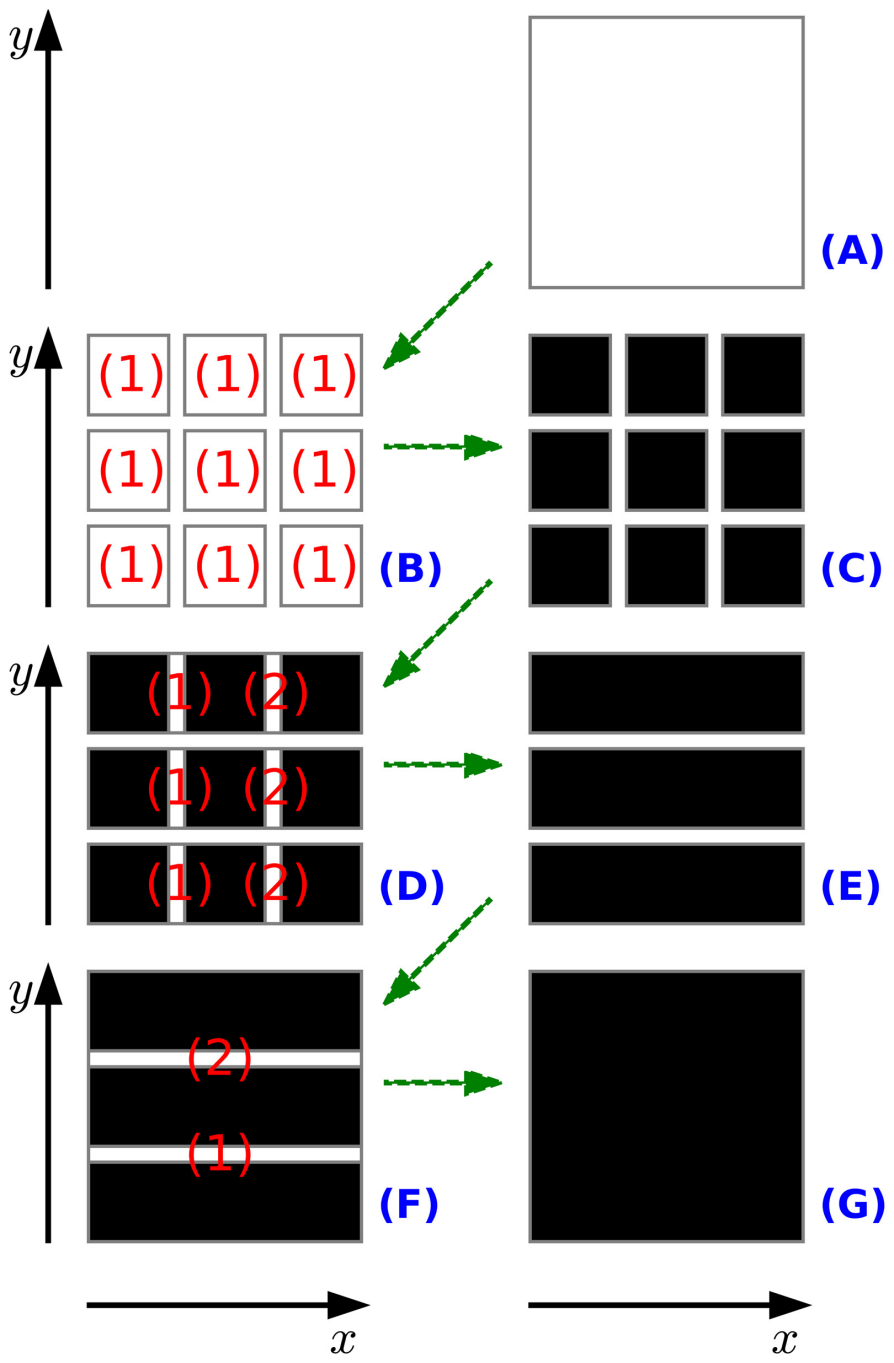
Parallel processing architecture. (**A**) Initialized classification matrix (full); (**B**) matrix is partitioned in y^bands^*x^bands^ small chunks (illustrated with y^bands^=x^bands^=3). A process is created per chunk to launch continuity testing simultaneously; (**C**) partial processing of small chunks completed; (**D**) classification matrix is partly restored from the small chunks to create a total of y^bands^ large chunks. One process is created per large chunk, and continuity is tested sequentially between their unprocessed edges; (**E**) partial processing of large chunks completed; (**F**) full classification matrix is restored from large chunks. A single process launches continuity testing sequentially between their unprocessed edges; (**G**) classification matrix fully processed.

Parallel processing was also used in Steps 4 and 5. This allows all classification tasks to be ‘distributed’ over several processes and thus compresses them in shorter processing times. The key to achieving this is partitioning classification matrices into smaller ‘chunks’, which are then processed individually and in parallel in as many processes. The proposed parallel processing strategy is schematically depicted in Fig. 4.

The proposed design is such that all steps allow the expansion of the biofilm/top regions in a very localized fashion per each step instead of launching a final continuity across all edges between chunks. A computational cost is associated with the tasks highlighted in Fig. 4, namely launching all parallel processes. Except for small images, where these overheads may be unfavourable, parallel processing generally contributes to faster processing times. There are several nuances in applying this architecture to Steps 4 and 5, which are presented in greater detail in Supplementary Appendix S1.

#### 2.2.6 Image-processing summary

**Figure 5.**
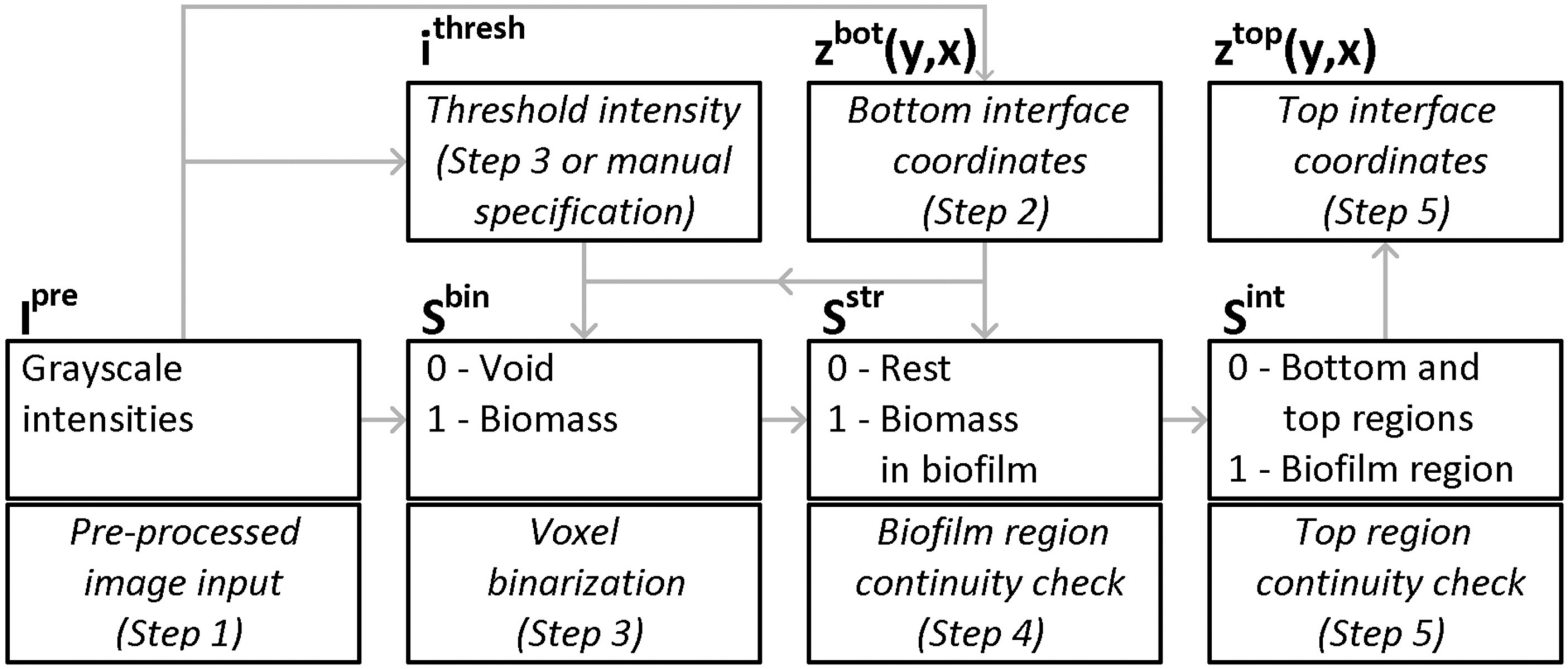
Summary of image-processing steps [adapted from Narciso *et al.* (2022)].

All image-processing steps are summarized in Fig. 5.

All classification information from 3D image processing is saved in Python as follows: (i) I^pre^ is the pre-processed 3D image/array of size (N_y_, N_z_, N_x_), (ii) i^thresh^ is the threshold intensity, (iii) z^bot^ and z^top^ are 2D arrays, listing the complete set of (y, z, x) coordinates of I^pre^ associated with the bottom and top interfaces, respectively, and (iv) S^bin^, S^str^, and S^int^ are 3D arrays of the same shape as I^pre^, where the classification information from Steps 3, 4, and 5, respectively, is saved.

#### 2.2.7 Image-processing outputs

Using these arrays, it is possible to calculate several biofilm structural parameters. Consistently with Narciso *et al.* (2022) and Romeu *et al.* (2022), biofilm thickness (*L*_F_) is calculated as follows:

(1) $$L_{F}(y,x)=(z^{\mathrm{bot}}\left(y,x\right)-\min\left(z^{\mathrm{top}}\left(y,x\right)-1\right){vx}_{\mathrm{len}},y=0,\;1,\;\ldots ,\;N_{y}-1,\;x=0,\;1,\;\ldots ,\;N_{x}-1.$$

For all pairs (y, x), the total number of voxels between the highest position in the top interface and the bottom interface is calculated. Then, taking vx_len_, the complete series of biofilm thickness is calculated in µm. The average value of L_F_ is denoted as $\bar{L_{F}}$. Next, the definitions of biofilm roughness (R_α_) and the roughness coefficient (R_α_*) are updated to 3D:

(2) $$R_{\alpha }=\frac{1}{{N_{y}N}_{x}}\sum_{i=0}^{N_{y}-1}\sum_{j=0}^{N_{x}-1}\left|L_{F}\left(i,j\right)-\bar{L_{F}}\right|.\;$$

(3) $$R_{\alpha }^{*}=\frac{1}{{N_{y}N}_{x}}\sum_{i=0}^{N_{y}-1}\sum_{j=0}^{N_{x}-1}\frac{\left|L_{F}\left(i,j\right)-\bar{L_{F}}\right|}{\bar{L_{F}}}.\;$$

The compaction parameter (0 < C_p_≤ 1) defined in Narciso *et al.* (2022) provides a measurement of how strongly the biofilm structure is compacted towards the bottom interface; its 3D definition is as follows:

(4) $$C_{P}=\;\frac{\sum_{i=0}^{N_{y}-1}\sum_{j=0}^{N_{z}-1}\sum_{k=0}^{N_{x}-1}S^{\mathrm{str}}(i,j,k)}{\sum_{i=0}^{N_{y}-1}\sum_{k=0}^{N_{x}-1}L_{F}(i,k)/{vx}_{\mathrm{len}}}.\;$$

Consistently with Wagner and Horn (2017), porosity (Φ) is conceptually defined as the fraction of background voxels in the biofilm region. Using S^srt^ and S^int^ to compute their total counts, porosity is calculated accordingly:

(5) $$\Phi =\;1-\frac{\sum_{i=0}^{N_{y}-1}\sum_{j=0}^{N_{z}-1}\sum_{k=0}^{N_{x}-1}S^{\mathrm{str}}\left(i,j,k\right)}{\sum_{i=0}^{N_{y}-1}\sum_{j=0}^{N_{z}-1}\sum_{k=0}^{N_{x}-1}S^{\mathrm{int}}\left(i,j,k\right)}.\;$$

Upon completion of automatic processing, a set of images is created to facilitate the inspection of the biofilm structure. The complete set of delivered outputs is listed in Table 2.

**Table 2.** Image-processing outputs.

Output	Description
Biomass	Biomass/non-biomass voxels in white/black.
Biofilm	Biofilm/non-biofilm voxels in white/black.
Bounds	Bottom and top interface voxels are given a high contrast colour (e.g. red)—highlighting biofilm contours.
Structure	All voxels in the top and bottom regions are given a neutral colour (e.g. black)—emphasizing the biofilm region.
Thickness	Thickness profile (shown as a heatmap).
Topography	3D topography of voxels at the top interface.

We remark that the outputs from Steps 3 and 4 are now also available to users (biomass and biofilm). The thickness profile is no longer a 1D series but a greyscale heatmap (highest/lowest thicknesses shown in white/black). Representation of the top interface 3D topography was developed to offer a complementary view of biofilm shape.

### 2.3 Post-processing (Step 6)

This is an optional final step, where users may manually select any ‘region of interest’ along the horizontal and depth axes to obtain the corresponding structural properties of biofilm. The first four 3D image outputs in Table 2 may now be visualized in BISCAP as 2D slices of any of the three axes.

## 3 Results

All results presented in this section aim to: (i) illustrate BISCAP’s image-processing features and (ii) demonstrate its accuracy in 3D calculations. Image processing was successfully applied on multiple 3D OCT images, and for brevity, the processing of a single image is presented here. Since most image outputs are inherently 3D, it is impossible to fully illustrate them in this manuscript. Readers are encouraged to explore the full potential of BISCAP for image analysis in biofilm research.

A set of tests were performed to validate that the implementation of Supplementary Algorithms S1–S6 in Python performs as expected. To this end, three 3D images were artificially designed, for which it is possible to perform manual calculations without the need of the continuity tests employed in Supplementary Algorithms S5 and S6. All images are defined with flat surfaces, which offer a convenient way to compute the full count of voxels in matrices S^str^ and S^int^. The full details of these calculations are presented in Supplementary Appendix S1, which demonstrates that the results from automatic processing in BISCAP match those from the independent manual calculations, thus providing strong evidence that all algorithms were correctly implemented in Python.

Before image acquisition and processing, biofilm was formed from a *Pseudomonas fluorescens* ATCC 13525 inoculum, supplied with nutritional medium, and grown in a Center for Disease Control biofilm reactor. The detailed experimental procedure and materials are described in Silva *et al.* (2023). Each coupon was placed inside a sterile 12-well microtiter plate (VWR International, Portugal). Each well was filled with 3 ml of a sterile saline solution (8.5 g/l NaCl, VWR International, Belgium) and imaged afterwards.

Biofilm images were obtained through spectral-domain OCT using a Thorlabs Ganymede (Thorlabs GmbH, Germany) instrument with a central wavelength of 930 nm. All volumes are ∼2.49 × 2.13 × 1.52 mm (y × z × x) and imaged using 509 × 1024 × 730 voxels in the corresponding axes (voxel sizes of 4.89 × 2.08 × 4.85 µm). The refractive index was set to 1.40 to be similar to the refractive index of water (1.33) since biofilms are mainly composed of water.

Image processing is illustrated sequentially with a biofilm sample grown for 8 days, where the reactor’s rotational velocity was set to 225 RPM. The raw image was trimmed in the vertical direction using the guidelines in Section 2.1, where only 317 of the original 1024 positions were kept. A slice of I^pre^ at constant y is shown below.

All default settings for automatic processing were used, namely y^bands^ = x^bands^ = 5. Processing via the shallow and deep modes was executed separately. The total processing time in both modes (including saving all outputs) was ∼9 min on a standard laptop machine.

**Figure 6.**
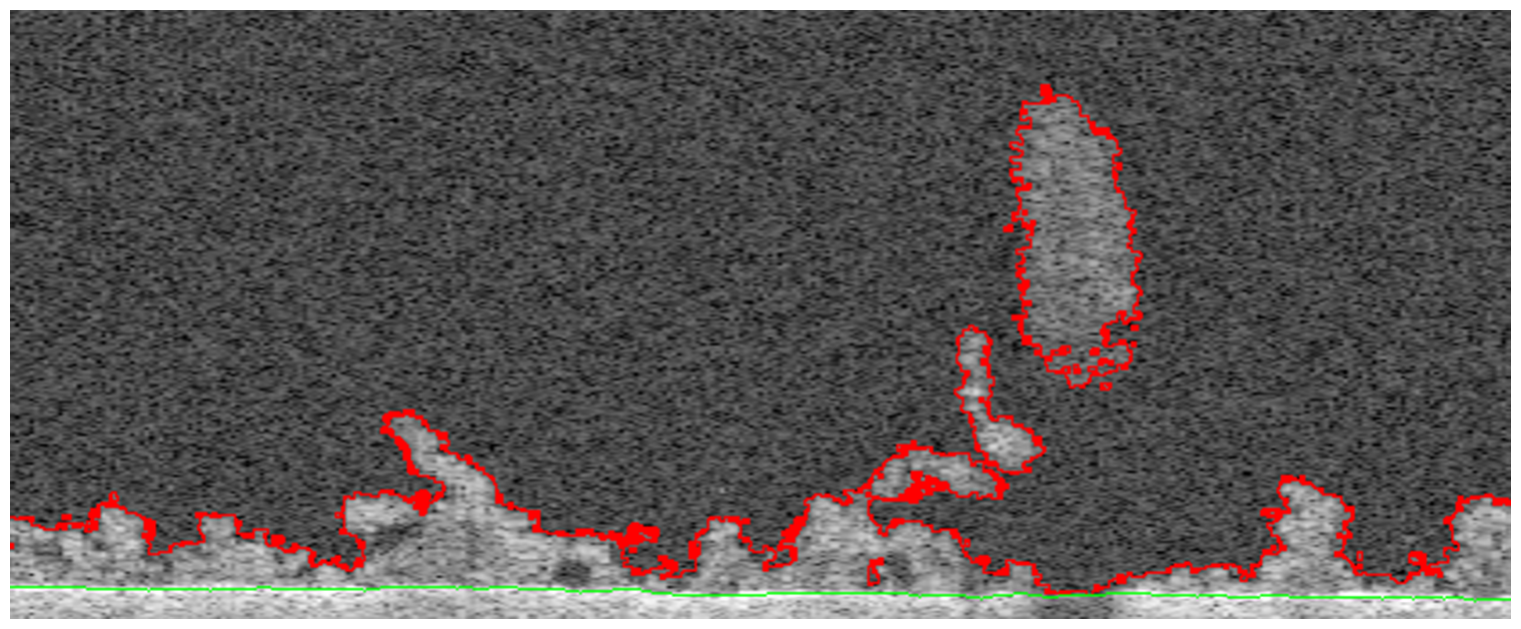
Biofilm contours: bottom/top interface voxels in green/red (shallow mode).

**Figure 7.**
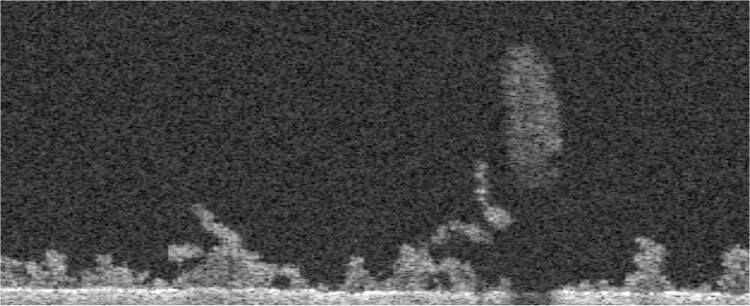
Pre-processed image at a constant y slice.

In Fig. 6, the bounds of biofilm are shown at the same y slice presented earlier. Interestingly, in the context of 2D image processing, the large piece of floating biomass shown in Fig. 7 is not connected to the bottom interface and would be classified as ‘non-biofilm’. However, 3D processing shows that this piece is connected to the bottom interface via neighbour y slices, reinforcing the significance of these developments.

**Figure 8.**
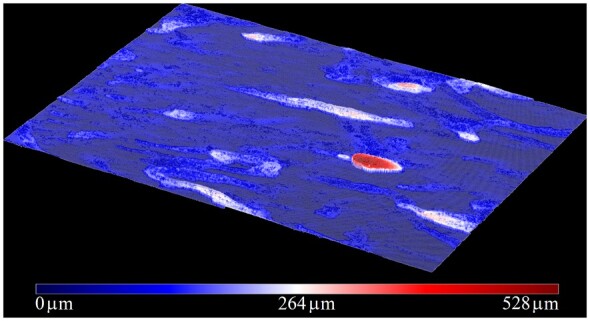
3D biofilm topography (via the set of top interface voxels).

Figure 8 exhibits the topography of the complete set of voxels at the top interface. This provides complementary insight into biofilm structure, where those regions with lower/higher thicknesses are shown in blue/red. For instance, the region with the highest thicknesses (∼520 µm) is shown as an oval (red) structure in Fig. 8. The presence of this structure is also confirmed in Fig. 6, as the portion of biofilm apparently disconnected from the remaining biofilm voxels with the largest thicknesses.

**Figure 9.**
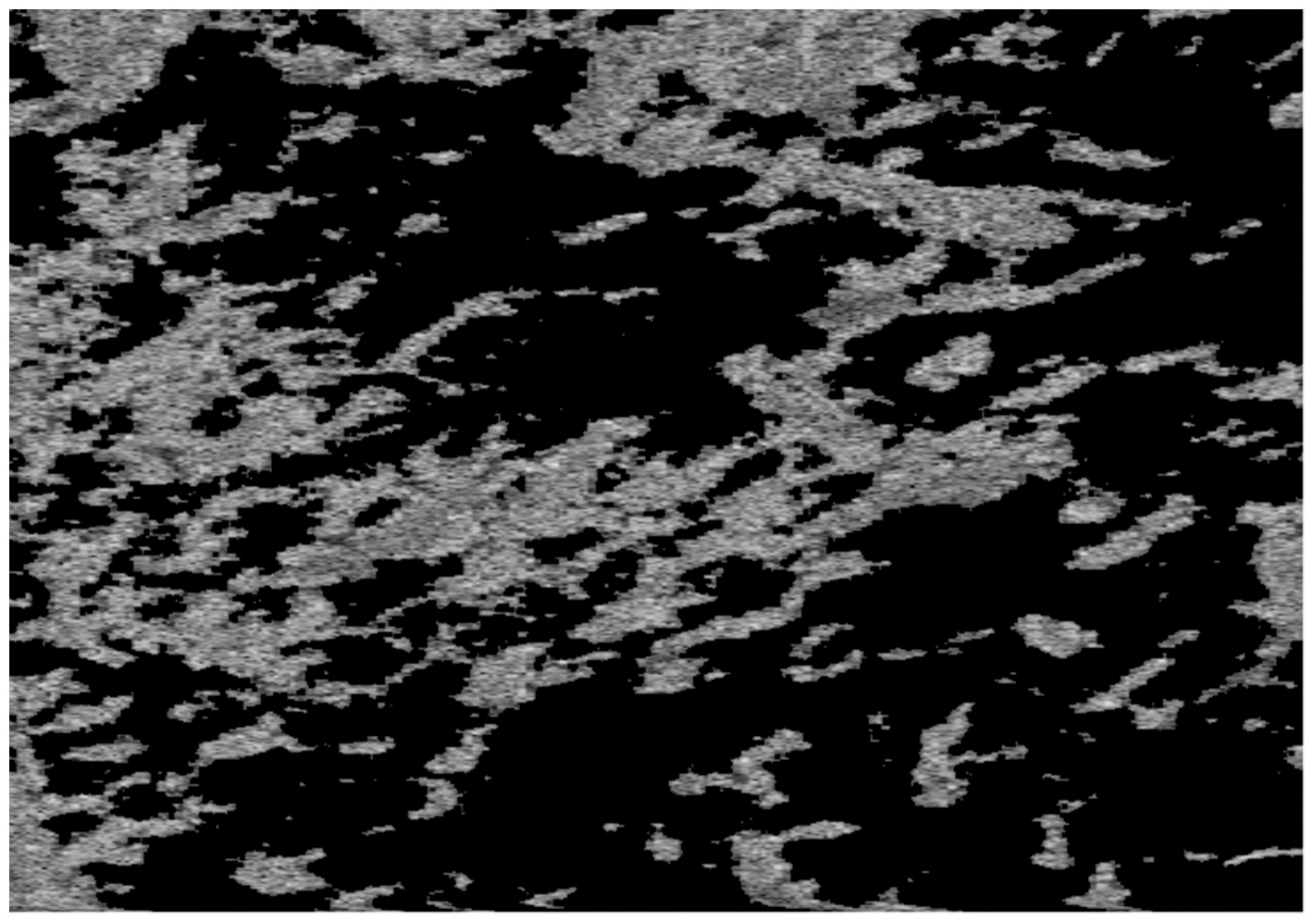
Biofilm structure displayed at a constant z slice (shallow mode).

Figure 9 displays a reconstruction of the biofilm region in a constant z slice. Provided pre-processing limits are correctly set, this view indicates no biofilm voxels are detected around z = 0, and density typically increases towards the bottom interface. The bottom of the biofilm in Fig. 9 is not homogeneously adhered to the surface, with significant areas where no biomass is observed (black areas). These observations are consistent with the ones presented in Lewandowski (2000) while reconstructing CLSM slides from multi-species biofilms. Visualization of constant x slices is also possible, exhibiting a geometry more consistent with constant y slices. These options offer a new and rich perspective on the biofilm structure with no additional image processing required.

All structural parameters were calculated from Equations (1) to (5). Note that the deep and shallow modes yield the same results for all parameters except porosity: (i) $\bar{L_{F}}$ = 71.1 µm, (ii) R_α_=44.8 µm, (iii) R_α_^*^=0.630, and (iv) C_p_=0.703. Porosity (Φ), as calculated from the deep and shallow modes, is 0.015 and 0.122, respectively. The higher porosity in the shallow mode is expected since continuity in this mode excludes many background voxels from the top region, which contributes instead to the set of background voxels in the biofilm region.

## 4 Discussion

The proposed image-processing strategy was successfully applied to 3D images, including complex biofilm shapes and inclined substratum surfaces. Detection of the bottom interface remains the element of image processing most prone to calculation errors, given that this is based on assessing a noisy greyscale series. Introducing a final smoothing step in Supplementary Algorithm S2 along the depth axis in 3D images has proved very effective in dealing with any remaining errors observed during this stage. The subjectivity in selecting a threshold intensity for voxel binarization is also largely covered in this work. Users must fine-tune parameters p and m for best results depending on image acquisition parameters.

The remaining classification tasks are based on more explicit criteria, from where very accurate calculations of contours and structural properties are consistently obtained; the efficient computation of all classification tasks, rather than accuracy, was the main focus during algorithm development in 3D processing. A significant effort was made to speed up all classification tasks, including via the use of parallel processing. Considerable gains are achieved in 2D processing (<5 s); 3D processing is completed in 5–10 min.

Two models have been developed for calculating the top interface (deep and shallow), each offering a different view of biofilm structure. At this stage, it is unclear which of these models captures biofilm structure more accurately. We expect their relevance to become clearer as the two models are used to characterize these structures in future work. These models are particularly relevant for porosity calculations, where the shallow mode delivers substantially higher porosities than the deep mode. As highlighted in Lewandowski (2000), determining and interpreting biofilms’ porosity is a complex problem associated with technical limitations and conceptual approaches. Although a detailed discussion on porosity is out of the scope of this article, it should be remarked that very different porosity ranges can be found across the literature. For example, Lewandowski (2000) reported a porosity increase from 0.67 to 1.00 between the biofilm’s bottom and top while analysing biofilm CLSM images. Zhang and Bishop (1994) found porosity values around 0.8–0.9, using a laboratorial biofilm slicing technique for such determination. Significantly lower biofilm porosity values (∼0.1 in shallow mode) were obtained from the 3D OCT images using BISCAP. Those values, although very low, are closer to the porosities reported in Romeu *et al.* (2022) (also from 3D OCT image processing), ranging between 0.2 and 0.4. Those differences might be due to the biofilm structure or the conceptual interpretation of porosity calculations. As the literature on porosity calculation assumptions is scarce, additional research and development are required on this subject. Apart from specific laboratorial experiments, a more detailed analysis of thresholding should be accomplished, which rather than classifying voxels as biomass/non-biomass, provides a more quantitative assessment of how much dry biomass is included in voxels.

The remaining structural parameters (thickness, roughness, roughness coefficient, and compaction parameter) determined from the 3D processing are consistent with the dataset from ten 2D OCT images obtained from the same biofilm coupons.

To conclude, this work successfully expanded on all the 2D image-processing principles presented in Narciso *et al.* (2022) to enable the fast and accurate classification of 3D OCT biofilm images. BISCAP was fully updated to accommodate 2D and 3D image processing, and where an extended set of options are now made available to analyse and characterize 3D biofilm structure images in detail.

## Supplementary Material

btae041_Supplementary_Data

## Data Availability

BISCAP is available from https://github.com/diogonarciso/BISCAP. All images used in the tutorials and the validation examples are available from https://web.fe.up.pt/∼fgm/biscap3d.
