# Supplementary material for: 3D Optical Coherence Tomography image processing in BISCAP: characterization of biofilm structure and properties
Source: Bioinformatics. 2024 Jan 23;40(2):btae041. doi: 10.1093/bioinformatics/btae041 (PMC10868339; doi:10.1093/bioinformatics/btae041)
Supplement: btae041_Supplementary_Data [file btae041_supplementary_data.zip › appendix1.pdf]

Appendix 1

Table of Contents

A1. Notes on image processing 2

A2. Regions of interest in 3D OCT images 5

A3. Detailed Algorithm Descriptions 6

A3.1 Algorithm 1: Window of interest (pre-processing) 7

A3.2 Algorithm 2: Bottom interface 8

A3.3 Algorithm 3: Threshold intensity 9

A3.4 Algorithm 4: Voxel binarization 9

A3.5 Algorithm 5: Biofilm structure 10

A3.6 Algorithm 6: Top interface 12

A4. Algorithms implementation validation 17

A4.1 Cube 18

A4.2 Polyhedron 20

A4.3 Mushroom 22

A4.4 Automatic processing and validation 25

A5. Default algorithms’ parameters 26

A1. Notes on image processing

In accordance with the standard convention adopted in the field, y, z, and x denote the depth, vertical and horizontal axes in 3D OCT images, respectively. Pre-processed images include a total of $N_y * N_z * N_x$ voxels, where N_y, N_z and N_x stand for the number of voxels in the depth/vertical/horizontal directions, respectively, and where each voxel is represented by a unique integer grayscale colour intensity ($i(y, z, x)$), ranging between 0 (black) and 255 (white):

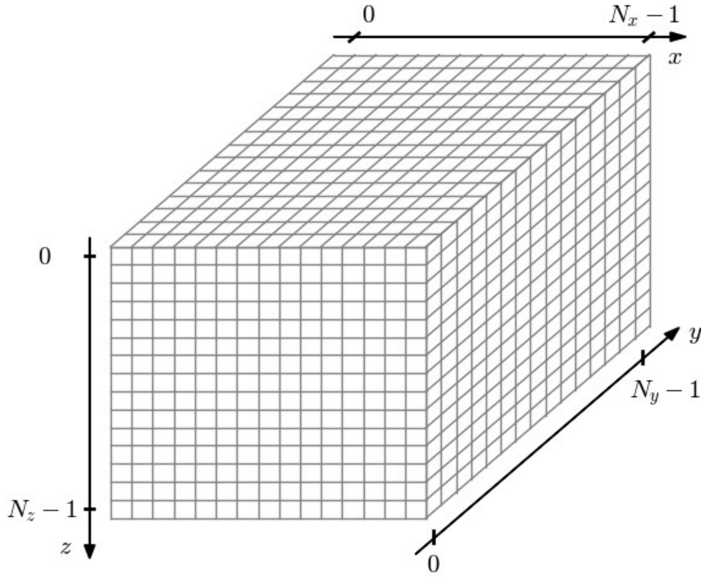

Figure A1: Geometry and axes of 3D OCT biofilm images.

According to Beyenal et al. 2004, two distinct representations are possible for such image files: (i) as grayscale images on a screen, or (ii) digitally, as matrices of size (N_y, N_z, N_x), including all corresponding voxel intensities $i(y, z, x)$, and denoted in this work as I^{pre}. Allowable inputs for 3D image processing in BISCAP are grayscale *tiff* files, consistent with the format above. If the original OCT image files include all RBG colour codes, it suffices to read a single colour channel to obtain I^{pre} as grayscale images.

The calculation of neighbour voxels is a fundamental requirement in Algorithms 5 and 6 (Section A3). This task is more complex in the 3D case since several geometry scenarios must be considered to calculate neighbours in all voxel positions precisely. The 3D voxel geometry is schematically depicted in Figure A2.

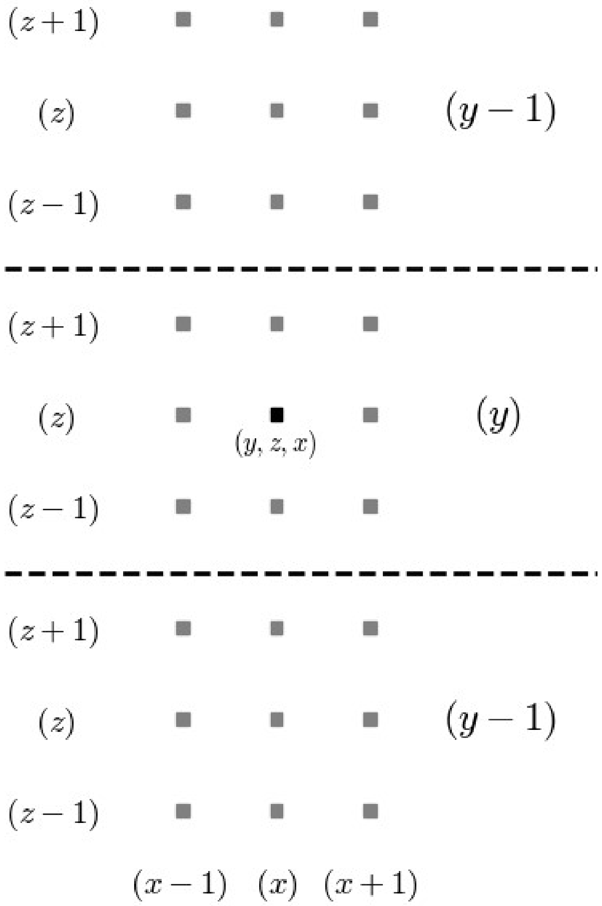

Figure A2: Given a voxel in the *interior region* of a 3D array at (y, z, x), a total of <u>26 neighbour voxels</u> are identified by selectively incrementing all their coordinates. Note that the 2D case corresponds to the middle slice (y), where a total of 8 neighbour pixels/voxels are found. An additional set of 9+9 neighbour voxels from the slices at $(y - 1)$ and $(y + 1)$ must also be considered for a rigorous 3D processing.

In 3D images, continuity testing must accommodate all neighbour voxels identified in Figure A2. In fact, for voxels in different positions of 3D arrays, a distinct set of neighbour voxels are identified, depending on their positions along y and x. In Figure A3, a general

3D image array is partitioned in 9 distinct regions along the horizontal and depth axes, and the matching relevant neighbours presented accordingly.

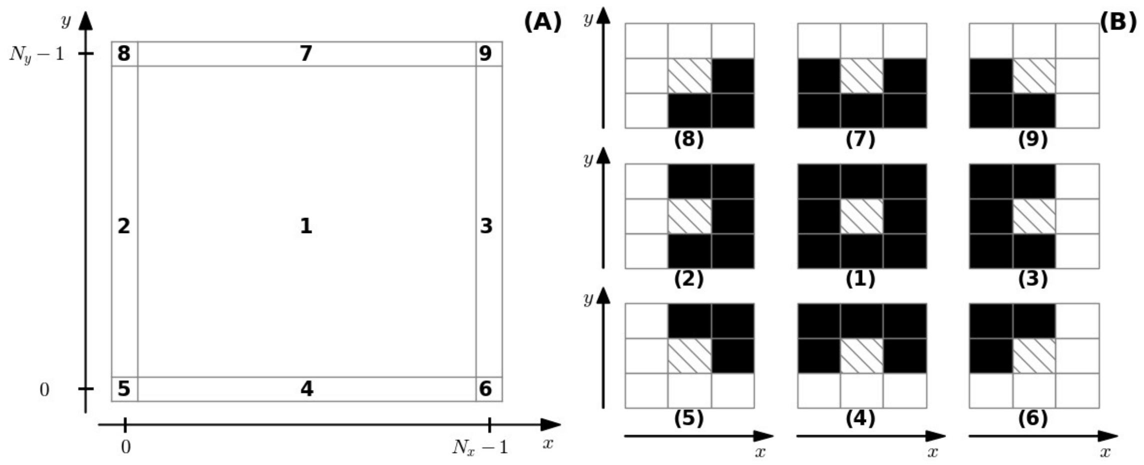

Figure A3: (A) Partitions (1 to 9) of a 3D array along the horizontal and depth axes. (B) For any voxel in one of these partitions, the corresponding neighbour voxels are identified accordingly (1 to 9). In all schematics, the central block represents a given voxel (y, z, x). The dotted lines indicate that the voxels immediately above $(y, z - 1, x)$ and below $(y, z + 1, x)$ are in the vicinity of the given voxel (but excluding the voxel of interest from its own vicinity). Black blocks indicate their neighbour positions along the horizontal and depth axes, including the matching three voxels at $z - 1$, z and $z + 1$. For instance, and consistently with Figure A2, any given voxel in the interior region $(0 < y < N_y - 1, 0 < x < N_x - 1)$, is neighbour to 26 voxels (1); on the other hand, in the bottom left corner $(y = 0, x = 0)$, only those neighbours where $y, x > 0$ are permitted, and thus including only a total of 11 neighbour voxels in this case (5).

All scenarios were coded in Python via a set of increments, each applicable to a specific set of (y, x) positions. These are then used in continuity calculations in Algorithms 5 and 6.

A2. Regions of interest in 3D OCT images

From visual inspection, voxels in OCT images may be qualitatively assigned to 3 distinct regions, as depicted in Figure A4. Biofilm is typically included in a small band of voxels along the vertical direction. In accordance with the goals of this work, the biofilm region comprises: (i) a set of continuous biomass voxels *connected* with the substratum, and (ii) all cavities within the biofilm structure *disconnected* from the liquid bulk. Note that this definition departs from the more conventional definition of biofilm as the set of biomass voxels attached to the substratum and enables a more refined voxel characterization. The top region includes all liquid bulk and floating biomass voxels, whereas the bottom region includes all substratum voxels and any other voxels detected below it. Here, the vertical window of interest is defined as the complete range of voxels in the vertical direction, fully including the biofilm region and a significantly reduced portion of all voxels in the top and bottom regions.

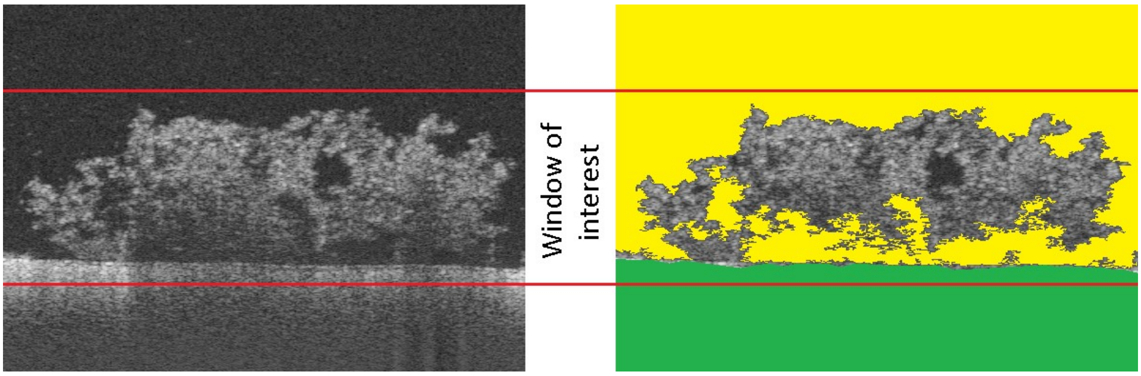

Figure A4: Vertical window of interest in OCT images of biofilms (2D illustration). The original grayscale image is shown on the left, and the top and bottom regions are marked in yellow and green, respectively, on the right. All pixels in the biofilm region retain their original grayscale intensities.

A3. Detailed Algorithm Descriptions

All algorithms developed in this work were coded as Python *functions* and included in the BISCAP code folder. This document highlights all functions in bold, and the corresponding source files are shown as underlined text (e.g. function **full_proc_p1** included in file processing_functions_3D.py). A summary of all code files is presented:

Table A1: Synopsis of BISCAP code files.

Code file	Description
aux_functions	Auxiliary functions for GUI
biscap_code	Full configuration of GUI
diagnostics_functions	Functions used during development for testing purposes
directions	Increments used in the calculation of neighbour voxels
full_processing_2D_single	Delivers full functionality for single 2D image
full_processing_3D_single	Delivers full functionality for single 3D image
parameters	Numeric and auxiliary parameters for all functions
processing_functions_2D	All functions related to 2D image processing
processing_functions_3D	All functions related to 3D image processing
processing_functions_bsi	All functions related to bottom interface detection
results_functions	Calculation of results: properties and images
set_up_functions	Auxiliary functions configuring main processing steps

In this Section, we expand on the working principles of all algorithms (as presented in the main manuscript) and provide more detailed information on their inner workings in articulation with these code files.

A3.1 Algorithm 1: Window of interest (pre-processing)

Per the discussion in Section A2, the first step in image processing consists of defining the *window of interest* along the vertical direction. This avoids processing a large number of voxels with no relevance for biofilm characterization and generally contributes to faster and more accurate image processing. A lower bound (z^{lb}) and an upper bound (z^{ub}) are defined for this purpose and *manually* set by users in the pre-processing screen of BISCAP, as illustrated in Figure A5.

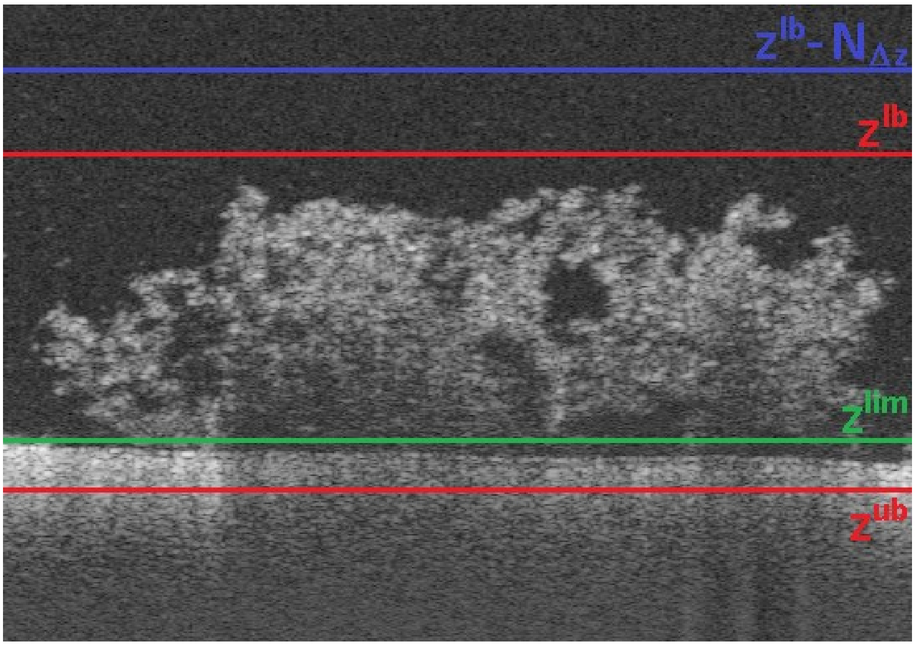

Figure A5: Graphical illustration of pre-processing in BISCAP.

An additional bound – z^{lim} – is also specified by users in this step. It is an auxiliary input enhancing the calculation of the bottom interface in Algorithm 2 and must be set such that <u>all voxels in the bottom region are *below* this bound</u>. In 3D images, users must check that the selected bounds are adequate for all slices at $y = 0, 1, \dots, N_y - 1$, according to the principles above.

The pre-processed image (I^{pre}) is obtained from the raw image (I^{raw}) by *trimming* it in the vertical direction between $z^{lb} - N_{\Delta z}$ and z^{ub}. Note that it is convenient that a small band of voxels in the top region immediately above the biofilm region are kept for

thresholding purposes. Parameter $N_{\Delta z}$ defines the length (distance in voxels) of this band. After setting all bounds, the raw image is trimmed automatically using function **img_trim** (set_up_functions.py).

A3.2 Algorithm 2: Bottom interface

The calculation of the bottom interface in 2D OCT images was thoroughly presented in Narciso et al. 2022. It is executed in three stages, as illustrated in Figure A6.

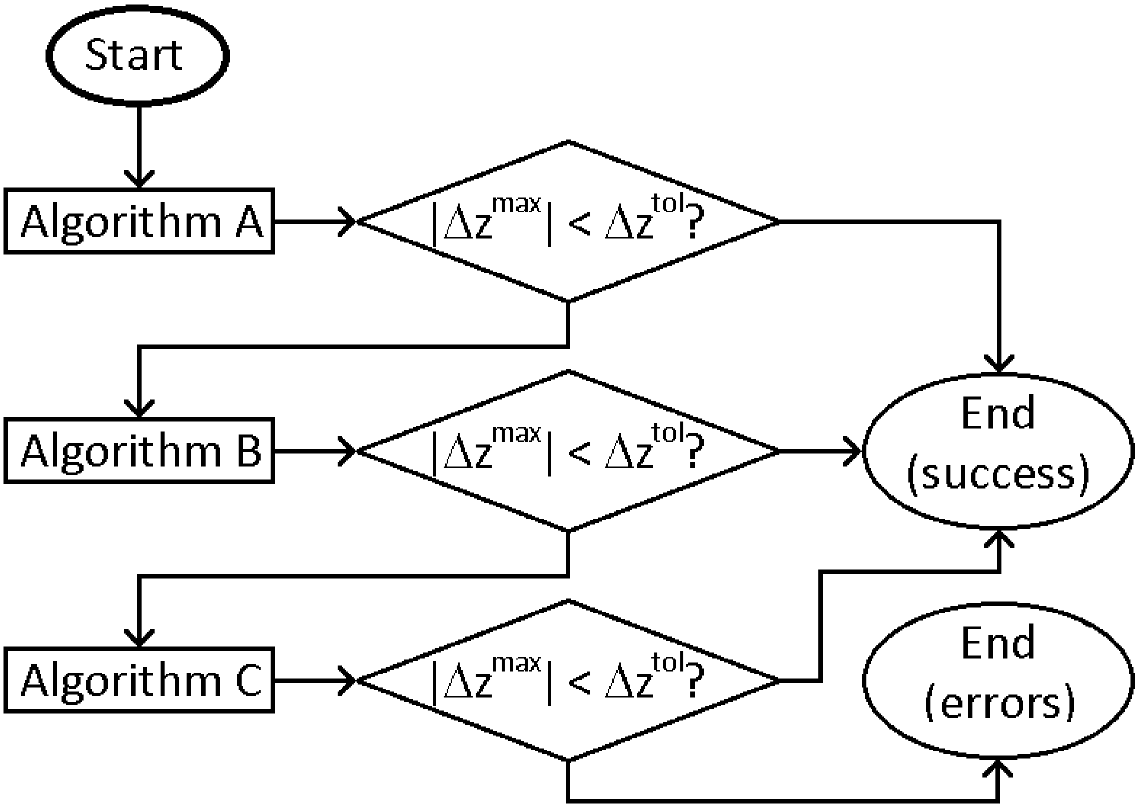

Figure A6: Top-level architecture for bottom interface estimation in 2D OCT images, where $|\Delta z^{max}|$ is the maximum step between consecutive positions of the bottom interface in the horizontal axis, and Δz^{tol} is a predefined error tolerance.

This detection strategy is repeated for all slices $y = 0,1, \ldots, N_y - 1$, from where a very accurate estimation of the bottom interface is generally obtained. A final smoothing step

is applied along the depth axis for all constant x slices. This strategy is very similar to Algorithm A, where a new set of discrete points based on the initial estimation of the interface is calculated. For all x slices, checking the vicinity of all discrete points along the depth axis allows any spurious points to be flattened towards the actual position of the bottom interface.

All code for bottom interface detection is included in the processing_functions_bsi.py file, where: (i) Algorithm 2 is coded in function **full_proc_double_axis_bsi**, (ii) the main interface detection engine – per slice – is coded in function **full_proc_single_axis_bsi**, and (iii) the final smoothing step is coded in function **secondary_axis_smoothing_bsi**.

A3.3 Algorithm 3: Threshold intensity

The threshold intensity may be manually set or calculated automatically. In the last case, a representative intensity of the top region (i^{void}) is obtained from a band of voxels just above the biofilm region (lower bound: $z^{lb} - N_{\Delta z}$, upper bound: z^{lb} – Figure A5). A calculation based on the p-percentile is made for this purpose using all (y, x) positions, from which an average value for i^{void} is then obtained. This calculation aims to establish a baseline intensity, such that all values above this intensity are a suitable threshold intensity: $i^{thresh} = i^{void} * m$, where $m > 1$ is a user parameter. The rationale for this approach is presented in greater detail in Narciso et al. 2022. Algorithm 3 is coded in function **threshold_calc** (set_up_functions.py).

A3.4 Algorithm 4: Voxel binarization

For all voxels in I^{pre}, if the grayscale intensity is lower/higher than the threshold intensity, the corresponding voxel is binarized as "0" (non-biomass) or "1" (biomass) using

classification matrix S^{bin} (same dimensions as I^{pre}). Algorithm 4 is coded in function **full_proc_p1** (processing_functions_3D.py).

A3.5 Algorithm 5: Biofilm structure

Algorithm 5 is coded in **full_proc_p2** (processing_functions_3D.py), which in turn uses a set of additional functions. Using classification matrix S^{str} (same dimensions as I^{pre}), it firstly initializes all voxels via function **init_p2** such that: (i) all biomass voxels are given a temporary *status* "0", and (ii) all non-biomass voxels given the temporary status "-1". The permissible space for continuity testing is the first group of voxels. At initialization, all biomass voxels immediately above the bottom interface define the first layer of biofilm voxels (status: "1"), from which continuity testing is executed.

The engine deriving this task is coded in function **cont_test_p2**; it may be applied over the full extent of S^{str}, or to any of its partitions. In any given iteration, it builds on the *current* set of biofilm voxels to identify any *unexplored* neighbour biomass/biofilm voxels in its vicinity, which are then included in a vector of voxels to be processed in the *next* iteration. A detailed discussion on voxel neighbours in 3D images is presented in Section A1. The algorithm proceeds over as many iterations as necessary until no new biofilm voxels are detected.

To speed up the continuity test, a parallel processing architecture was developed as follows:

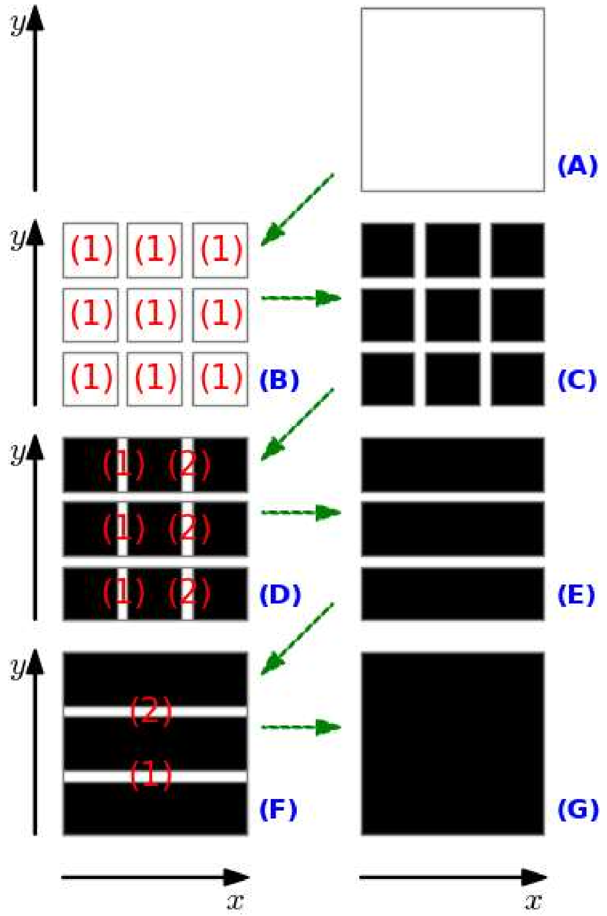

Figure A7: Parallel processing architecture. (A) Initialized classification matrix (full); (B) Matrix is partitioned in $y^{bands} * x^{bands}$ small chunks (illustrated with $y^{bands} = x^{bands} = 3$). A process is created per chunk to launch continuity testing simultaneously; (C) Partial processing of small chunks completed; (D) Classification matrix is partly restored from the small chunks to create a total of y^{bands} large chunks. One process is created per large chunk, and continuity is tested sequentially between their unprocessed edges; (E) Partial processing of large chunks completed; (F) Full classification matrix is restored from large chunks. A single process launches continuity testing sequentially between their unprocessed edges; (G) Classification matrix fully processed.

In the case of Algorithm 5, this set of operations is included in function **prl_proc_p2**, with the following function names: **units_input_calc_p2**, **units_proc**, **bands_input_calc_p2**, **bands_proc**, **mat_update_from_bands** and **link_bands_p2**.

Function **cont_test_p2** is used in **units_proc**, **bands_proc** and **link_bands_p2** to process the small chunks, the large chunks and the full matrix, respectively, as presented in Figure A7. In brief, when *binding* together all chunks via functions **bands_proc** and **link_bands_p2**, all unprocessed voxels between neighbour chunks are assessed: those which are biomass and include at least one neighbour biofilm voxel are added to a new initial biofilm layer, and then **cont_test_p2** is launched. Details on calculating these layers are available in functions **link_units_p2** and **link_bands_p2**.

Upon conclusion of continuity testing, all voxels initialised with status "-1" are updated to status "0" (not biofilm) via function **final_proc_p2**. As a result, at the end of this stage, all voxels in S^{str} are assigned one of two possible statuses: (i) "1" – biofilm, or (ii) "0" – non-biofilm.

A3.6 Algorithm 6: Top interface

In earlier work, the initial layer used to begin continuity testing across the top region was set at z^{lb} (consistently with Figure A5). This approach ensures, as required, that all biofilm voxels are below this initial layer. In biofilms with high rugosity, this approach leads to the processing of large portions of the top region, as illustrated below:

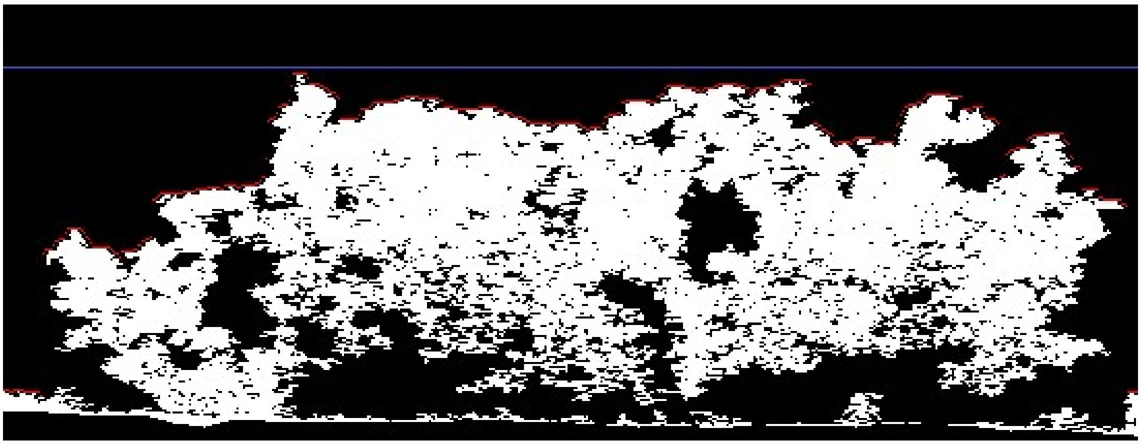

Figure A8: Setting the initial layer to launch continuity testing in the top region. In earlier work, this is represented by the flat blue line at z^{lb}. Now, this layer is defined *just above* the biofilm region (information available after Algorithm 5 terminates) and represented by the set of red pixels. This approach avoids the unnecessary processing of large portions of the top region and contributes to algorithm efficiency.

This work introduced an improvement whereby this initial layer is set much closer to the biofilm region. For all (y, x) it suffices to set the initial layer slightly above the highest biofilm voxel at (y, x). In fact, this calculation is based on all neighbour (y, x) positions, which *widens* the layer. This adaptation is necessary for the method's generality, namely to reach all voxels at the top interface. This calculation is conveniently executed after biofilm thickness is calculated in function **thickness_calc** (results_functions.py).

Algorithm 6 is coded in **full_proc_p3** (processing_functions_3D.py), which in turn uses a set of additional functions. Using classification matrix S^{int} (same dimensions as I^{pre}), it firstly initializes all voxels via function **init_p3** such that:

- All voxels above the initial layer are given the status "-1" (top region – not interface).

- All voxels at and below the bottom interface are given the status “3” (bottom region).
- All voxels at the initial layer are given the temporary status “-3” (these are in the top region, but undetermined at this stage if they belong to the top interface).
- All remaining voxels are initialised with the temporary status “0”.

The last set of voxels defines the permissible space for continuity testing. During continuity testing, voxels are given their final statuses:

- “-1”: Voxels at the top region with no neighbour biofilm voxels.
- “1”: Voxels at the top interface (top region side)
- “2”: Voxels at the top interface (biofilm region side)

The parallel processing architecture, as depicted in Figure A7, is implemented in this Algorithm via function **prl_proc_p2**, which in turn uses the following functions: **units_input_calc_p3**, **units_proc**, **bands_input_calc_p3**, **bands_proc**, **mat_update_from_bands** and **link_bands_p3**.

Given that the classification of all voxels in one of the possible statuses presented above requires all neighbour voxels to be checked, this presents a challenge in processing both the small and large chunks presented in Figure A7. This is illustrated in Figure A9:

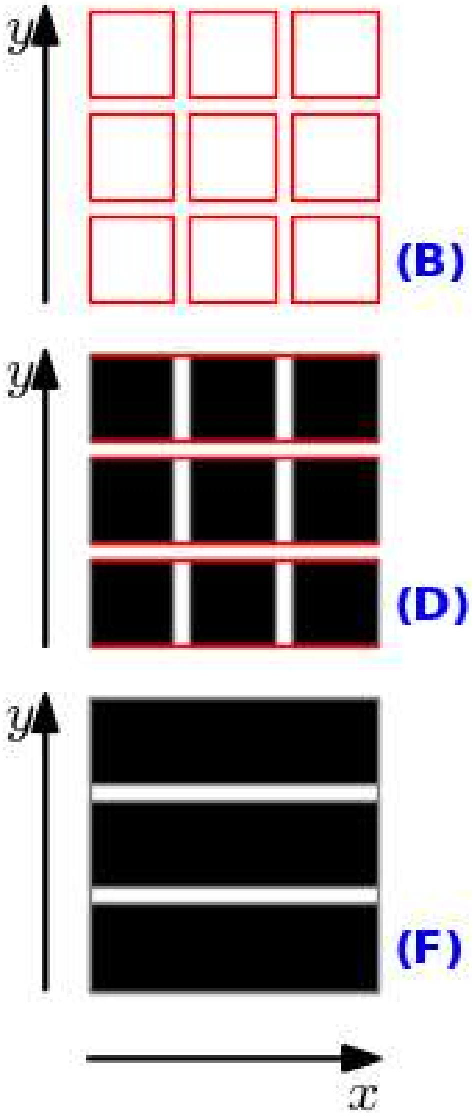

Figure A9: Parallel processing in the top region – adapted from Figure A7. (B) While small chunks are processed in parallel, note that the rigorous classification of their voxels at all edges (in red) requires information from all voxels at unprocessed neighbour planes (to check if they are included in the top interface) and which is not available at this stage. In this case, the continuity engine is tailored to exclude these edges from full classification; if any voxel in the top region is found at an edge, it is given the temporary status "-3", and processing resumes from these edges at a later stage. (D) The same problem occurs in large chunks but only at the constant *y* edges, and the continuity engine is tailored accordingly. (F) In the full matrix, none of the restrictions above is applicable.

As a result, three slightly different implementations of the continuity engine in the top region are implemented and used as follows in the following functions:

- **units_proc_prl**: uses engine **cont_test_p3_excl_y_x_edges** (small chunks)
- **link_units_p3**: uses engine **cont_test_p3_excl_y_edge** (large chunks)
- **link_bands_p3**: uses engine **cont_test_p3_full** (full matrix)

All variations of **cont_test_p3** work on the same principles as **cont_test_p2**, except that, in this case, continuity is tested across the set of voxels in the top region.

Binding together all chunks is implemented via functions **bands_proc** and **link_bands_p2**, where all voxels at their edges are assessed: those with status "-3" are added to a vector defining a new starting layer and the relevant continuity engine applied as presented above.

Upon conclusion of continuity testing, all voxels with status "1" in S^{int} define the top interface (z^{top}). The set of all biofilm voxels neighbour to at least one voxel in the top region is also calculated if required; these correspond to the set of voxels at the top interface at the *biofilm region side*, while those in z^{top} are the top interface voxels at the *top region side*. Their calculation is likely less important than those voxels at z^{top} and disabled by default; it may be enabled in the automatic processing options in BISCAP.

A final processing task is performed on S^{int} via function **full_proc_p4**, where all voxels previously classified as top region ("-1"), top interface ("1") and support (3) are all given their final status "0" (not biofilm region). This way, S^{int} is such that voxels in and out of the biofilm region have statuses "1" and "0", respectively.

A4. Algorithms implementation validation

Algorithms 1 and 4 require trivial code implementations. A thorough discussion on the ability of Algorithm 2 to capture the bottom biofilm interface was presented in Narciso et al. 2022; furthermore, for any image of interest, it is possible to visually validate the estimated interface from algorithmic calculations against the actual interface (which is generally easily identifiable). The merits of Algorithm 3 in delivering a meaningful threshold intensity were also discussed in Narciso et al. 2022; the calculation of thresholds is an image processing simplification towards the calculation of the biofilm structure, and one cannot, in rigour, specify what the exact threshold intensity value is. As a result, a formal validation of Algorithms 1-4 does not make sense in this context.

Algorithms 5 and 6, on the other hand, are rather complex and computationally intensive. The delivered image processing outputs do not enable any sort of confirmation that their implementations are correct. The only route towards this end is to check that the results from *automatic* processing match those from some sort of independent *manual* processing route. Given that the size of 3D images can easily be larger than 10^8, checking the results via individual voxel processing would be impractical at best, if not impossible. A distinct approach is employed here: three 3D images were artificially crafted, each comprising a set of biofilm voxels (high intensities) and a set of "holes" within their structures (disconnected from the liquid bulk); these are the set of void voxels (low intensities) contributing to biofilm porosity within their structures. All volumes created have a very well-defined structure, enabling a much simpler route to account for the complete set of voxels in 3D images. In other words, matrices S^{str} and S^{int} as calculated via Algorithms 5 and 6, respectively, can be delivered independently using manual calculations to test the accuracy of algorithm implementation. All images are available for detailed analysis in https://web.fe.up.pt/~fgm/biscap3d/ (Validation Examples).

A4.1 Cube

The blueprints for this 3D image are depicted in Figure A10. This is a 200*200*200 voxels image, where a cube of biofilm at the centre of the image was artificially created.

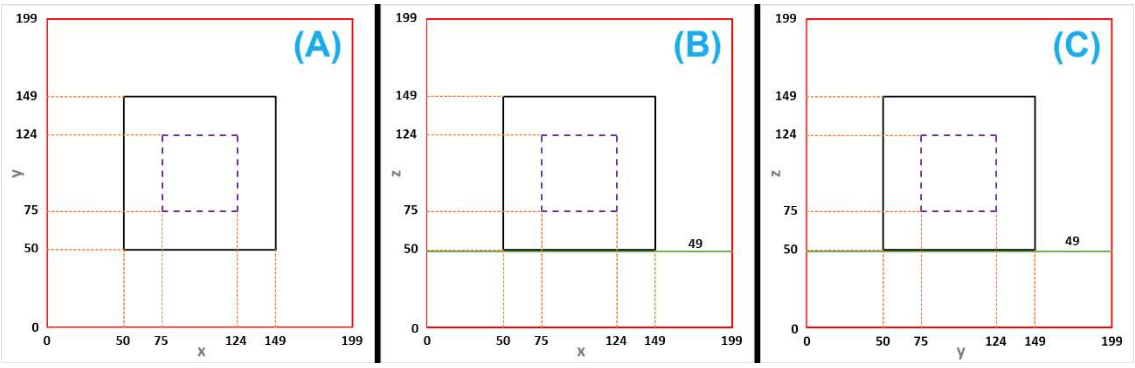

Figure A10: Blueprints of biofilm cube. (A) Top, (B) Front, (C) Side views.

All voxels in this image are black (grayscale intensity equals 0), except:

1) All voxels within the cube bounded by the solid black lines – except those within the cube bounded by the dotted purple lines – where the grayscale intensity is assigned to 200.
2) All voxels at $z = 49$ (voxels marked as green in Figure A10 (B) and (C)) where the grayscale intensity is assigned to 255 (position of the bottom interface).

This 3D image is an artificial construction of a biofilm cube, where a cube of void voxels is included within its structure (bounded by the purple lines). The boundaries of all cubes are explicitly defined in Figure A10.

<u>Voxels in the biofilm structure</u>

Delivering the full matrix S^{int} would be impractical. The calculation of the total number of voxels included in the biofilm structure, however, is a simple calculation: from Figure A10, given that this structure is a cube of size 100, the total number of voxels fitting this criterion amounts to $100^3 = 10^6 = \sum S^{int}$. Note that the biofilm structure also includes all void voxels in the innermost cube (disconnected from the liquid bulk).

Biofilm voxels

The total number of void voxels within the biofilm structure is $50^3 = 1.25*10^5$. Therefore, the total number of biofilm voxels is 10^6 - $1.25*10^5 = 8.75*10^5 = \sum S^{str}$.

Biofilm thickness series

In the centre of the (x, y) range displayed in Figure A10 (A), biofilm thickness matches the thickness of the biofilm cube: 124 - 25 + 1 = 100 (bottom interface at $z = 49$). In the rest of the (x, y) range the biofilm thickness is 0, since no more biofilm voxels are defined in this image. The thickness profile is graphically illustrated in Figure A11.

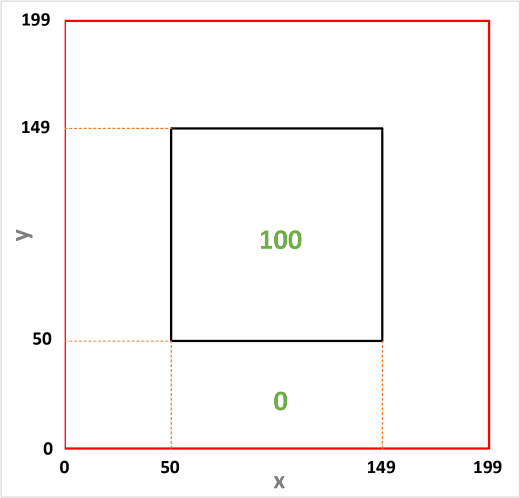

Figure A11: Thickness profile for first artificial 3D image (cube).

Formally, BISCAP delivers biofilm thickness and roughness in μm, where parameter vx_{len} converts distance expressed in number of voxels to μm. This conversion is dismissed in manual calculations; to ensure an adequate comparison between the manual and automatic routes, parameter vx_{len} must be set to 1 in BISCAP (Section A4.4).

All structural properties are calculated in sequence for this 3D image (via Equations 1 – 5, as presented in the main manuscript).

Structural properties

$$\overline{\mathbf{L_F}} = \frac{100 * (100 * 100) + 0 * (200 * 200 - 100 * 100)}{200 * 200} = \mathbf{25}$$

$$\boldsymbol{R_\alpha} = \frac{|100 - 25| * 100 * 100 + |0 - 25| * (200 * 200 - 100 * 100)}{200 * 200} = \mathbf{37.5}$$

$$\boldsymbol{R^*_\alpha} = \frac{37.5}{25} = \mathbf{1.5}$$

$$\boldsymbol{C_P} = \frac{8.75 * 10^5}{100 * (100 * 100) + 0 * (200 * 200 - 100 * 100)} = \mathbf{0.875}$$

$$\mathbf{\Phi} = 1 - \frac{8.75 * 10^5}{10^6} = \mathbf{0.125}$$

A4.2 Polyhedron

The blueprints of this 3D image comprising 200*250*200 voxels are depicted below.

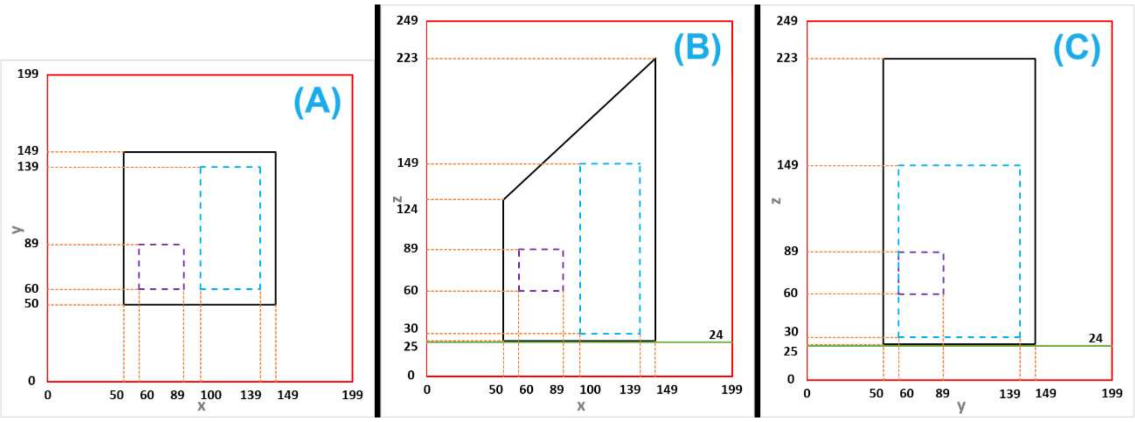

Figure A12: Blueprints of biofilm polyhedron. (A) Top, (B) Front, (C) Side views.

All voxels in this image are black, except:

1) All voxels within the polyhedron bounded by the solid black lines – except those within: (i) the cube bounded by the dotted purple lines, and (ii) the cuboid bounded by the dotted blue lines – where the grayscale intensity is assigned to 200.
2) All voxels at $z = 24$ (voxels marked as green in Figure A12 (B) and (C)) where the grayscale intensity is assigned to 255 (position of the bottom interface).

This biofilm polyhedron includes two “holes” of void voxels in its structure; the bounds of all volumes are explicitly defined in Figure A12.

Voxels in the biofilm structure

This artificial biofilm structure is a combination of a cube of size 100, and a triangular cylinder immediately above the cube with dimensions 99*99*100. Therefore, the total number of voxels in the biofilm structure amounts to 100^3 + (99 + 98 + … + 1)*100 = 1.495*10^6 = $\sum S^{int}$.

Biofilm voxels

The total number of void voxels within the “holes” bounded by the purple and blue lines are 30^3 = 2.7*10^4 and 40*80*120 = 3.84*10^5, respectively. The total number of biofilm voxels is 1.495*10^6 - 2.7*10^4 - 3.84*10^5 = 1.084*10^6 = $\sum S^{str}$.

Biofilm thickness series

The thickness profile is graphically depicted in Figure A13.

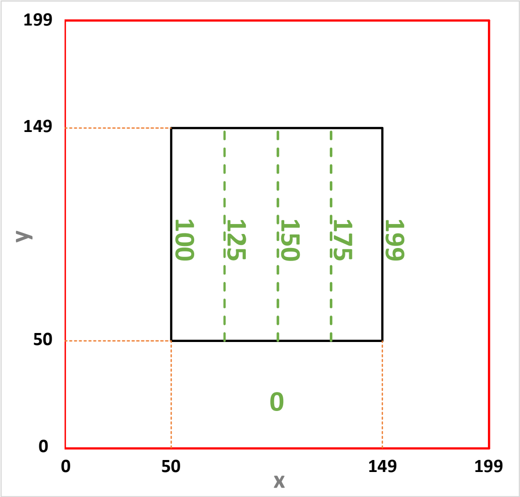

Figure A13: Thickness profile for second artificial 3D image (polyhedron).

In the centre of the (x, y) range where the polyhedron is located – displayed in Figure A12 (A) – biofilm thickness ranges from 100 (124 – 25 +1) at x = 50 to 199 (223 – 25

+1) at $x = 149$. This profile is clearly visible in Figure A12 (B). In the rest of the (x, y) range the biofilm thickness is 0, since no more biofilm pixels are defined. All structural properties are calculated below:

<u>Structural properties</u>

$$\overline{\mathbf{L_F}} = \frac{(100 + 101 + \cdots + 199) * 100 + 0 * (200 * 200 - 100 * 100)}{200 * 200} = \mathbf{37.375}$$

$$\boldsymbol{R_\alpha} = \frac{(|100 - 25| + \cdots + |199 - 25|) * 100 + |0 - 25| * (200 * 200 - 100 * 100)}{200 * 200} = \mathbf{56.0625}$$

$$\boldsymbol{R_\alpha^*} = \frac{56.0625}{37.375} = \mathbf{1.5}$$

$$\boldsymbol{C_P} = \frac{1.084 * 10^6}{(100 + 101 + \cdots + 199) * 100 + 0 * (200 * 200 - 100 * 100)} = \mathbf{0.72508}$$

$$\mathbf{\Phi} = 1 - \frac{1.084 * 10^6}{1.495 * 10^6} = \mathbf{0.27492}$$

A4.3 Mushroom

This is a more complex image aiming to capture the *mushroom*-like shapes often found in biofilm image processing. It consists of a central "tower" and four protruding "arms" of biofilm voxels along the x and y directions. The image also includes three "holes" within its structure fully disconnected from the liquid bulk and thus contributing to biofilm porosity. The blueprints are presented in Figure A14. For detailed analysis of this shape the corresponding 3D image may be consulted in the "examples" file.

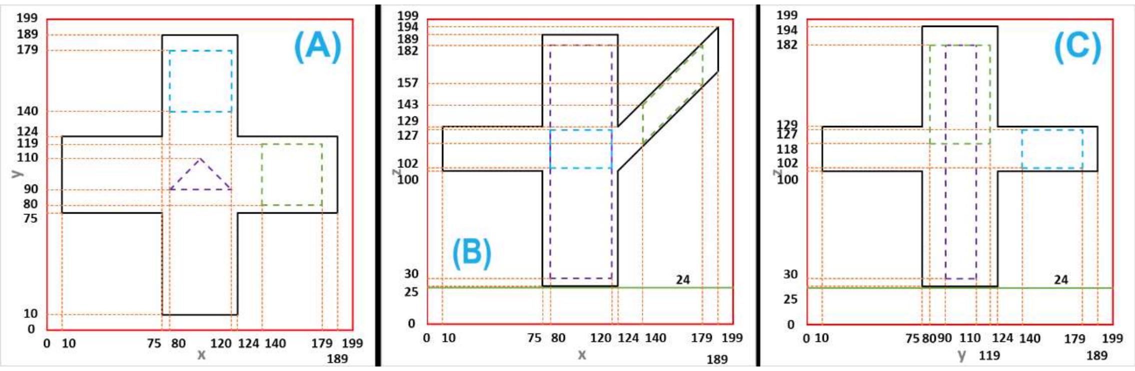

Figure A14: Blueprints of biofilm mushroom. (A) Top (B) Front, (C) Side views.

All voxels in this image are black, except:

1) All voxels within the polyhedron bounded by the solid black lines – except those within: (i) the triangular cylinder bounded by the dotted purple lines, (ii) the cuboid bounded by the dotted blue lines, and (iii) the parallelepiped bounded by the dotted green lines – where the grayscale intensity is assigned to 200.
2) All voxels at $z = 24$ (voxels marked as green in Figure A14 (B) and (C)) where the grayscale intensity is assigned to 255 (position of the bottom interface).

This biofilm mushroom includes three "holes" of void voxels in its structure; the boundaries of all volumes are explicitly defined in Figure A14.

Voxels in the biofilm structure

This artificial biofilm structure is a combination of a cuboid of size 50*165*50 ("tower") the three cuboids of size 50*30*65 (horizontal "arms") and a parallelepiped of size 50*30*65 (single inclined "arm"). The total number of voxels in the biofilm structure amounts to 50*165*50 + 4*50*30*65 = 8.025*10^5 = $\sum S^{int}$.

Biofilm voxels

The total number of voxels in the "holes" bounded by the purple, blue and green lines is 21*21*153 = 67473, 41*40*26 = 46240 and 40*40*26 = 41600, respectively. The total number of biofilm voxels is 8.025*10^5 - 67473 - 46240 - 41600 = 650787 = $\sum S^{str}$.

Biofilm thickness series

The thickness in the centre of the (x, y) range where the "tower" is included is 165 (189-25+1). Thickness at the horizontal "arms" is 105 (129 – 25 + 1). Thickness at the inclined "arm" ranges from 106 at $x = 125$ to 170 at $x = 189$. In the rest of the (x, y) range no

biofilm pixels are defined, and the thickness is 0. The thickness profile is graphically illustrated in Figure A15.

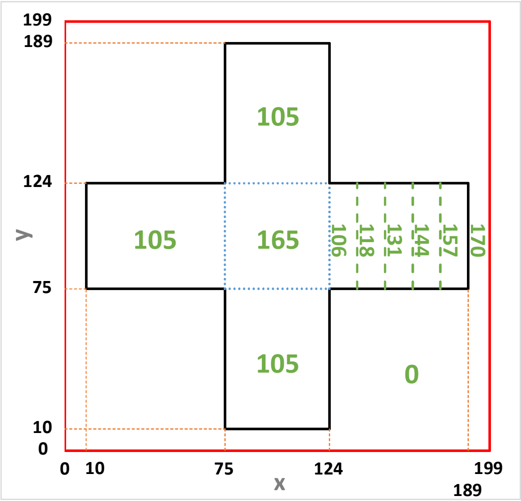

Figure A15: Thickness profile for third artificial 3D image (mushroom).

All structural properties are calculated below:

Structural properties

$$\overline{\mathrm{L_F}} = \frac{165 * (50 * 50) + 105 * (65 * 50) * 3 + (106 + 107 + \cdots + 170) * 50}{200 * 200} = \mathbf{47.11875}$$

$$\boldsymbol{R_a} = \frac{|165 - \overline{\mathrm{L_F}}| * (50 * 50) + |105 - \overline{\mathrm{L_F}}| * (50 * 65) * 3 + \left(|106 - \overline{\mathrm{L_F}}| + \cdots + |170 - \overline{\mathrm{L_F}}|\right) * 50}{200 * 200}$$

$$+ \frac{|0 - \overline{\mathrm{L_F}}| * (200 * 200 - 50 * 50 - 65 * 50 * 4)}{200 * 200} = \mathbf{57.72047}$$

$$\boldsymbol{R_a^*} = \frac{57.72047}{47.11875} = \mathbf{1.225}$$

$$\boldsymbol{C_P} = \frac{650787}{165 * (50 * 50) + 105 * (65 * 50) * 3 + (106 + 107 + \cdots + 170) * 50} = \mathbf{0.345291}$$

$$\boldsymbol{\Phi} = 1 - \frac{650787}{8.025 * 10^5} = \mathbf{0.18905}$$

A4.4 Automatic processing and validation

The three artificial 3D images presented in the previous Sections are included in https://web.fe.up.pt/~fgm/biscap3d/ (Validation Examples). The following steps are recommended for a consistent comparison with results obtained from manual processing. Check the user manual for instructions on using BISCAP.

1) Pre-process all images in BISCAP (meaningful definition of bounds).
2) Set the threshold calculation to manual and set its value to 80 (any value below 200 – all biofilm voxels in the 3D images were assigned this intensity).
3) Set vx_{len} to 1; since thickness and roughness are expressed in voxel distance units in manual calculations, this setting is mandatory for consistency.
4) Disable the calculation of topography in the options of the automatic processing screen (in these images, an incompatibility between the numpy and the matplotlib libraries prevents these images from being saved correctly; this is not a critical problem in the general context of biofilm image processing and will be addressed in the next release of BISCAP).
5) Run automatic processing and check the structural parameters directly on BISCAP or in the saved Excel "outputs.xlsx" file upon conclusion of this activity.

These steps were executed for all 3D images, and the same results as reported from manual processing in Sections A4.1-A4.3 were consistently delivered. This provides a strong indication that the implementation of Algorithms 5 and 6 is fully consistent with the technical specifications presented in Section A3.

A5. Default algorithms' parameters

All parameters have been empirically optimized to deliver accurate results to the complete set of biofilm images used during development and testing. All internal parameters are defined and set in the file parameters.py. Users are recommended not to change these, unless for some specific development tasks. The key user defined parameters and their default values are presented in Table A2:

Table A2: Default values for algorithms' parameters.

**Parameter**	**Default**
p	60
m	1.4
vx_{len}	2.08333µm **Note 1**
x^{bands}	5
y^{bands}	5

Note 1: The default for voxel length is such that 100 voxels equal 48µm. This parameter must be configured to match the local image acquisition specifications.
