# Supplementary material for: 3D Optical Coherence Tomography image processing in BISCAP: characterization of biofilm structure and properties
Source: Bioinformatics. 2024 Jan 23;40(2):btae041. doi: 10.1093/bioinformatics/btae041 (PMC10868339; doi:10.1093/bioinformatics/btae041)
Supplement: btae041_Supplementary_Data [file btae041_supplementary_data.zip › user_manual.pdf]

Biofilm Imaging and Structure Classification Automatic Processor (BISCAP): User Manual

Version 2 (January 2024)

Disclaimer

The supplied version of the software has been tested, and no undesired behaviour/interactions with the Windows operating system have been detected. Depending on the size and complexity of the images being processed, automatic processing may require a significant utilization of available processing resources, particularly in 3D images. Users are made aware that BISCAP automatically reads and writes a set of images and Excel files to disk within the reference root folder(s) specified by users. The authors shall not be liable for any damage caused by using this software.

Authors

All Python functions and the architecture of BISCAP were devised, written, and tested by Diogo A. C. Narciso and Manuel Monteiro. The architecture of BISCAP was further refined, fully implemented, and tested by Nuno O. Dias as a Graphical User Interface (GUI).

U. PORTO
FEUP FACULDADE DE ENGENHARIA
UNIVERSIDADE DO PORTO
ALiCE
Lepabe
Laboratory for Process Engineering, Environment, Biotechnology and Energy

Table of Contents

Disclaimer 1

Authors 1

Table of Contents 2

Development Notes 3

Definitions 3

Nomenclature 4

1. Introduction 5

2. BISCAP set-up 6

- **2.1 Installation** 6
- **2.2 Launching BISCAP** 7
- **2.3 Troubleshooting** 8

4. Pre-processing 11

5. Automatic Processing 14

6. Post-processing 18

- **6.1 2D Post-processing** 18
- **6.2 3D Post-processing** 20

7. Single Image Mode 2D 23

8. Multi-images Mode 2D 24

- **8.1 Pre-processing** 24
- **8.2 Automatic Processing** 25
- **8.3 Post-processing** 26
- **8.4 Workflow** 27

9. Single Image Mode 3D 28

10. Tutorial 1: Single Image Mode 29

11. Tutorial 2: Multi-images Mode 2D 32

12. Tutorial 3: Single Image Mode 3D 36

Development Notes

The main new feature of the 2nd version of BISCAP is the ability to process 3D images. Earlier, BISCAP processed 2D vertical image slices only. This is a significant development enabling the complete assessment of the complex morphologies of biofilm structures. Two continuity models (deep and shallow) were developed to estimate all pixels/voxels at the top interface.

A new and faster strategy for continuity testing is now implemented. Algorithms based on continuity testing are the most expensive, and to further speed-up calculations, a parallel processing architecture was also put in place. Both factors contribute to faster computation times; 2D/3D images of standard sizes are fully processed in a few seconds/minutes.

New image outputs were developed for 3D images, including a 3D representation of the topography of biofilms. Users may also examine 2D slices along the three directions of 3D images. The calculation of porosity and two new auxiliary structural parameters were implemented.

The 2nd version of BISCAP is now delivered as a set of Python code files (instead of a large standalone file). This option enables BISCAP to load more quickly and allows interested users to read all code. This requires the prior installation of all necessary Python components to enable all functionalities in BISCAP.

Some minor bugs were corrected.

Definitions

<u>2D OCT image:</u> 2-dimensional image obtained from an OCT scan along the vertical axis (z) and the horizontal axis (x), and commonly referred to as a B-scan. 2D OCT images include a total of N_z and N_x discretized positions in the vertical and horizontal axes, respectively. They include a total of $N_z * N_x$ pixels, where each pixel is associated with a unique grayscale intensity.

<u>3D OCT image:</u> 3-dimensional image obtained from an OCT scan along the depth axis (y), the vertical axis (z), and the horizontal axis (x), and commonly referred to as a C-scan. These correspond to a collection of N_y 2D OCT images along the depth axis. They include a total of $N_y * N_z * N_x$ voxels, where each voxel is associated with a unique grayscale intensity.

<u>Background:</u> All pixels/voxels in 2D/3D OCT images with grayscale intensities below a pre-defined threshold intensity.

<u>Biofilm:</u> All biomass pixels/voxels defining a continuous set of pixels/voxels extending from the bottom interface.

<u>Biofilm region:</u> Set of all biofilm pixels/voxels and all background pixels/voxels with no continuity to the top region (cavities in biofilms).

<u>Biomass:</u> All pixels/voxels in 2D/3D OCT images with grayscale intensities above a pre-defined threshold intensity.

<u>Bottom interface:</u> Set of pixels/voxels identified from automatic processing as the substratum, thus defining the boundary between the biofilm and bottom regions. These

are generally associated with the positions where the highest grayscale intensity along the vertical direction is detected.

Bottom region: Set of all pixels/voxels at and below the bottom interface.

Grayscale intensity: All pixels/voxels in 2D/3D OCT images are assigned a grayscale intensity ranging from 0 (black) to 255 (white).

OCT: Optical Coherence Tomography.

Pixel: The smallest element of 2D OCT images, in accordance with the definition of 2D OCT image.

Substratum: The surface where biofilm is attached to.

Threshold intensity: Pre-defined or calculated grayscale intensity such that all pixels/voxels (in 2D/3D images) with grayscale intensities above/below this value are classified as biomass/background, respectively.

Top interface (biofilm region side): Set of all biofilm pixels/voxels at the boundary between the biofilm and top regions.

Top interface (top region side): Set of all background pixels/voxels at the boundary between the biofilm and top regions.

Top region: Set of all background pixels/voxels enveloping the biofilm region and all floating biomass pixels/voxels.

Voxel: The smallest element of 3D OCT images, in accordance with the definition of 3D OCT image.

Nomenclature

A distinct word formatting is used in this document to distinguish between the elements of BISCAP, as follows:

- Screens: **bold**
- User-defined parameters: *italics*
- Actionable buttons: underline arial
- Files and folders: “under brackets”
- Code strings: `consolas`

1. Introduction

BISCAP is a Graphical User Interface (GUI) enabling the detailed analysis of 2D and 3D biofilm images obtained via OCT. This includes calculating biofilm structural parameters and delivering other useful visual outputs. This work is an extension to the first version of BISCAP developed specifically for the automatic processing of 2D OCT biofilm images (January 2022). All processing functions and BISCAP have been extended also to accommodate the 3D case. This manual covers the utilization of BISCAP on 2D and 3D OCT biofilm images.

This work was performed to comply with two fundamental guiding principles. Firstly, all image processing tasks should be made as automatic as possible, thus generally reducing the time required to obtain the information of interest and with the least possible burden. Secondly, given the intrinsic subjectivity reported in the field on threshold selection, automatic functions were developed to remove subjectivity as much as possible from image processing.

The development work comprised two distinct stages. Firstly, a set of Python functions were developed, tested, and improved to deliver the proposed goals. This work consisted mainly of code writing and testing its performance on a large set of 2D and 3D OCT images. In the second stage, all code was built into a GUI, of which BISCAP is the main deliverable. This format is particularly convenient to researchers not familiar with Python since BISCAP does not require any programming knowledge. Instead, all tasks are managed and executed via intuitive screens and buttons. This manual explicitly covers the practical aspects of the utilization of BISCAP. All functions are available as readable code, and additional details on their implementation may be found in Appendix 1.

2. BISCAP set-up

BISCAP is delivered as a collection of Python code files. Any Python platform may be used to read these files and launch the GUI. In this manual, we suggest utilizing the Anaconda platform, and specifically the Anaconda Prompt, to launch the GUI. All new users are recommended to read the full contents of this Section before exploring the image processing features of BISCAP.

2.1 Installation

The necessary installation steps are listed below:

1) Access the www.anaconda.com website and download the latest version of Anaconda - choose the version that best suits your operating system.[1]
2) Install Anaconda, accepting all default installation options.
3) Unzip and save the BISCAP files from https://github.com/diogonarciso/BISCAP to a folder of your choice (the folder structure must not be changed).
4) Open Anaconda. In the left sidebar of the window, click on Environments:[2]

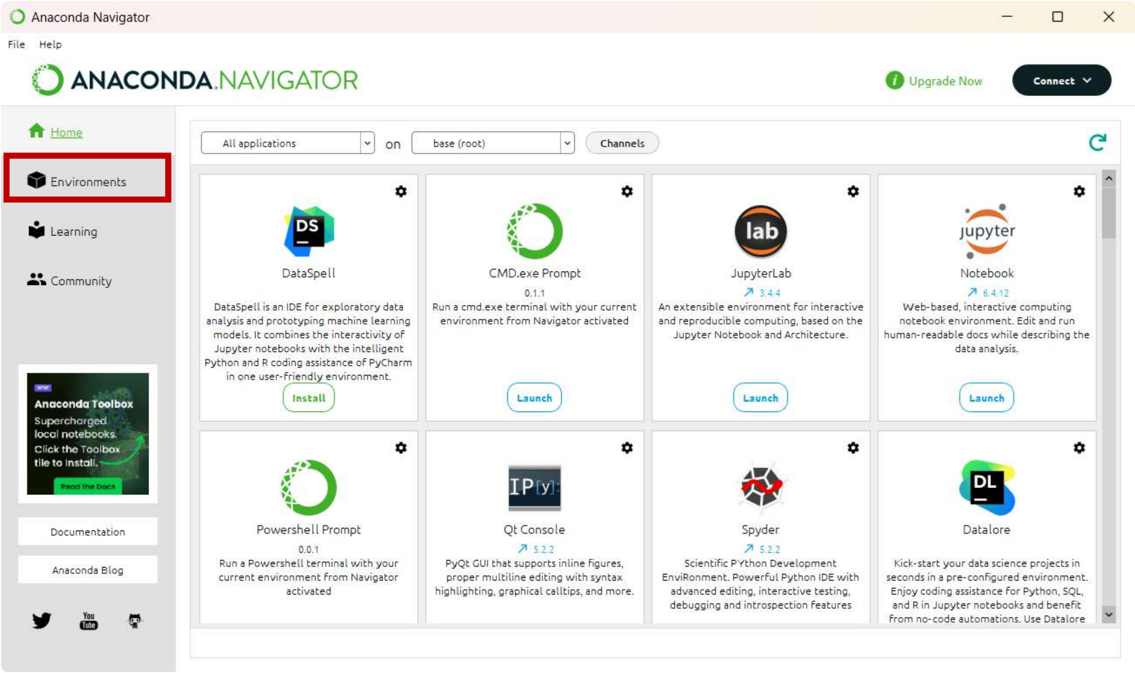

Figure 1: Anaconda navigator – homepage.

5) Follow the steps depicted in Figure 2: (1) press the Import button, and (2) the folder icon. Then, (3) browse to the location where the BISCAP project files were saved and select the “environment\biscap_env.yaml” (4). Open this file (5) and click the second (green) Import button (6). These steps create a new environment in Anaconda using all library versions used in the development of BISCAP. This step avoids the manual installation of libraries and ensures that the GUI behaves as designed. Downloading and installing all libraries can take a few minutes.

[1] BISCAP was developed for Windows operating systems.

[2] Environments are used in Anaconda to manage libraries in project development.

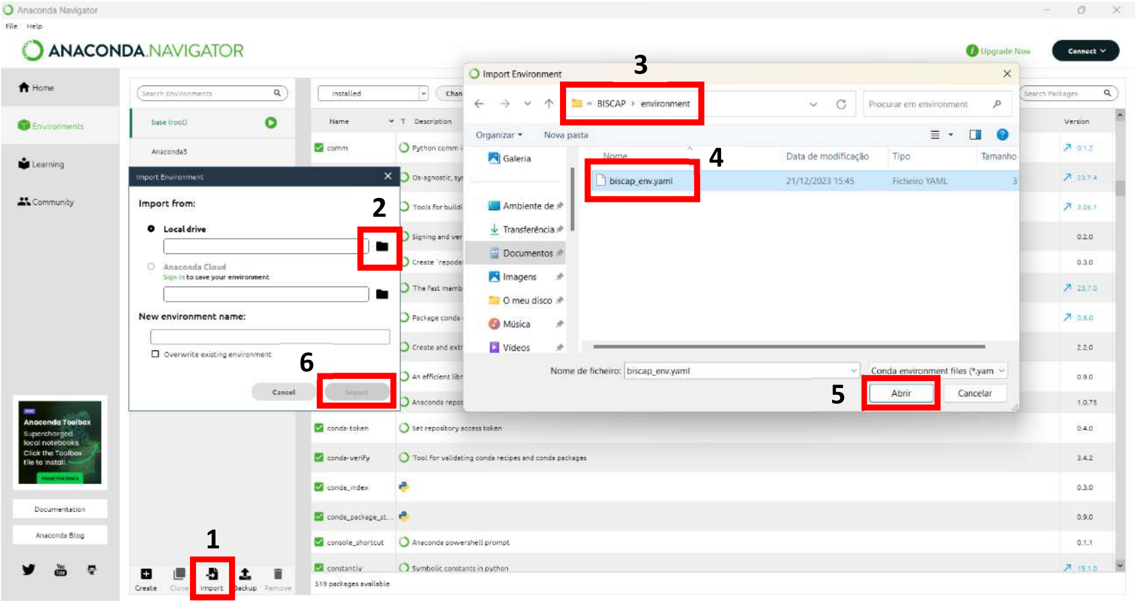

Figure 2: Anaconda navigator – importing an environment.

6) Close the Anaconda navigator. The installation is complete.

2.2 Launching BISCAP

Anaconda Prompt is a command line application which is installed with Anaconda and may be opened from the Start or Search menus in Windows. The following steps are suggested to launch BISCAP:

1) Open the Anaconda Prompt.
2) Type or copy-paste the following lines of code to Anaconda Prompt (the code below illustrates the case when the project folder is saved to the Desktop):
   a. `conda activate biscap_env`
   b. `cd Desktop\BISCAP_v2`
   c. `python biscap_code.py`

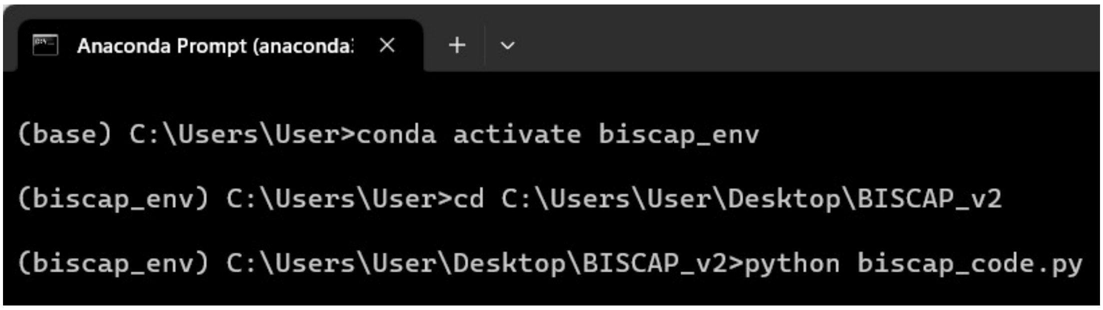

Figure 3: Launching BISCAP from Anaconda Prompt.

The first command activates the defined environment, including all supporting libraries. The second command sets the working path to the folder's location, including all BISCAP files. The example above illustrates when this folder is saved on the Desktop, and users must make any necessary adaptations consistently with step 3 of the installation. The third

command launches BISCAP via the "biscap_code.py" file in the project folder. To exit BISCAP, it suffices to close Anaconda Prompt.

2.3 Troubleshooting

The proposed methodology for library installation was devised to be as simple as possible. On launching BISCAP for the first time, users must check that: (i) the GUI is launched (Figure 6), and (ii) no errors are reported in the Anaconda Prompt. If any library is missing in the environment, this will be flagged in the Anaconda Prompt:

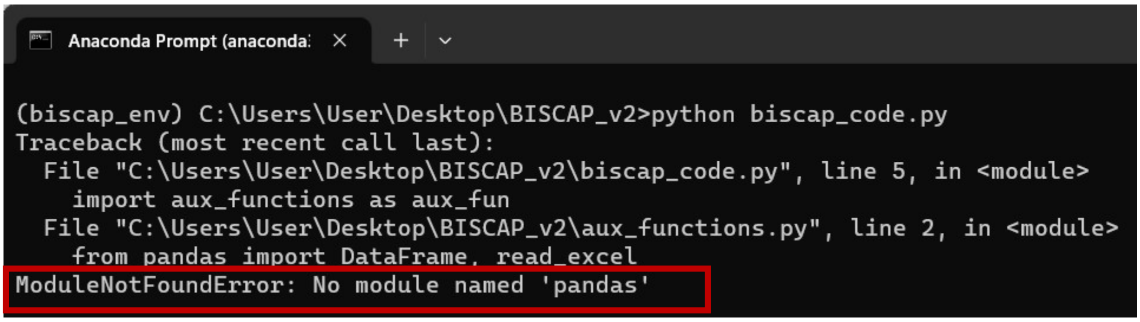

Figure 4: Troubleshooting missing libraries: illustrating the case when the pandas library is not installed on the environment.

It is not expected that this kind of error will occur given that the proposed installation steps should install all necessary libraries. If such an error is detected, it suffices to install any missing libraries in the Anaconda Navigator. If any error or bug is detected by users, these will also be displayed in the Anaconda Prompt. If this occurs, please do send a list of relevant steps leading to the problem and a print screen of the command line.

The authors plan to release new versions of BISCAP in the future to fix any reported bugs and ensure that the BISCAP code remains fully compatible with all supporting libraries. Any comments or suggestions are welcome.

3. Basics

BISCAP was designed to process 2D and 3D OCT biofilm images automatically. More concretely, these must be grayscale images in tiff (2D and 3D) or png (2D) formats obtained from OCT scans. An example is shown in Figure 5, where a 2D biofilm image is presented in its more conventional form (left), and an equivalent matrix representation highlighting its data structure is also shown (right). The same principles apply to 3D images, which comprise multiple image slices on consecutive positions of the depth axis (y). All processing steps in BISCAP are executed by automatically manipulating these 2D and 3D matrices.

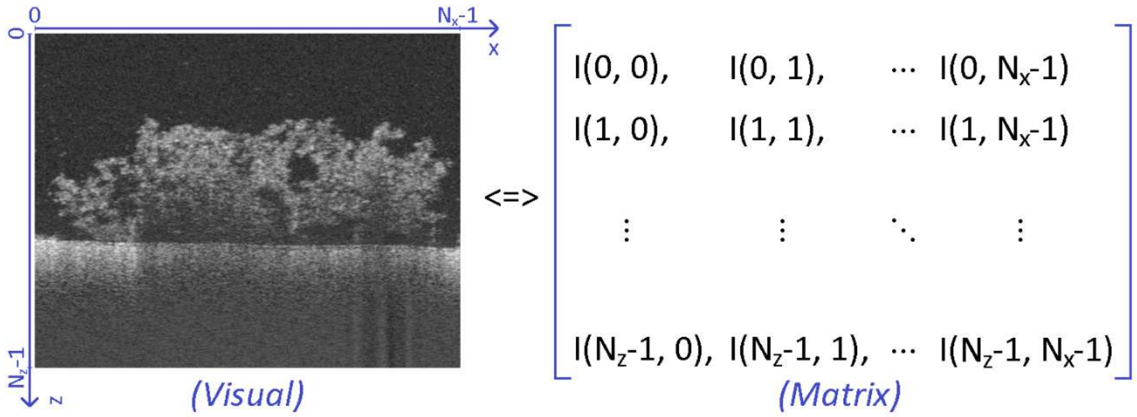

Figure 5: Example of 2D OCT biofilm image (left). These images are imported and processed in BISCAP as equivalent 2D matrices (right), where all matrix entries include the corresponding pixel grayscale intensities.

The total number of pixels in the vertical/horizontal directions in 2D OCT images are denoted as N_z and N_x, respectively. 3D images also include a total of N_y positions along the depth axis. Consistently with the standard convention in the field, the vertical axis (z) increments from the top ($z = 0$) to the bottom ($z = N_z - 1$), the horizontal axis (x) increments from the left ($x = 0$) to the right ($x = N_x - 1$), and the depth axis from the front ($y = 0$) to the back ($y = N_y - 1$).

BISCAP includes three image-processing functionalities:

- Pre-processing (Section 4)
- Automatic processing (Section 5)
- Post-processing (Section 6)

Three distinct modes/workflows for full image processing are built into BISCAP, making use of these three basic functionalities in a slightly different way, as follows:

- Single image processing 2D (Section 7)
- Multi-images processing 2D (Section 8)
- Single image processing 3D (Section 9)

On launching BISCAP, the **Home** screen is loaded, as illustrated in Figure 6, from where one of the three modes above may be selected. However, since all modes share the three steps above with only slight variations between them, these functionalities are presented first (Sections 4–6), and then their utilization in the context of the three modes (Sections 7–9) is discussed. Quick-start tutorials for the three modes are then presented in Sections 10–12.

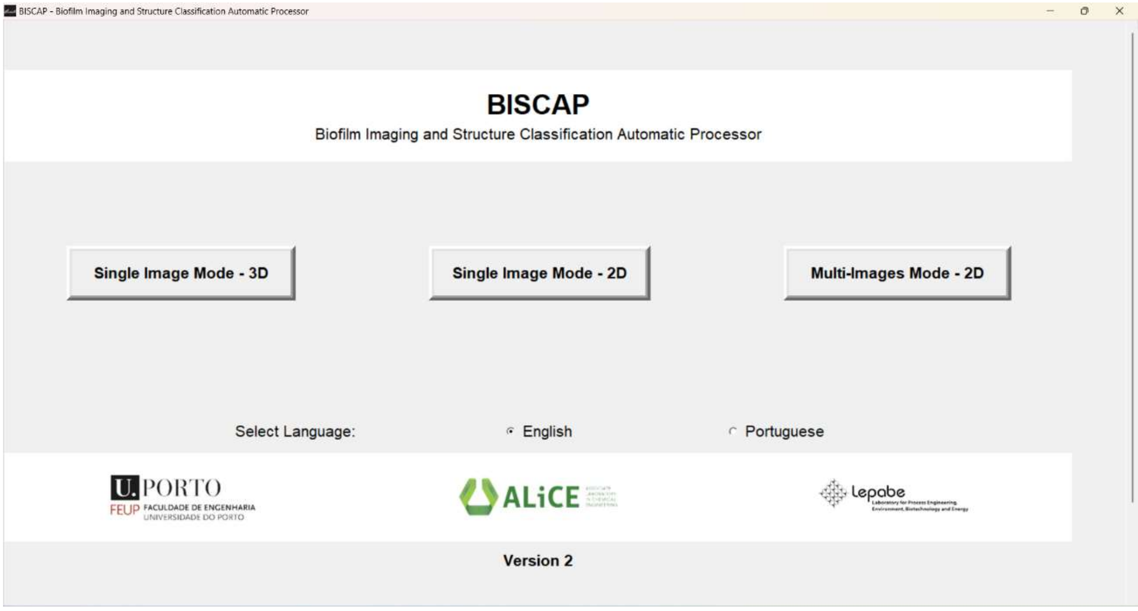

Figure 6: **Home** screen in BISCAP. All image processing modes are accessible from this screen.

It is assumed that all biofilm image files are saved to folders, including exclusively tiff or png files obtained directly from 2D OCT scans or exclusively tiff files for the case of 3D scans. Upon conclusion of image processing and taking the root folder for any given image as the reference, a set of additional subfolders are created, as follows:

- "inputs": includes all auxiliary files saved during pre-processing.
- "outputs": includes all critical results from the automatic processing and post-processing stages (as listed in the main manuscript).
- "post_proc": matrices from automatic processing saved in equivalent image files; this information is necessary to process regions of interest and obtain the corresponding structural parameters in the post-processing stage.
- "temp": while an image is under processing in BISCAP, temporary auxiliary files are saved in this folder. The folder is deleted after processing is completed.

This folder structure must be kept as described above to ensure BISCAP delivers the desired functionality. To facilitate image processing in BISCAP, images are resized to fit on the screen, which may sometimes not preserve the original height/length ratio. Note that all images delivered by BISCAP keep this ratio and are also available for additional checking via the standard image display applications from these folders.

Note: Image files may be saved with any names as required, and generally it is recommended to keep them as short and succinct as possible. The same recommendation holds for paths. In some systems, paths including spaces (e.g. "…\my folder\...") on the folders/paths including the images of interest, may prevent the application from loading images correctly.

4. Pre-processing

Pre-processing is an optional functionality in BISCAP, whereby (raw) 2D or 3D OCT images are conveniently trimmed along the vertical axis to include only a central horizontal band, including all biofilm pixels. This is achieved via manual user input, and a good understanding of what constitutes biofilm and substratum pixels (in line with the Definitions) is required. While this is an optional step in BISCAP, it is also highly recommended since the minimal effort required in pre-processing generally enables significant improvements in accuracy and computational efficiency.

Three **Pre-processing** screens were developed: one for the single 2D image mode, one for the 2D multi-images mode, and another for the single 3D image mode. While there are minor differences between them to accommodate the distinct workflows in the three modes, their basic functionality is the same and presented in sequence. Additional details on loading images and specific command buttons are discussed separately in Sections 7–9.

Once a raw biofilm image is selected, it is displayed accordingly in the **Pre-processing** screen of BISCAP. This screen includes several buttons, as depicted in Figure 7.

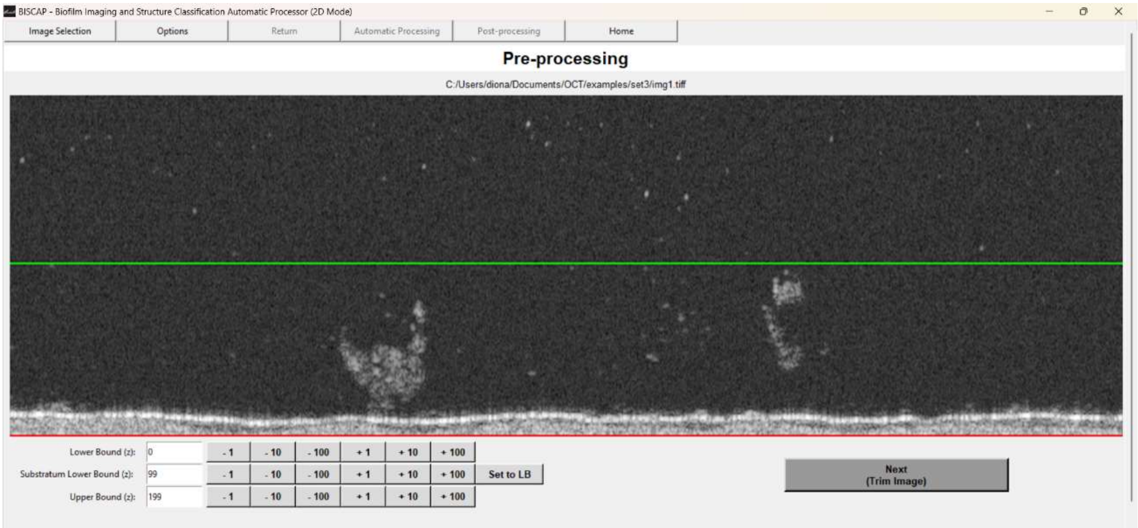

Figure 7: **Pre-processing** screen in BISCAP (single image mode – 2D): raw image with default bounds.

Three bounds on the vertical axis are defined and visible on the lower left portion of the **Pre-processing** screen and shown over the raw image. These bounds are defined as follows:

- *Lower Bound (z):* The lower bound of the horizontal band of interest; all biofilm pixels/voxels must be below this bound.
- *Substratum Lower Bound (z):* Auxiliary bound for substratum identification; all substratum pixels/voxels must be below this bound.
- *Upper Bound (z):* The upper bound of the horizontal band of interest; all biofilm pixels/voxels must be above this bound.

By default, the *Lower Bound (z)* is set to $z = 0$, and the *Upper Bound (z)* is set to $z = N_z - 1$. These bounds control the width of the horizontal band of interest and are initialized to include the full vertical range of raw images. Using the definitions above and the negative

and positive increment buttons (-1, -10, -100, +1, +10, +100), all bounds should be set as tightly as possible. Increments of 10 and 100 are usually sufficient for this purpose.

Pre-processing allows additionally to obtain auxiliary information for the automatic processing stage. Specifically, the *Substratum Lower Bound (z)* was defined to minimize the impact of very bright pixels above the substratum and enhance the automatic identification of the bottom interface of biofilms. Unlike the lower and upper bounds, this bound does not contribute to trimming raw images and is used during automatic processing. It is initialized by default at $z = (N_z - 1)/2$, and should also be set as tightly as possible for an accurate identification of the bottom interface. Figure 8 illustrates the fine selection of these bounds.

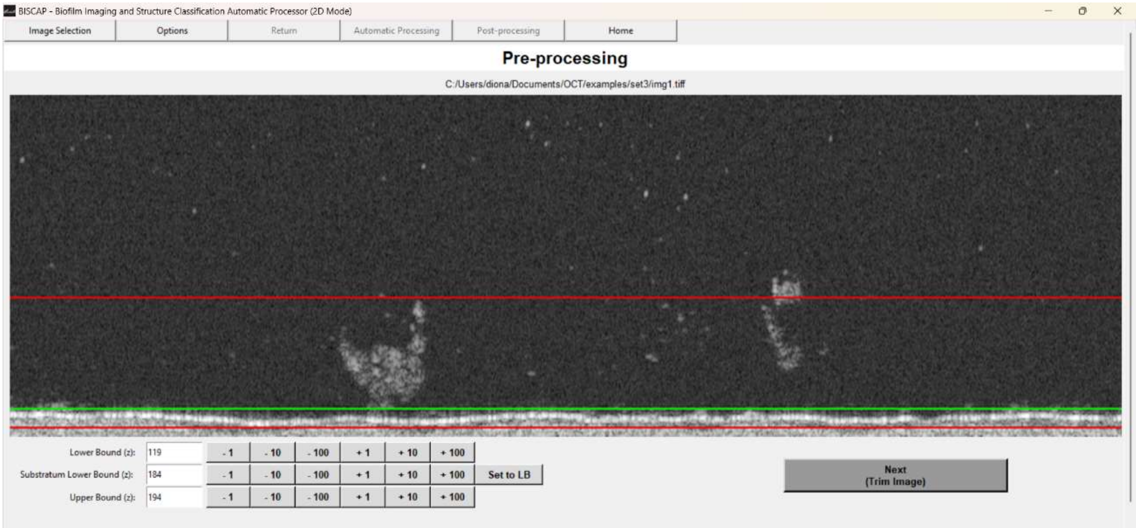

Figure 8: **Pre-processing** screen in BISCAP (single image mode – 2D): raw image with user-selected bounds.

Observe that *Lower Bound (z) ≤ Substratum Lower Bound (z) < Upper Bound (z)*. A warning is displayed if a change is made on these bounds via the increment buttons violating this rule, and the action is cancelled. In the extreme case of strongly inclined substratum interfaces, the *Lower Bound (z)* may equal the *Substratum Lower Bound (z)*. In this case, the *Lower Bound (z)* should be firstly set, and then using the Set to UB button, the *Substratum Lower Bound (z)* is conveniently made to coincide with the former.

Bounds' colours and thicknesses, as displayed on the screen, may be adjusted via the Options button for convenience. The Save button must be clicked to activate any changes made on this window, which will also close the options window. These options are depicted in Figure 9.

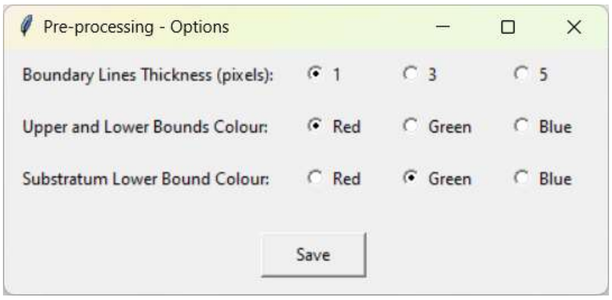

Figure 9: Pre-processing options.

The description presented so far concludes the interactive part of pre-processing. To finalize pre-processing, an additional button (details for the three modes/workflows in Section 7–9) takes the specified user-defined bounds and automatically trims raw images accordingly. These images are conveniently displayed in BISCAP for assessment (Figure 10). Pre-processed images are also saved to the "inputs" subfolder. Pre-processed images are named the same as the corresponding raw image with the appended suffix "_input". All bounds are saved to an Excel file ("inputs.xlsx") and included in the "inputs" subfolder. Note that if this information is saved for a given image and the **Pre-processing** screen is resumed later, all bounds will be shown accordingly and not using the defaults presented earlier.

As a final note, the lower bound for image trimming is not defined exclusively via the *Lower Bound (z)*; an additional gap (in pixels) is used for trimming raw images. This extra gap is necessary for the automatic thresholding module. This is illustrated via Figures 8 and 10: note that the band of pixels defined (Figure 8) does not match the obtained pre-processed image (Figure 10); instead, the pre-processed image extends above the defined bound.

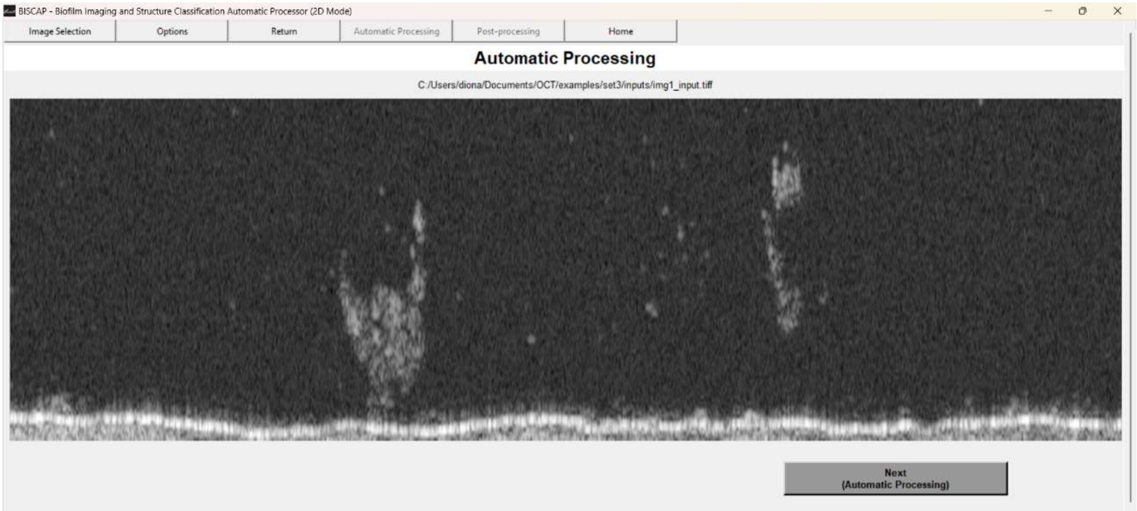

Figure 10: Pre-processed image after bound selection and (raw) image trimming (displays in **Automatic Processing** screen if the single image mode is selected).

5. Automatic Processing

Automatic processing comprises a set of automatic calculations in four stages:

- Bottom interface
- Binarization
- Biofilm structure
- Top interface

Detailed descriptions of these stages are available in the main manuscript and Appendix 1. This manual presents automatic processing exclusively from a user perspective, thus not covering their underlying principles.

Three **Automatic Processing** screens were developed: one for the single 2D image mode, one for the 2D multi-images mode, and another for the single 3D image mode. Their core functionality is the same, and specific details for their utilization in the context of these modes are presented in Sections 7–9. It is assumed at this stage that pre-processing was concluded, and pre-processed image(s) have been obtained as described in Section 4. Figure 10 depicts the **Automatic Processing** screen.

This task is significantly less interactive than pre-processing, and no user input is strictly required by default. Automatic processing may begin immediately once pre-processing is concluded. However, several optional settings in this mode may be adjusted. The parameter specification window is available from the Options button, as shown in Figure 11. All groups of available settings are presented in sequence.

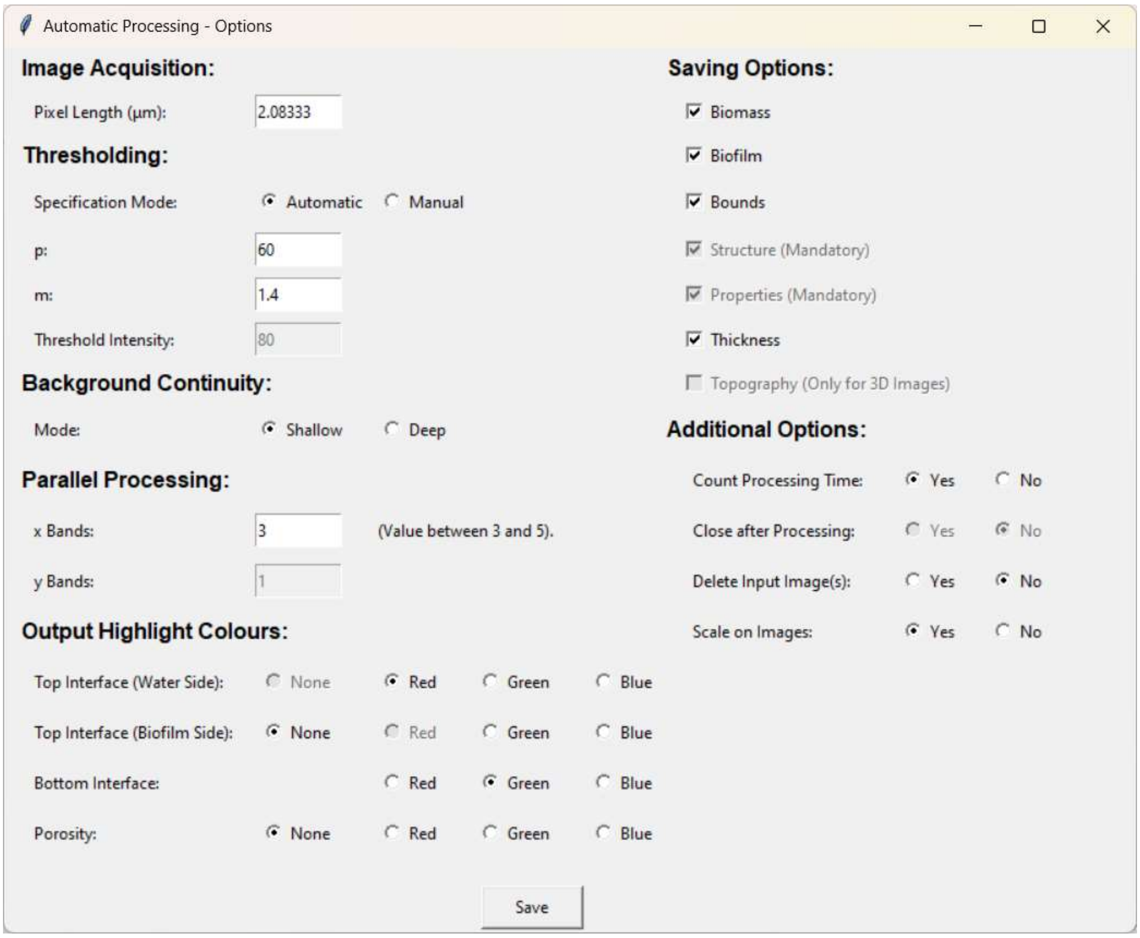

Figure 11: Optional automatic processing settings (single image mode – 2D).

Image acquisition:

A default is given for *Pixel length (μm)*, such that the length of 100 consecutive pixels/voxels along the vertical direction of OCT images equals 48μm. Users must set this parameter according to the local image acquisition set-up and update it whenever necessary.

Thresholding:

This is likely the most important set of processing options, with a noticeable impact on biofilm structure. Automatic and manual threshold *Specification modes* are available. In the first case, parameters p $(0 \leq p \leq 100)$ and m $(m > 1)$ may be customised. Additional details on these parameters may be obtained from Narciso et al. 2022. These have been empirically tuned via a set of 300 biofilm images with highly satisfactory results obtained. Updating these parameters should originate from a rigorous critical analysis of results. Generally, the higher are p and m, the higher are the corresponding threshold intensities. Alternatively, users may select the manual specification mode and set a constant *Threshold intensity* in the corresponding box.

Background continuity:

Two modes are now available to detect all pixels/voxels in the top region. The *shallow*/*deep modes* do not/do allow continuity from pixels at the top interface. As a result, the calculated positions of the top interface in the first mode capture the outer contours of biofilm structures. The top interface generally penetrates deeper into the biofilm structure in the second mode. In turn, this selection typically has a noticeable impact on porosity calculation.

Parallel Processing:

All continuity calculations are based on a parallel processing architecture. This generally enables faster computations, except in small images where the overheads associated with creating additional processes may be unfavourable. Users must specify how many partitions are defined along the horizontal axis (*x Bands*) – 2D and 3D modes – and the depth axis (*y Bands*) – 3D mode only. The total number of small chunks created is thus *x Bands* * *y Bands*; if both parameters are set to 1, pixel/voxel classification is executed in the full matrix using a single process.

Powerful desktop machines can make use of many parallel processes, whereas, in laptop machines, this is typically a smaller number. This consideration is essential to determine the maximum values for these parameters. The optimal setting of *x Bands* and *y Bands* has yet to be studied: on the one hand, it is desirable to distribute all tasks by as many processes as possible, but on the other hand, this requires additional operations along their edges. Partitioning 2D images in <u>3</u> small chunks and 3D images in <u>5*5</u> chunks is offered as a <u>rough guideline</u> at this stage. Note that if the number of bands is over (roughly) 1/3 of the total number of positions in the corresponding axes, this may yield empty chunks during parallel processing, which causes a fatal error. A much smaller fraction is generally advised.

Saving options:

The primary purpose of automatic processing is to deliver a set of useful images and numeric outputs for biofilm analysis. These outputs are presented in Table 1:

Table 1: Outputs available from automatic processing.

Output	Description	Suffix
Biomass	Pixels/voxels above the bottom interface with grayscale intensities above/below a threshold are displayed in white/black.	"_biom"
Biofilm	Biomass pixels/voxels defining a continuous structure from the bottom interface are displayed in white. All others are displayed in black.	"_biof"
Bounds	Taking the pre-processed image, all pixels/voxels at the bottom and top interface are given a high contrast colour to emphasize their contours (output 1 in BISCAP v1).	"_bnds"
Structure	All pixels/voxels in the biofilm region retain their original grayscale colours (from pre-processed images). The remaining pixels/voxels from the bottom and top regions are displayed in black to emphasize the biofilm structure (output 2 in BISCAP v1).	"_struct"
Properties	Structural (numeric) parameters of biofilm, saved as an Excel file (output 3 in BISCAP v1).	"_props"
Thickness	Thickness in all x positions of 2D images is shown as a series; thickness in all (y, x) positions of 3D images is shown as a heatmap (output 4 in BISCAP v1).	"_thick"
Topography	Applicable only for 3D images: all voxels at the top interface are represented as a 3D topography map.	"_topogr"

The majority of outputs may be enabled/disabled via the corresponding checkboxes. Only the selected outputs are calculated and then saved to the "outputs" folder. All outputs are saved with the same name as the corresponding raw image, where the suffixes listed in Table 1 are used to distinguish between them.

When processing is concluded, two additional files are also (mandatorily) saved per image in the "post_proc" folder: (i) "biof_mat", and (ii) "struct_mat". These files preserve the information from automatic processing, namely concerning: (i) biofilm vs. rest classification and (ii) biofilm region vs. rest classification, respectively. This information is necessary for post-processing to calculate the structural parameters of any defined region of interest.

Output highlight colours:

These options control the display of the third output listed in Table 1 (Bounds). Consistently with the outputs from automatic processing, the set of pixels/voxels at the top interface includes, in fact, two sub-groups: (i) those which are included in the top region and are neighbours to at least one pixel/voxel in the biofilm region (*Top Interface (water side)*), and (ii) those which are included in the biofilm region and are neighbour to at least one pixel/voxel in the top region (*Top Interface (biofilm side)*). In BISCAP v1,

only the first of these options was considered. Both interfaces may now be displayed; in this case, users must specify different colours to distinguish between them. To disable printing them, the radio button must be set to None. Note that at least one of these interfaces must be specified a colour to display biofilm contours.

Users may also select a colour for the *Bottom interface* in this section. A new option was enabled in this version of BISCAP where all porosity pixels/voxels classified as porosity during the automatic processing may be highlighted if required. For this purpose, the parameter *Porosity* controls this display. If set to None, this class of pixels/voxels retains their original grayscale colours in the Structure image output (same as in BISCAP v1); if one the three colours available is selected, these pixels/voxels are marked accordingly in the Structure image output. Users are advised that using this option in shallow mode can result in some pixels/voxels being classified in this category, even though they are not isolated within the biofilm structure. This is the result of the continuity testing architecture put in place for the shallow mode, which limits the extent of this task.

Additional options:

Users may time the duration of automatic processing (*Count processing time*) if required. An option is also given to close the application on finishing after automatic processing concludes (*Close after processing*). This may be useful when large amounts of images are to be processed and available only in the 2D multi-images mode. To minimise the amount of data saved, and once all automatic processing tasks are completed on a given image, the corresponding input file is not strictly needed and may be deleted (*Delete input image(s)*). There is also an option to print a scale in the top left corner of all image outputs (*Scale on image(s)*). This option is available only in the 2D image processing modes.

Details on the launching and progress of automatic processing are discussed in Sections 7–9 for the three available modes. Once launched and until processing is concluded, a message is displayed accordingly in BISCAP, and all buttons remain inaccessible. During this stage, BISCAP may appear to freeze, but this is not the case. Total processing time depends strongly on the size and complexity of biofilm images and computational power. On a standard desktop machine, 2D image processing takes a few seconds (<5s). In 3D, total processing times are in the order of minutes (5-10min).

6. Post-processing

This function allows the convenient visualization of all outputs obtained during automatic processing and customized biofilm analysis in user-defined regions of interest along the horizontal/depth axes. Three **Post-processing** screens were developed: one for the single 2D image mode, one for the 2D multi-images mode, and another for the single 3D image mode. Their core functionality is very similar, particularly concerning the 2D modes. There are noticeable differences concerning the 3D mode, and the two subjects are presented in Sections 6.1 and 6.2, respectively. The utilization of post-processing in the context of these three modes is presented in Sections 7–9.

6.1 2D Post-processing

The **Post-processing** screen for the 2D modes is depicted in Figure 12. It is assumed that at this stage, automatic processing is finalized.

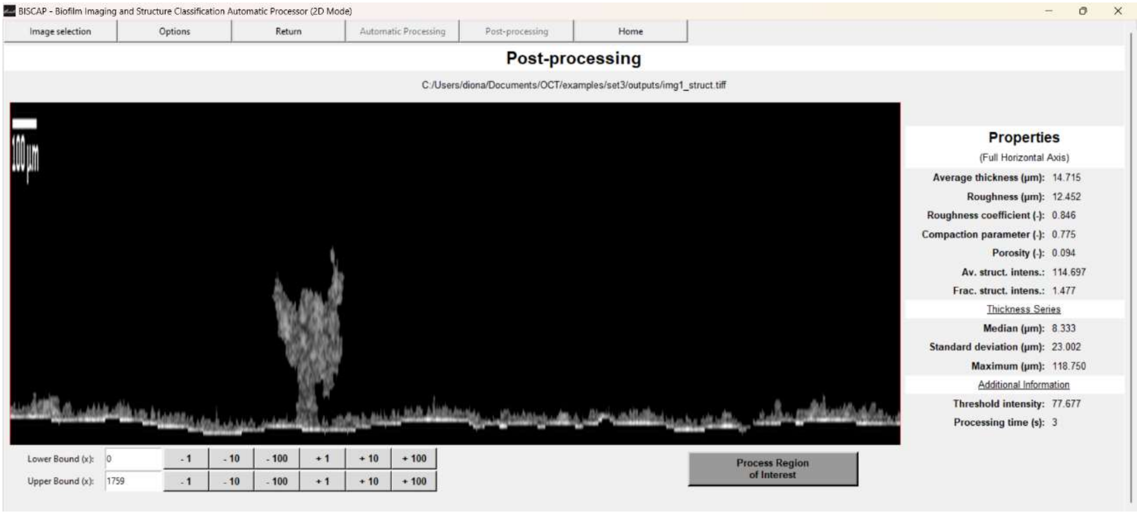

Figure 12: **Post-processing** screen (single image mode – 2D).

Biofilm structural properties are displayed on the right side of the screen. Note that these results are calculated for the full length of the horizontal/depth axes. The key biofilm structural parameters are: (i) average thickness, (ii) roughness, (iii) roughness coefficient, (iv) compaction parameter, and (v) porosity. Two additional parameters are also calculated: (vi) average (biofilm) structure intensity and (vii) its ratio with the calculated threshold intensity. Statistics concerning the thickness series (maximum, standard deviation, and median), the threshold intensity, and the total processing time are also displayed.

A single image fills the larger portion of the **Post-processing** screen to the left of the list of properties. By default, biofilm structure is displayed. From the Options button, all remaining outputs obtained from automatic processing may be selected by enabling the corresponding option from the parameter *Displayed output*. The options window for the **Post-processing** screen is shown in Figure 13.

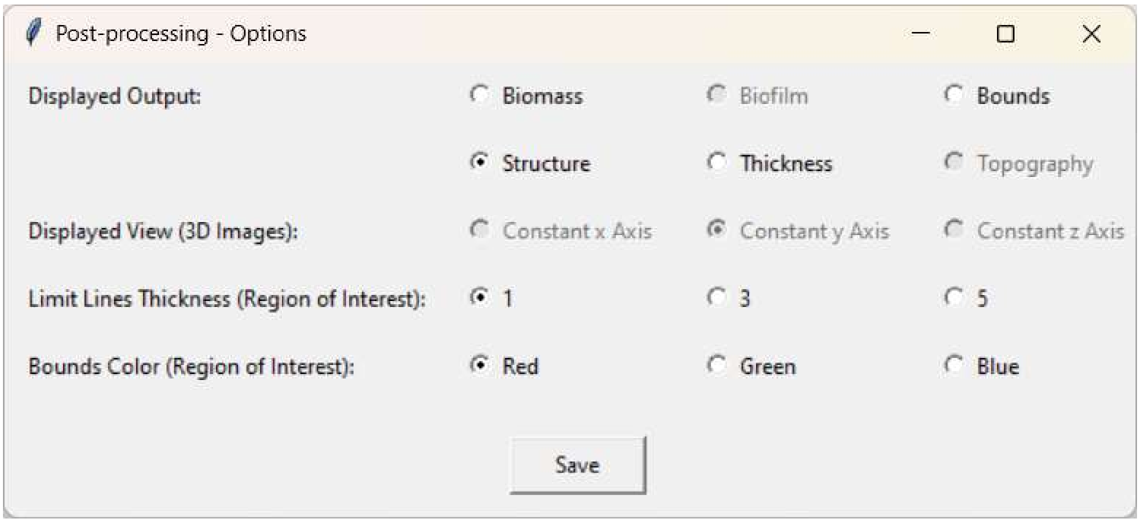

Figure 13: Post-processing options (single image mode – 2D).

The option *Displayed view* is not applicable in the 2D modes and is covered in Section 6.2. Note that users can only select those outputs marked for calculation in the automatic processing options (Figure 11). Thickness and the colours of a set of auxiliary boundary lines are also available from the options window. These vertical lines enable the definition of a region of interest and are shown below the displayed output in the **Post-processing** screen:

- *Lower Bound (x)*: Defines the left vertical edge of the defined region of interest.
- *Upper Bound (x)*: Defines the right vertical edge of the defined region of interest.

By default, *Lower Bound (x)* is set to $x = 0$ and *Upper Bound (x)* is set to $x = N_x - 1$. In this case the region of interest coincides with the full horizontal range. Using the increment buttons to the left of these parameters (-1, -10, -100, +1, +10, +100), any region of interest may then be selected, provided that *Lower Bound (x)* < *Upper Bound (x)*. Note that the defined region of interest includes the lines in the image.

Once a region of interest is defined, the Process Region of Interest button calculates all biofilm structural parameters for the present selection and saves the corresponding image. A window is displayed, including these results, as illustrated in Figure 14. These additional user-defined outputs are also saved in the corresponding "outputs" subfolder. They are saved with the same name as the original outputs for the full horizontal axis and with an appended suffix also identifying the selected bounds (e.g. "_props_xmin_xmax").

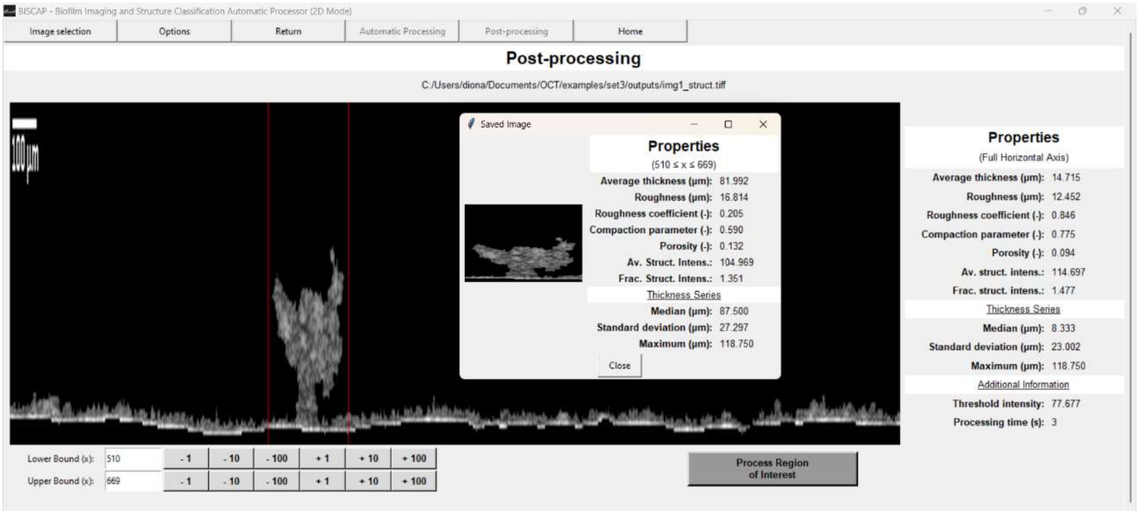

Figure 14: **Post-processing** screen (single image mode – 2D): region of interest and detailed biofilm analysis.

6.2 3D Post-processing

By default, the **Post-processing** screen in the 3D mode displays the thickness heatmap since this is the most convenient output to define regions of interest in the horizontal and depth axis (entirely consistent with its geometry). Biofilm structural properties are also shown on the right (same as in 2D). This screen is shown below:

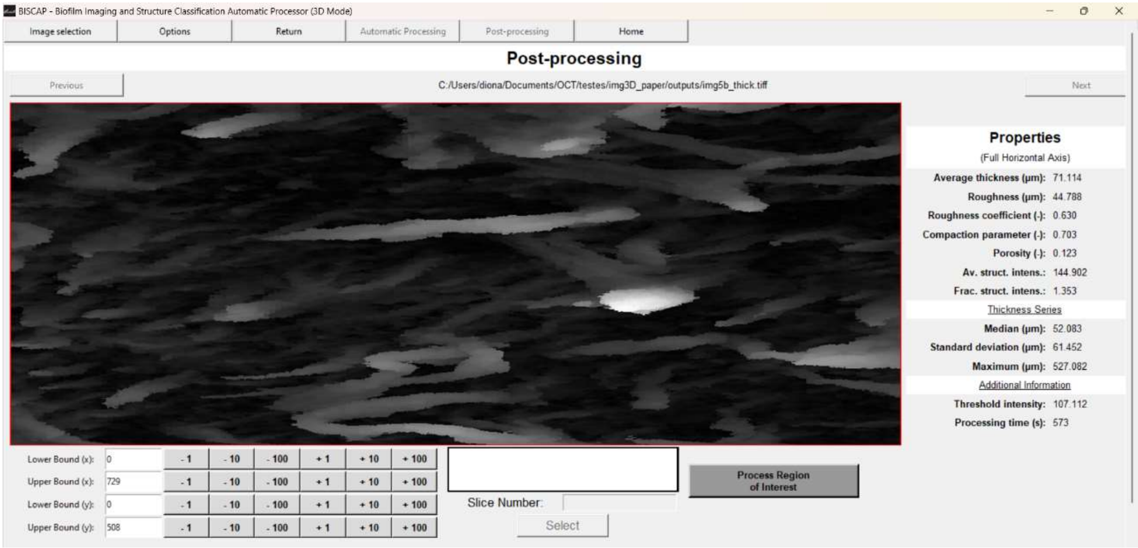

Figure 15: **Post-processing** screen (single image mode – 3D): thickness heatmap.

Additionally to the buttons enabling the definition of the region of interest along the horizontal axis (Section 6.1), a new set of buttons is also created for the depth axis:

- *Lower Bound (y)*: Defines the bottom horizontal edge of the defined region of interest.
- *Upper Bound (y)*: Defines the top horizontal edge of the defined region of interest.

As illustrated below, any region of interest may be defined using these buttons. To obtain the properties, press the button Process Region of Interest. The properties are displayed

on the screen and saved to the "outputs" folder. In this case, no additional image output is saved. Note that the defined regions of interest also include the four bounds.

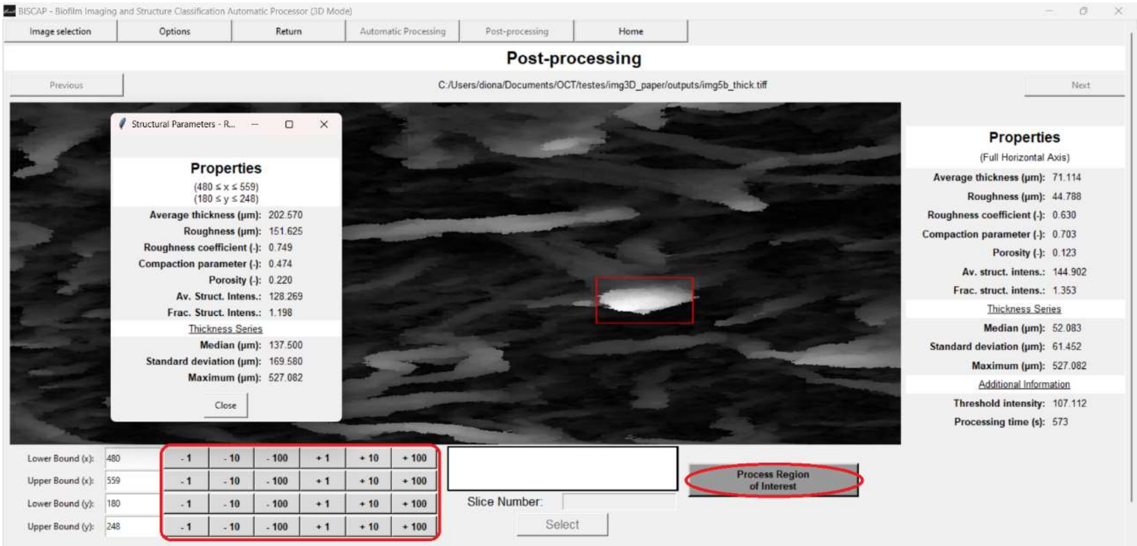

Figure 16: **Post-processing** screen (single image mode – 3D): properties of regions of interest.

The Options button may also enable the display of the remaining image outputs. The first four image outputs in Table 1 have the same data structure, and their visualization follows the same principles. These are 3D images, and all their 2D slices may be visualized. Note that this is not restricted to slices at constant y only as used in pre-processing, but any of the three axes may be selected and their slices inspected. To do this, from the displayed view in the Options button/window (Figure 13), select an axis via *Displayed View*, and then using the Previous and Next buttons, browse through all their slices. Alternatively, insert the *Slice Number* and press the Select button to jump directly to any slice of interest.

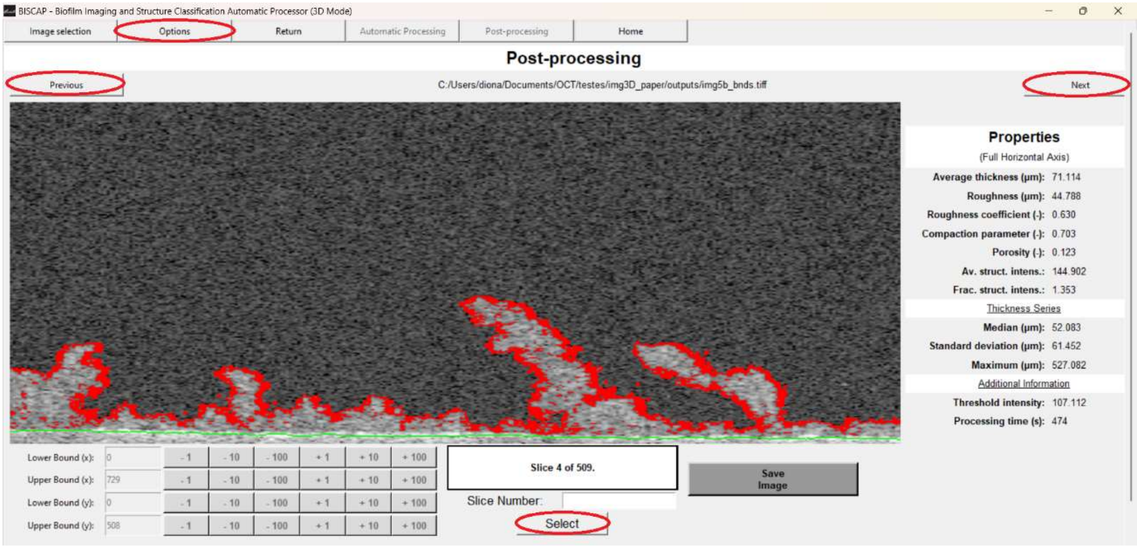

Figure 17: **Post-processing** screen (single image mode – 3D): biofilm contours at a constant y slice.

The current display on the screen may be saved as an image in the “outputs” folder via the Save Image button. Images are saved with the same name as the corresponding output with an additional suffix (e.g. “_bnds_y_4”).

The topography of the top interface can also be inspected from the Options button/window. This offers a 3D insight into biofilm shape, where those regions with higher/lower thicknesses are shown in red/blue.

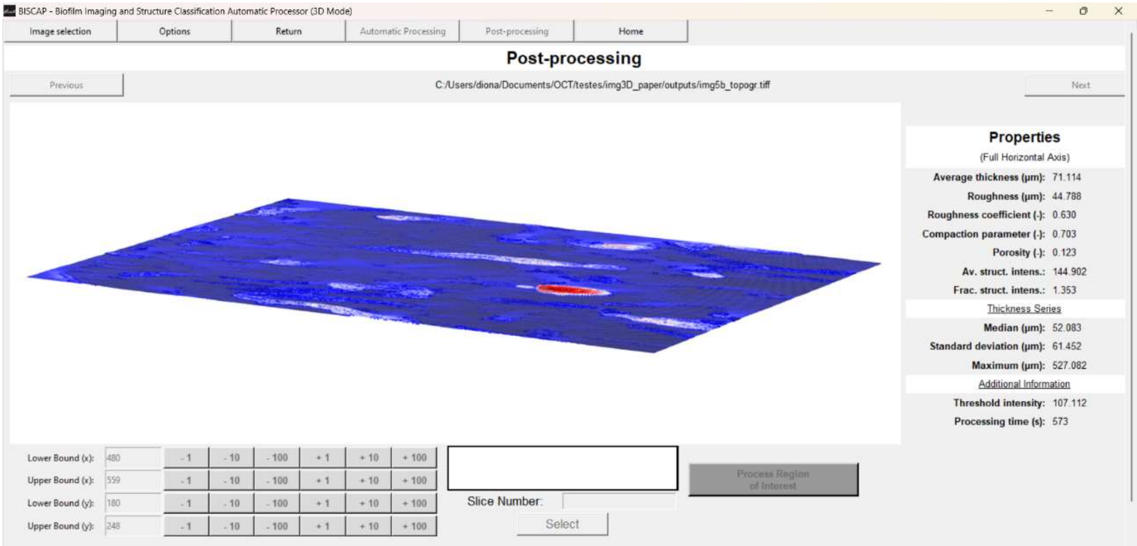

Figure 18: **Post-processing** screen (single image mode – 3D): the topography of the top interface.

7. Single Image Mode 2D

This mode is accessible via the (initial) **Home** screen. As the name suggests, it is tailored specifically for processing a single biofilm 2D image. The three functionalities as described in Sections 4–6 are thus displayed sequentially in BISCAP via three screens: **Pre-processing → Automatic Processing → Post-processing**, where each of these screens only becomes available if the necessary actions are completed (e.g. **Automatic Processing** screen is available only if pre-processing is concluded).

Pressing the Single Image Mode – 2D button from the **Home** screen displays the **Pre-processing** screen. A set of navigation buttons are permanently displayed in this mode, enabling users to move through the three available processing functions (subject to the conclusion of all precursor steps). A description of these buttons is presented below:

- Image selection: Clears the selected file from memory (if any) and opens a window to browse to a 2D OCT image of interest.
- Return: Displays the previous screen according to the natural processing sequence defined above (not actionable in the **Pre-processing** screen – first step).
- Automatic Processing: Displays the **Automatic Processing** screen without re-running pre-processing. Only actionable if the corresponding pre-processed image is available.
- Post-processing: Displays **Post-processing** screen without re-running pre-processing and automatic processing. Only actionable if outputs from automatic processing are available.
- Home: Clears the selected file from memory (if any) and displays the **Home** screen.

Image processing begins with the selection of a 2D OCT image. The buttons above enable navigation between the three screens to some extent, subject to the availability of relevant results. The buttons effectively executing the main actions as described in Sections 3 and 4 are available in the corresponding screens:

- Next (Trim Image) (**Pre-processing** screen): Takes the defined user-selected bounds and trims the selected raw image. Saves the corresponding pre-processed image and bounds' values and moves the display to the **Automatic Processing** screen.
- Next (Automatic Processing) (**Automatic Processing** screen): Launches automatic processing of the pre-processed image using the select processing options. The **Post-processing** screen is displayed, but no actions are available until processing is completed.

The standard workflow for this mode is as follows:

1) Press the Single Image Mode – 2D button from the **Home** screen.
2) Select a 2D OCT image via the Image selection button.
3) **Pre-processing:** Adjust all bounds as described in Section 4, and once completed, press the Next (Trim Image) button.
4) **Automatic processing:** Adjust any relevant options and press the Next (Automatic Processing) button.
5) **Post-processing:** Select outputs to visualize from the Options button, and define and analyse any region of interest if required (Section 6.1).
6) Use the navigation buttons above if necessary to move between screens.

Note: all input and output Excel and image files may be opened and checked independently of tasks executed in BISCAP. However, users are advised to close these Excel files if the main pre-processing or automatic processing tasks are launched. If not, BISCAP is unable to write the Excel files, and the application freezes.

8. Multi-images Mode 2D

From a user perspective, the single image mode would be cumbersome and inefficient for processing multiple 2D images. This is a consequence of the unavoidable waiting times between processing any two images until automatic processing completes. An alternative workflow was developed for this case to circumvent this limitation.

Rather than focusing on a set of images and executing the three processing functions for all of them as described in Section 7, in the multi-images mode, the focus is put on the three processing functionalities individually and obtaining the necessary results for all selected images. The workflow is now as follows:

1) Pre-process all selected images.
2) Execute automatic processing for all selected images.
3) Post-process all selected images (if required).

Pre-processing is the only task requiring significant user input. Once completed, during the automatic processing stage, all images are processed sequentially without the need for user input and supervision, thus becoming a more practical approach for image processing. In the end, and if required, users may also post-process results as described earlier.

In the **Home** screen, the **Multi-Images Mode** screen is accessible from the corresponding button (Multi-Images Mode – 2D). This screen displays four buttons, enabling the navigation to the three available image processing screens (**Pre-processing**, **Automatic Processing,** and **Pre-processing**) or back to the **Home** screen.

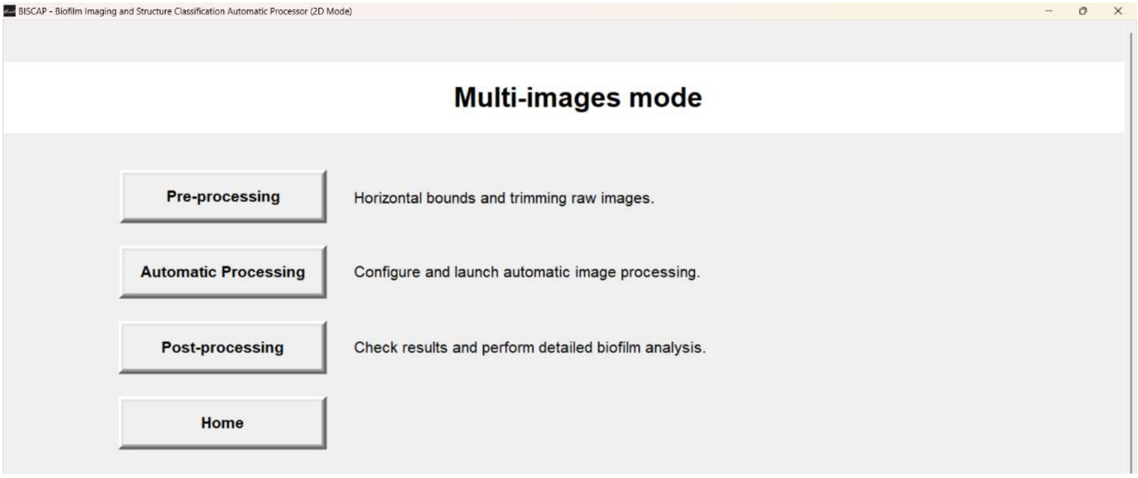

Figure 19: Main navigation screen in multi-images 2D.

All buttons are actionable at all times. However, the same principles of results availability as described in Section 7 apply in this mode. The utilization of these image processing functions in the context of the multi-images mode is presented in sequence.

8.1 Pre-processing

The **Pre-processing** screen is depicted in Figure 20:

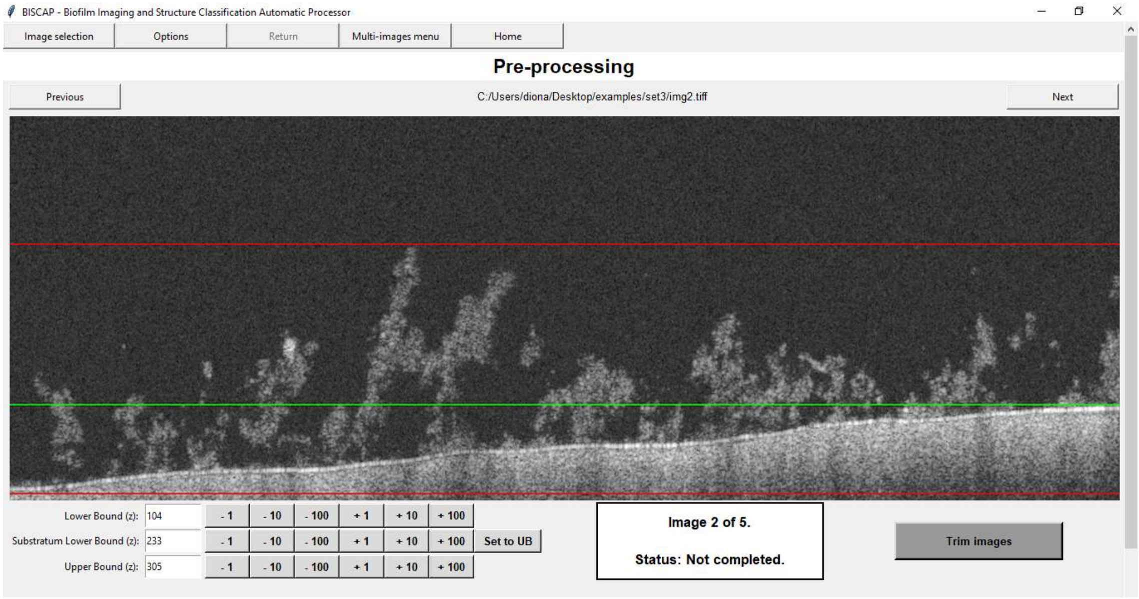

Figure 20: **Pre-processing** screen (multi-images mode – 2D).

Additionally, to the main functionality presented in Section 4, the **Pre-processing** screen also includes four navigation buttons, as follows:

- Image selection: Clears the selected files from memory (if any) and opens a window to browse to a folder of interest.
- Return: After executing pre-processing, trimmed images are displayed, and increment buttons are not actionable. At this stage, the Return button becomes actionable, and if pressed, BISCAP resumes the selection of horizontal bounds.
- Multi-images menu: Clears the selected files from memory (if any) and displays the main **Multi-images mode** screen.
- Home: Clears the selected files from memory (if any) and displays the **Home** screen.

Rather than selecting a single file, a folder must be specified in this mode. All images within the selected folder are loaded to BISCAP and available for pre-processing. A single image is displayed at the time, and using the Next and Previous buttons, users may visualise all images in the selected folder. Then, all bounds must be adequately adjusted using the increment buttons, as discussed in Section 4. Once completed, the Trim images button delivers and saves the pre-processed images from raw images, as mentioned in Section 4.

8.2 Automatic Processing

The **Automatic Processing** screen is depicted in Figure 21:

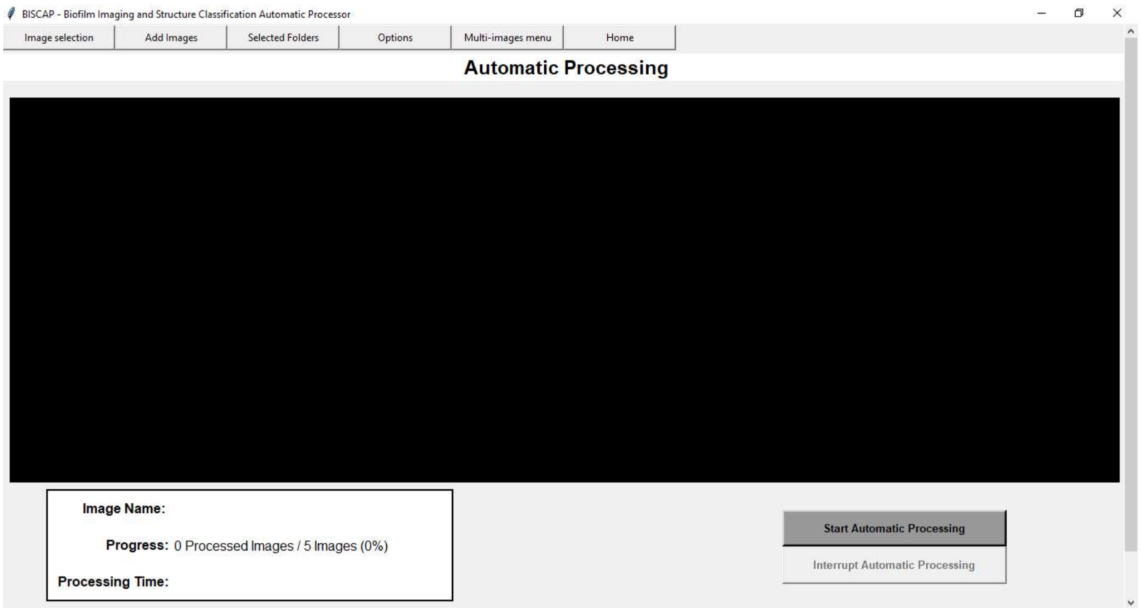

Figure 21: **Automatic Processing** screen (multi-images mode – 2D).

Additionally to the navigation buttons already discussed in Section 8.1, two extra buttons are available in the **Automatic Processing** screen, as follows:

- Add Images: Any previously selected file(s) are kept, and a window is displayed to specify an additional folder and load biofilm images therein.
- Selected Folders: Displays all currently selected folders for automatic processing.

Any number of folders is allowed. The only requirement is that pre-processing was completed for all images in the selected folders. If this is not the case, a warning is displayed accordingly. Next, BISCAP sequentially processes all images and saves all results to disk. To achieve this, it suffices to adequately set the processing options presented in Section 5 and then launch automatic processing via the Start Automatic Processing button. The Interrupt Automatic Processing button may be used during processing to cancel this action. In this case, only a fraction of all results requested initially is saved to disk.

During automatic processing, the current image being processed is displayed on the screen, and its name is shown below. Progress towards completion is also shown in the lower left corner, and if enabled, the total processing time is also displayed when the activity completes. On completion, a summary Excel file ("outputs.xlsx") is saved for all selected directories in the corresponding "outputs" subfolders, additionally to the Excel files saved individually for all images processed. This provides convenient access to all results and facilitates their comparison.

8.3 Post-processing

All navigation buttons presented in Section 8.1 are available on the **Post-processing** screen. Users must select a folder for which results from automatic processing have been obtained at an earlier stage using the Image selection button. A warning message is displayed if a chosen folder does not satisfy this requirement. All results for the selected folder are loaded to BISCAP as a gallery of images. As described earlier, all results may

be visualized using the Previous, Next, and Options buttons. Additionally, regions of interest may be defined for all outputs as described in Section 6.1.

8.4 Workflow

To conclude, the standard workflow for this mode is as follows:

1) Press the Multi-images mode – 2D button from the **Home** screen.
2) **Pre-processing:** Press the Pre-processing button from the **Multi-images mode** screen. Select a folder, and adjust all bounds of biofilm images as described in Section 8.1. Once completed, press the Multi-images menu button.
3) **Automatic processing:** Press the Automatic Processing button from the **Multi-images mode** screen. Select the same folder as in step 2, and launch automatic processing as described in Section 8.2. Once completed, press the Multi-images menu button.
4) **Post-processing:** Press the Post-processing button from the **Multi-images mode** screen. Select the same folder as in step 2, and browse through the images gallery to check results and define any regions of interest as described in Section 8.3. Once completed, press the Home button.

9. Single Image Mode 3D

This mode is accessible from the **Home** screen. As the name suggests, it is tailored specifically for processing a single biofilm 3D image. The three image processing functions, as described in Sections 4–6, are thus displayed sequentially in BISCAP via three screens: **Pre-processing → Automatic Processing → Post-processing**, where each of these screens only becomes available if the necessary actions are completed.

Pressing the Single Image Mode – 3D button from the **Home** screen displays the **Pre-processing** screen. A set of navigation buttons is permanently displayed while in this mode, enabling users to move through the three available processing functions (subject to the conclusion of all precursor steps). These buttons are the same as presented in Section 7: (i) Image selection, (ii) Return, (iii) Automatic Processing, (iv) Post-processing, and (v) Home.

Image processing begins with the selection of a 3D OCT image. The buttons above enable navigation between the three screens to some extent, subject to the availability of relevant results. The buttons effectively executing the main actions as described in Sections 4 and 5 are available in the corresponding screens, as explained in Section 7: (i) Next (Trim Image) and (ii) Next (Automatic Processing).

The standard workflow for this mode is as follows:

1) Press the Single Image Mode – 3D button from the **Home** screen.
2) Select a 3D OCT image, via the Image selection button.
3) **Pre-processing:** Adjust all bounds as described in Section 4, and once completed, press the Next (Trim Image) button. In the 3D case, bounds must be set adequately by taking into account all 2D image slices along the depth axis. Using the Next and Previous buttons, users may visualise all slices. Any slice of interest may also be selected using the parameter *Slice Number* and the Select button.
4) **Automatic processing:** Adjust any parameters from the Options button/window and press the Next (Automatic Processing) button.
5) **Post-processing:** Select outputs to visualize from the Options button/window, and define and analyse any region of interest if required (Section 6.2).
6) Use the navigation buttons above if necessary to move between screens.

10. Tutorial 1: Single Image Mode

The set of examples listed in https://web.fe.up.pt/~fgm/biscap3d/ (Tutorial Examples) is assumed to be downloaded and unpacked to any root location. In these tutorials, folders and files are referred to via relative paths to this root location (e.g. "…\examples\set1"). The full sequence of steps for the first tutorial is presented in sequence.

1) **(Start)** Open BISCAP.
2) **(Select mode)** In the **Home** screen, click the Single Image Mode – 2D button.
3) **(Image selection)** Click the Image selection button and browse to "…\examples\set1\img1.tiff". "img1.tiff" is then displayed on the initial **Pre-processing** screen.

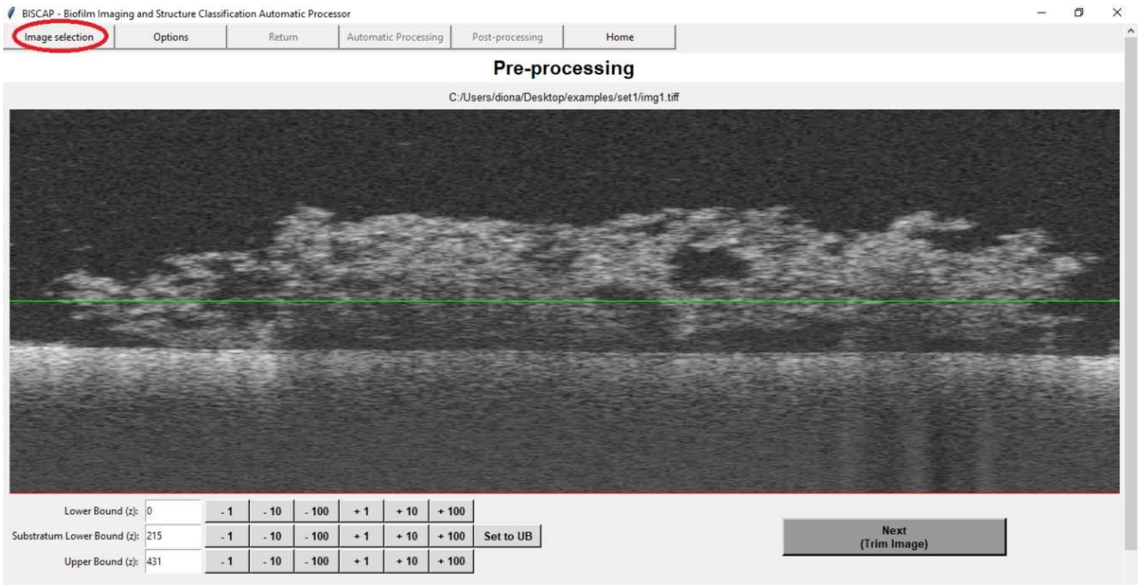

4) **(Pre-processing)** Using the increment buttons (-1, -10, -100, +1, +10, +100), set all bounds such that:
   - All biofilm pixels are below the *Lower Bound (z)*;
   - All substratum pixels are below the *Substratum Lower Bound (z)*;
   - All biofilm pixels are above the *Upper Bound (z)*.

   For reference, values used in the original contribution for this example are: *Lower Bound (z)* = 100, *Substratum Lower Bound (z)* = 262, and *Upper Bound (z)* = 281. When the specification is complete, click the Next (trim image) button.

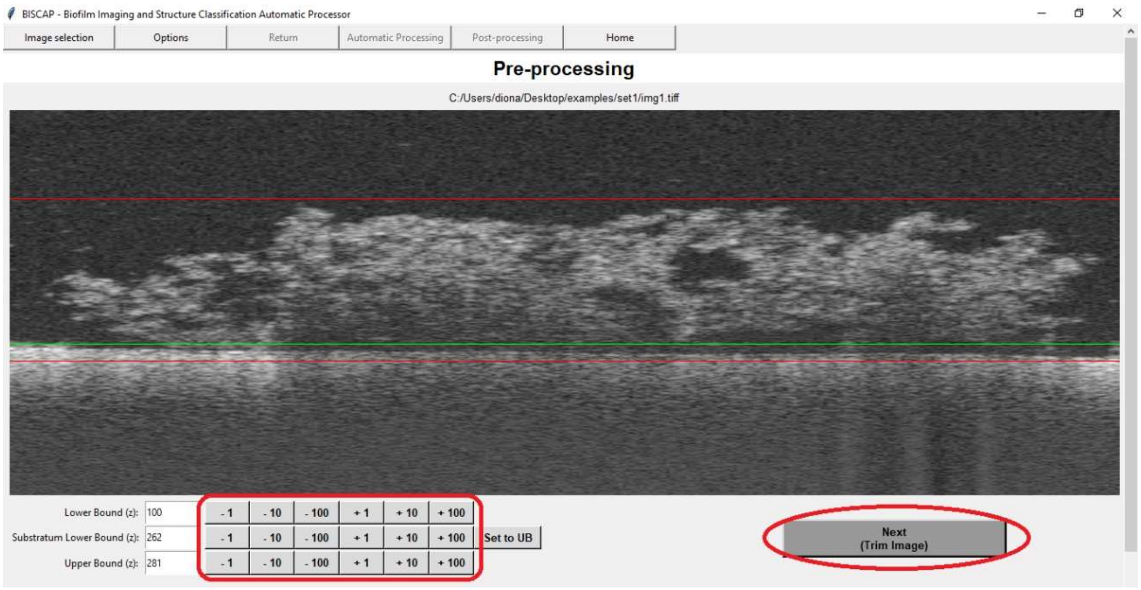

5) **(Automatic Processing)** The pre-processed image is displayed, and if a correction is required on the defined bounds, click the Return button to make the necessary adjustments (step 4). When complete, click the Next (Automatic Processing) button to begin automatic processing.

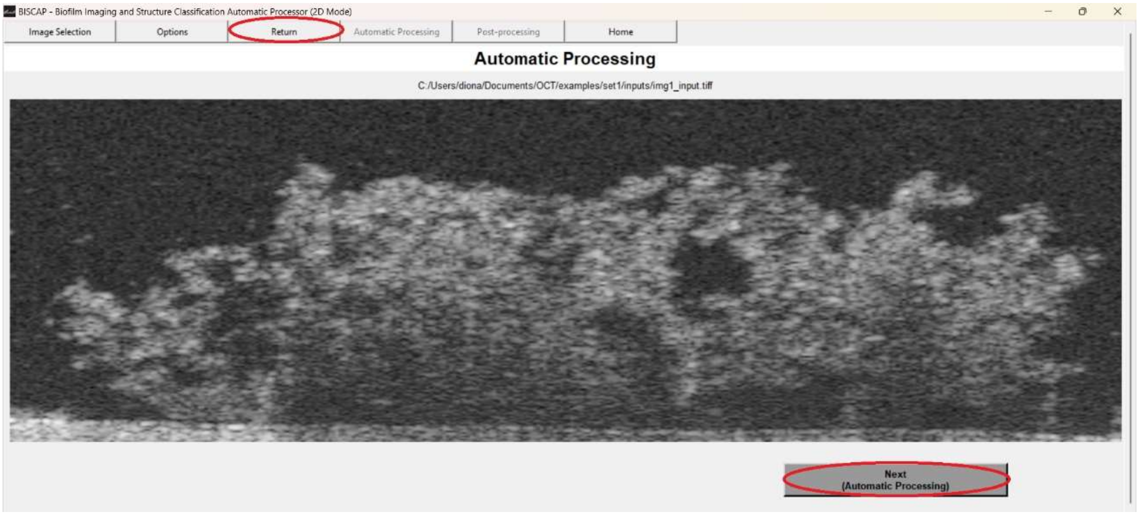

6) **(Results display)** All results from automatic processing are displayed in the **Post-processing** screen, including by default the biofilm region and structural properties (numeric results on the right side of the screen). From the Options button, select and visualise the remaining outputs.

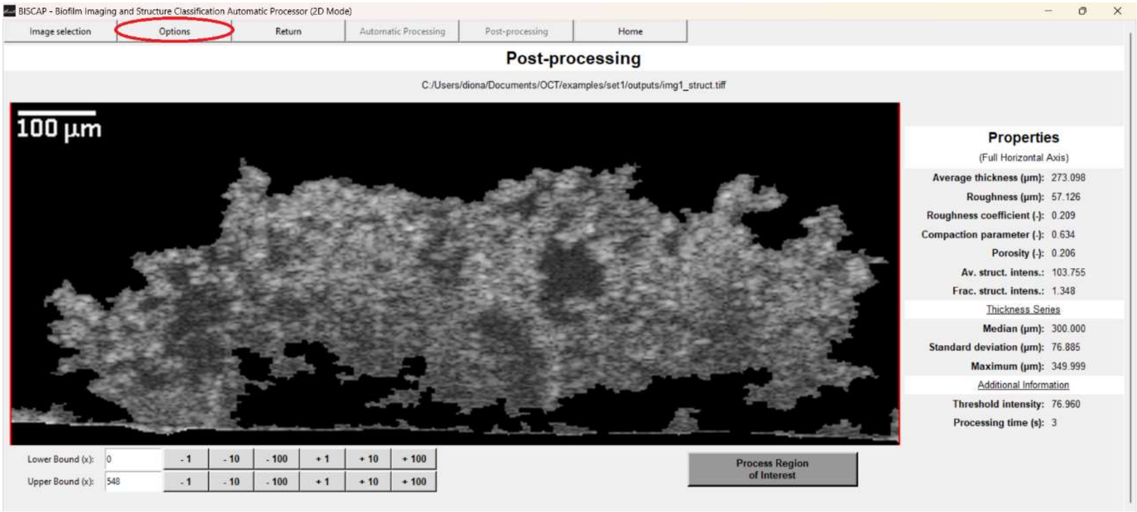

7) **(Post-processing)** Using the increment buttons (-1, -10, -100, +1, +10, +100), set the *Lower Bound (x)* and the *Upper Bound (x)* to define any region of interest. 20 and 148 are suggested, for the two parameters, respectively. Then, click the Process Region of Interest button to display and save the corresponding region of interest and structural parameters.

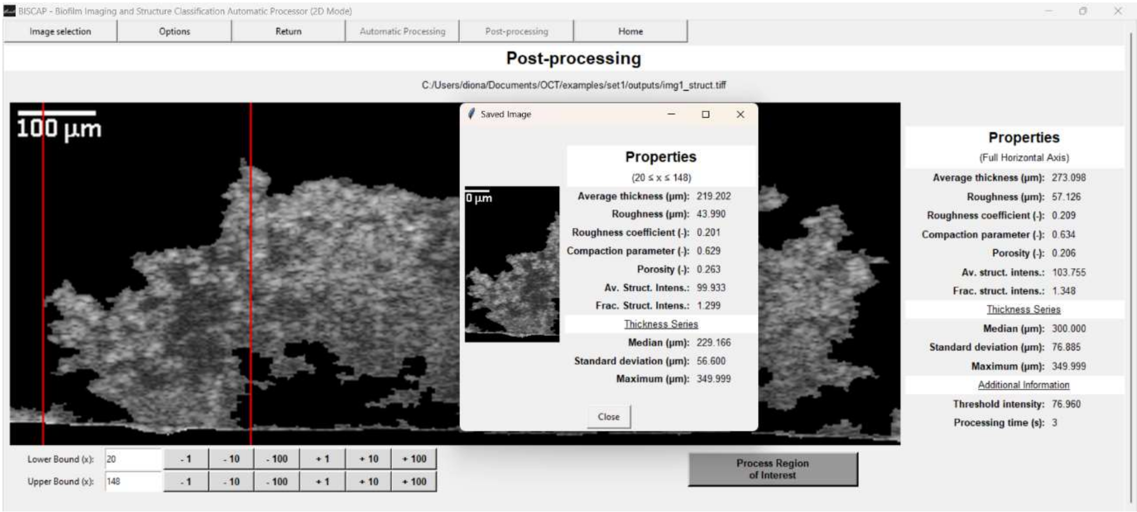

11. Tutorial 2: Multi-images Mode 2D

1) **(Start)** Open BISCAP.
2) **(Select mode)** In the **Home** screen, click the Multi-Images Mode – 2D button. The **Multi-images mode** screen is displayed, providing access to the three key image processing functionalities and corresponding screens: (i) **Pre-processing**, (ii) **Automatic Processing**, and (iii) **Post-processing**.

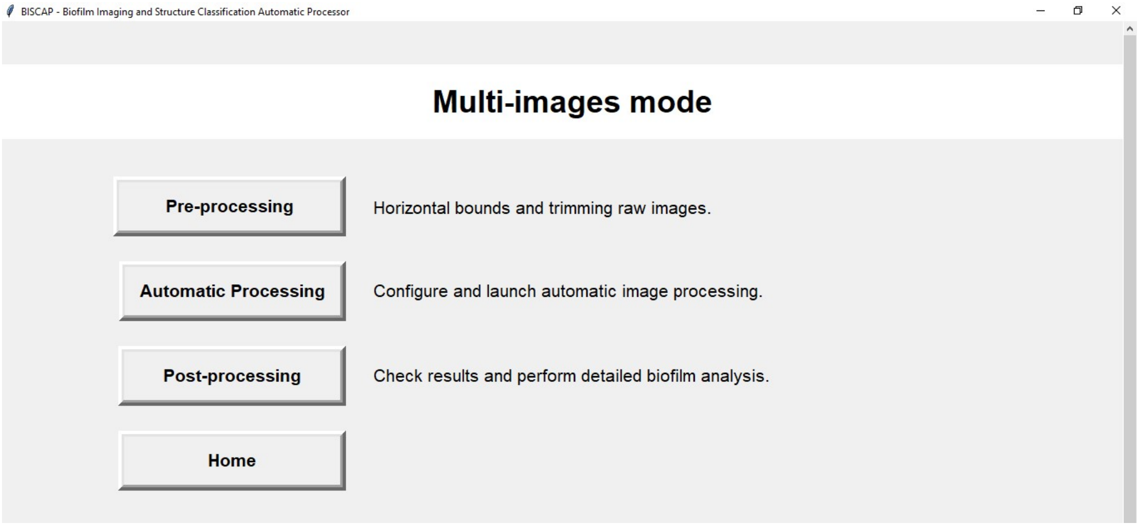

3) **(Pre-processing – part I)** Click the Pre-processing button. In the **Pre-processing** screen, click the Image selection button and browse to "…\examples\set2".

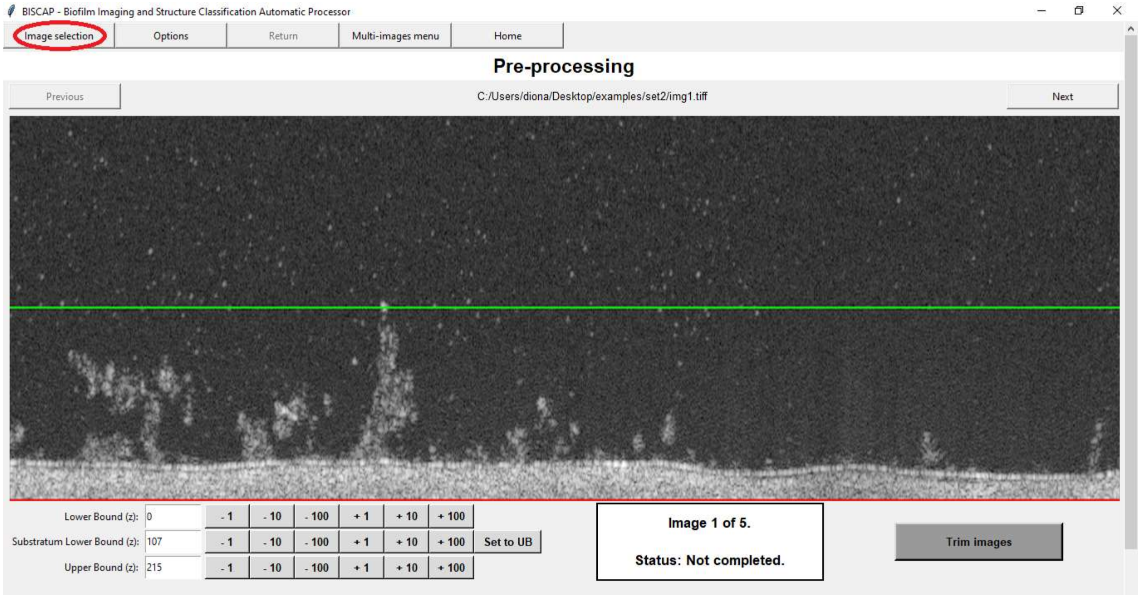

4) **(Pre-processing – part II)** Using the Next and Previous buttons and all increment buttons available on the bottom of the **Pre-processing** screen, set all bounds in accordance with the guidelines in Sections 3 or 8.

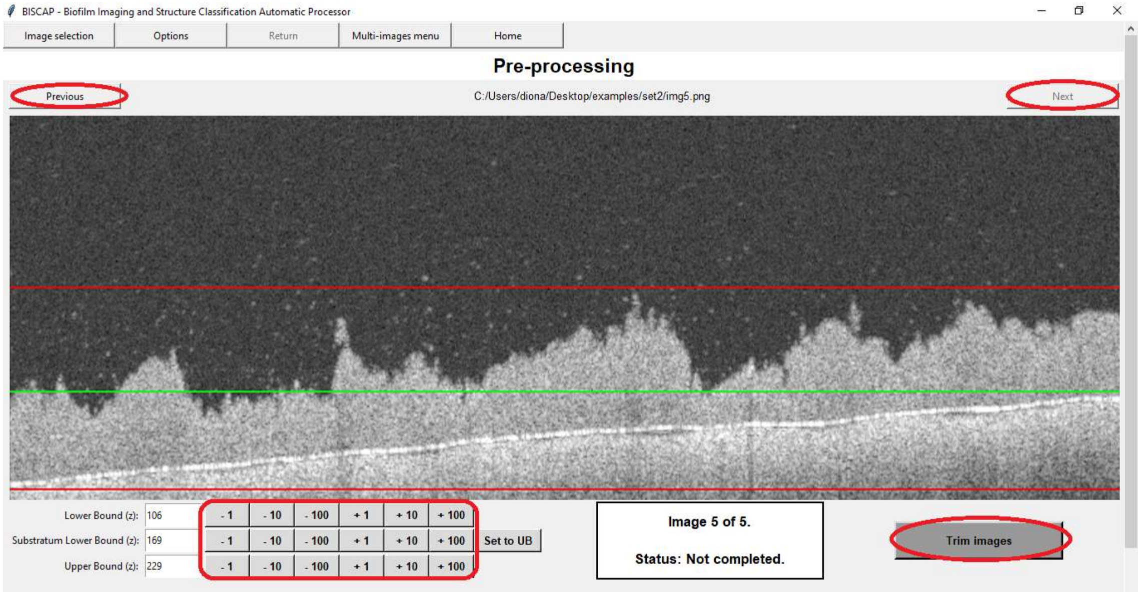

For reference, the bounds used for this set of images are presented in the Table below:

**Image**	**img1**	**img2**	**img3**	**img4**	**img5**
Lower Bound	103	105	104	106	106
Substratum Lower bound	189	362	254	141	169
Upper Bound	205	399	301	179	229

5) **(Pre-processing – part III)** Click the Trim Images button to automatically trim all raw images accordingly to the specifications in step 4. All pre-processed images may then be visualized using the Next and Previous buttons. For any additional adjustment, click the Return button. When completed, click the Multi-Images mode button to return to the main **Multi-images mode** screen.

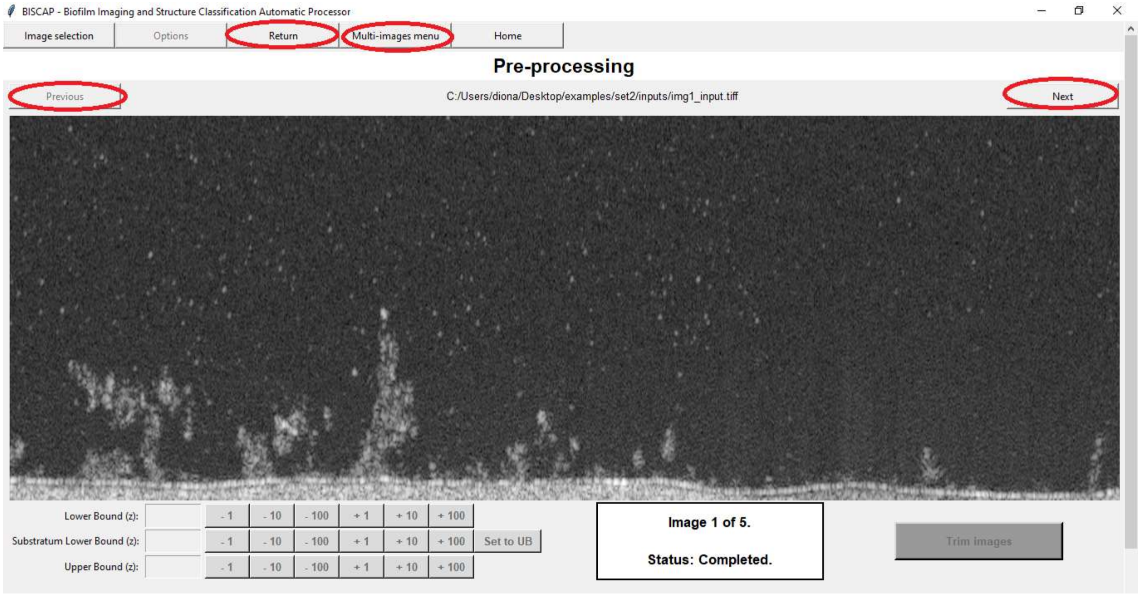

6) **(Automatic Processing – part I)** Click the Automatic Processing button. In the **Automatic Processing** screen, click the Image selection button and browse to folder "…\examples\set2". Click the Start Automatic Processing button to begin automatic processing. To process additional files (optional), click the Add Images button and select a folder of interest where pre-processing tasks were also completed in a prior stage (e.g. "…\examples\set3").

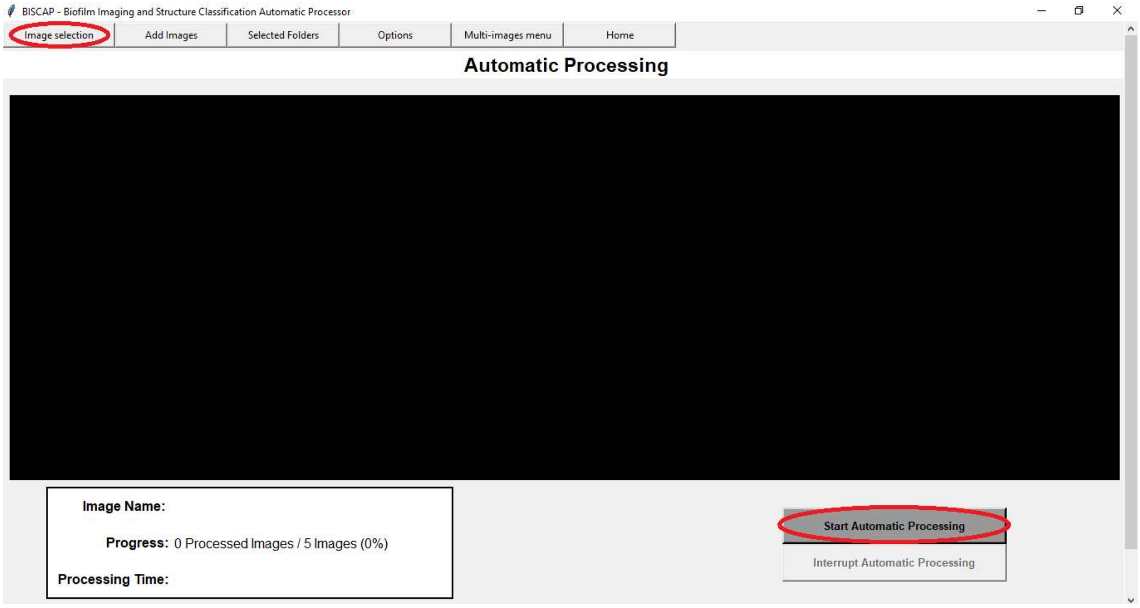

7) **(Automatic Processing – part II)** BISCAP processes in sequence all images selected. The current image under automatic processing is displayed, and the progress toward completion is shown below. On completion, click the Multi-Images mode button to return to the **Multi-Images mode** screen.

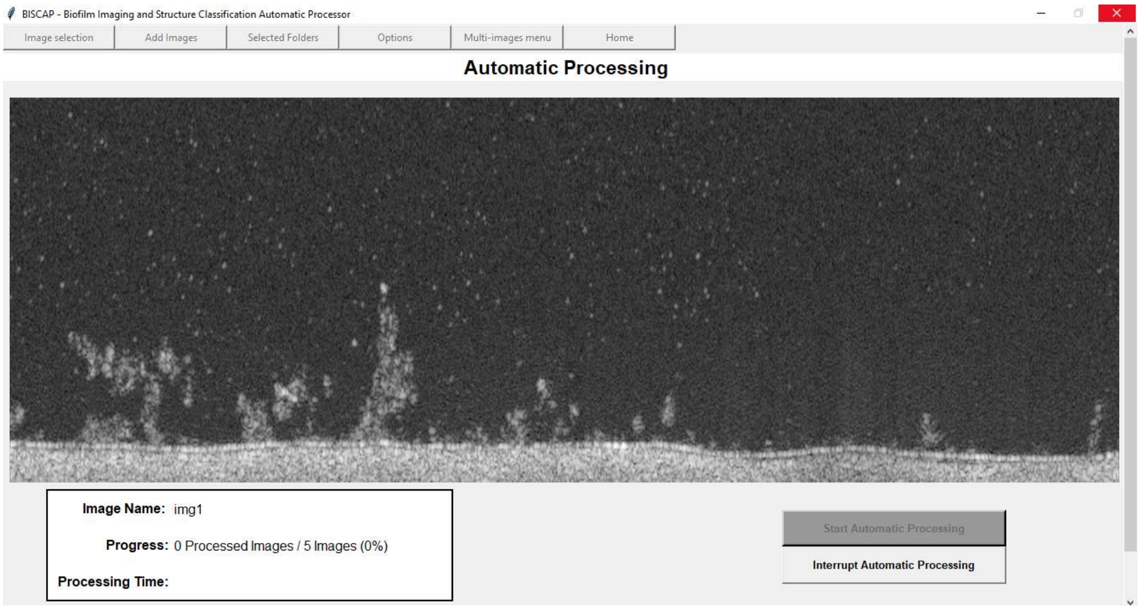

8) **(Results display)** Browse to the "…\examples\set2\outputs" folder and open the "outputs.xlsx" file to check and compare the image processing results for the five images in the selected root folder.

	Average thick	dian thickr	dev. thickr	ax. thickne	Roughness	ness coef	ction para	Porosity	truct. Inte	Struct. Int	Threshold	nation Co	cessing Ti
**img1**	27.89845	12.49998	36.22102	160.4164	25.88315	0.92776	0.69769	0.12596	109.6414	1.40116	78.25035	1	2.00467
**img2**	88.30888	8.33332	126.1476	337.4995	113.4208	1.28436	0.4992	0.28377	114.6371	1.22793	93.35781	1	3.10525
**img3**	91.24666	37.49994	108.3923	347.9161	89.84237	0.98461	0.52589	0.21974	114.9519	1.31197	87.61773	1	2.77524
**img4**	53.43121	52.08325	13.64394	97.91651	10.83795	0.20284	0.98445	0.01415	145.5892	1.52547	95.43892	1	1.98989
**img5**	106.4143	106.2498	23.85769	174.9997	19.34264	0.18177	0.99063	0.00594	167.4111	1.73666	96.39804	1	2.25815

9) **(Post-processing)** Click the Post-processing button. Then, click the Image selection button and browse to folder "…\examples\set2". Using the Previous and Next buttons select any image within the selected folder. Then, repeat steps 6 and 7, as presented in the first tutorial, to visualise all outputs and define any region of interest.

12. Tutorial 3: Single Image Mode 3D

1) **(Start)** Open BISCAP.
2) **(Select mode)** In the **Home** screen, click the <u>Single Image Mode – 3D</u> button.
3) **(Image selection)** Click the <u>Image selection</u> button and browse to "…\examples\set4\img1.tiff". "img1.tiff" is then displayed on the initial **Pre-processing** screen ("img2.tiff" was used for illustration purposes in the main manuscript; "img1.tiff" was obtained as a smaller piece from "img2.tiff").

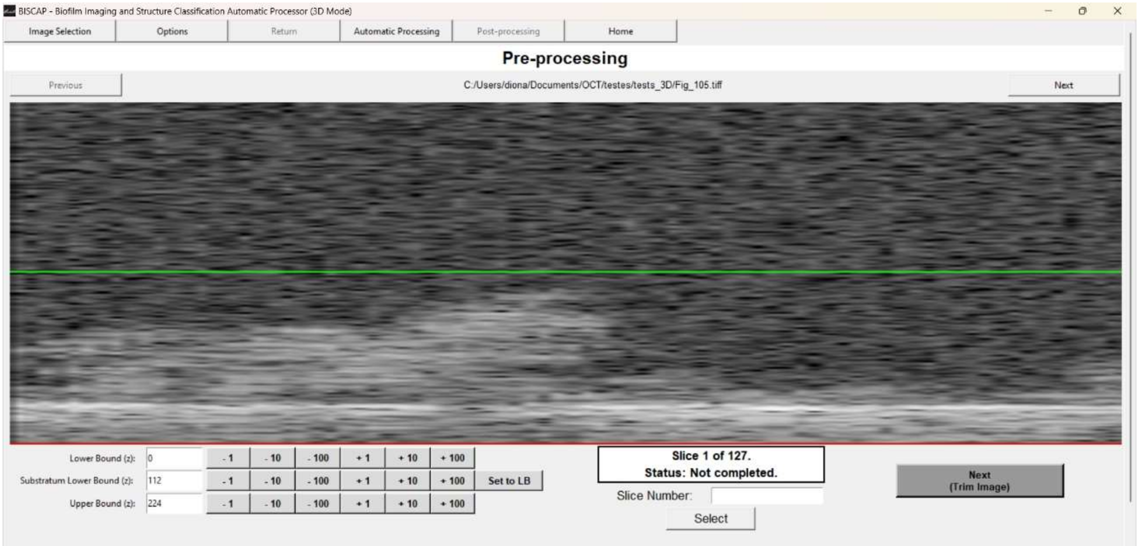

4) **(Pre-processing)** Using the increment buttons (<u>-1</u>, <u>-10</u>, <u>-100</u>, <u>+1</u>, <u>+10</u>, <u>+100</u>), set all bounds such that, for all constant y slices:
   - All biofilm pixels are <u>below</u> the *Lower Bound (z)*;
   - All substratum pixels are <u>below</u> the *Substratum Lower Bound (z)*;
   - All biofilm pixels are <u>above</u> the *Upper Bound (z)*.

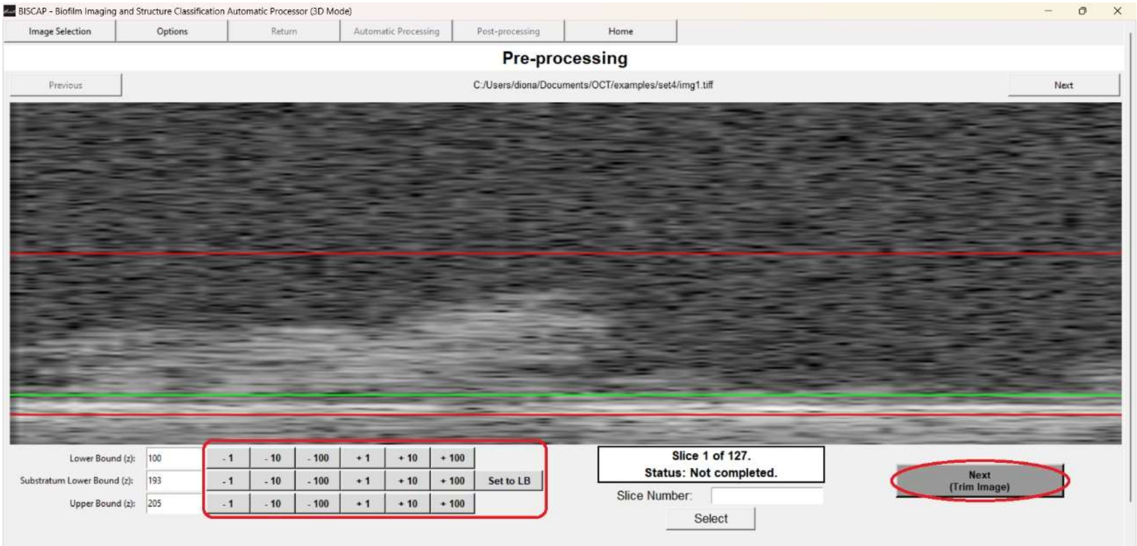

For reference, the bounds used for this example are: *Lower Bound (z)* = 100, *Substratum Lower Bound (z)* = 193, and *Upper Bound (z)* = 205. When the specification is complete, click the <u>Next (trim image)</u> button.

5) **(Automatic Processing)** The pre-processed image is displayed, and if a correction is required on the defined bounds, click the <u>Return</u> button to make the necessary adjustments (step 4). From the <u>Options</u> button/window, check that *Topography* is enabled and that parameter *y Bands* is set to 5. When complete, click the <u>Next (Automatic Processing)</u> button to begin automatic processing.

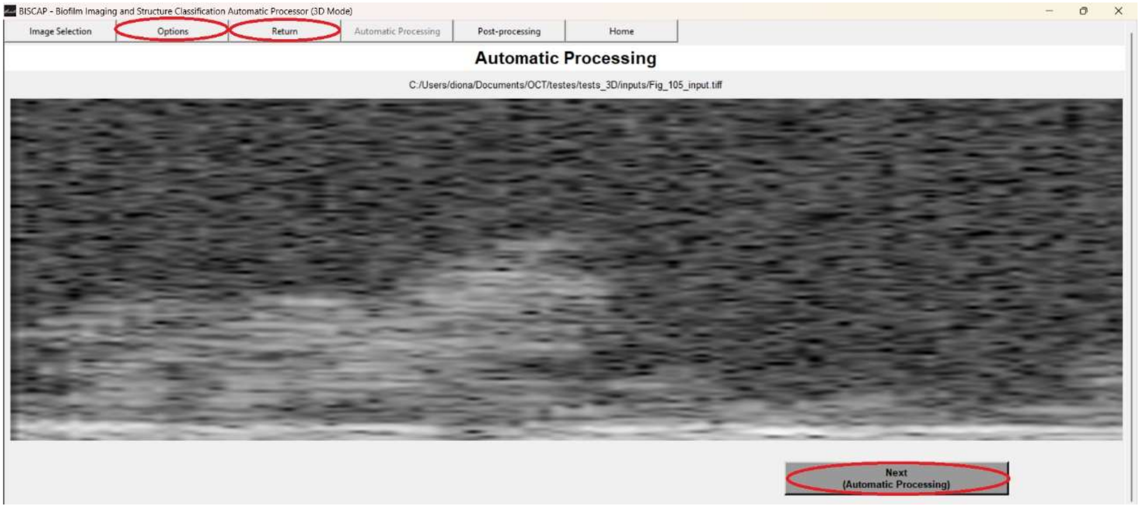

6) **(Results display)** All results from automatic processing are displayed in the **Post-processing** screen, including the thickness heatmap and all structural properties (right side of the screen).

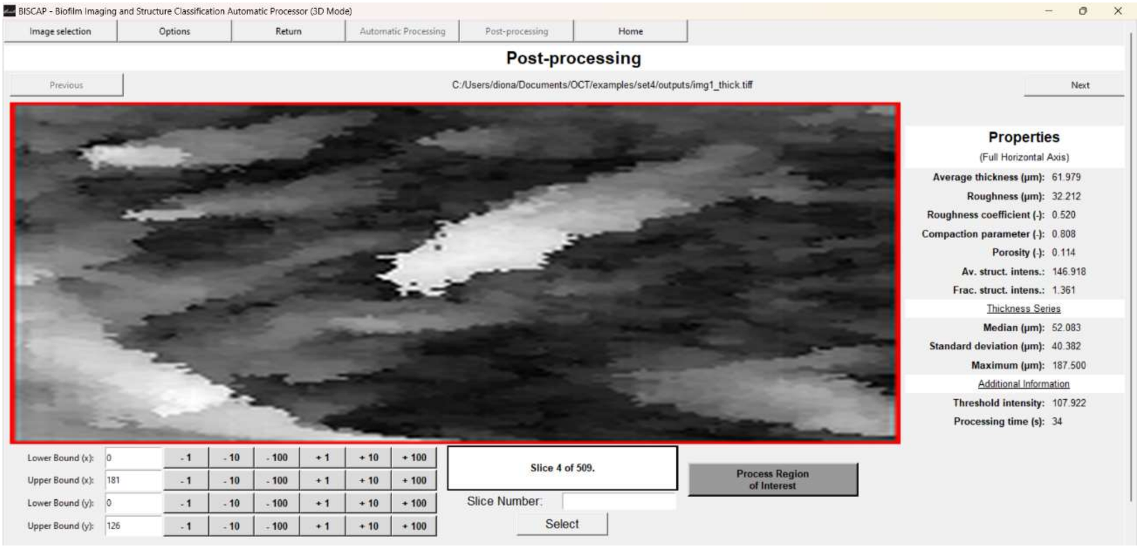

7) **(Post-processing: regions of interest)** Using the increment buttons (<u>-1</u>, <u>-10</u>, <u>-100</u>, <u>+1</u>, <u>+10</u>, <u>+100</u>), set the *Lower Bound (x)*, *the Upper Bound (x)*, the *Lower Bound (y),* and the *Upper Bound (y)* to define any region of interest. Values 70, 121, 50, and 96 are suggested, for these four parameters, respectively. Then, click the <u>Process Region of Interest</u> button to calculate the structural parameters in the defined region of interest.

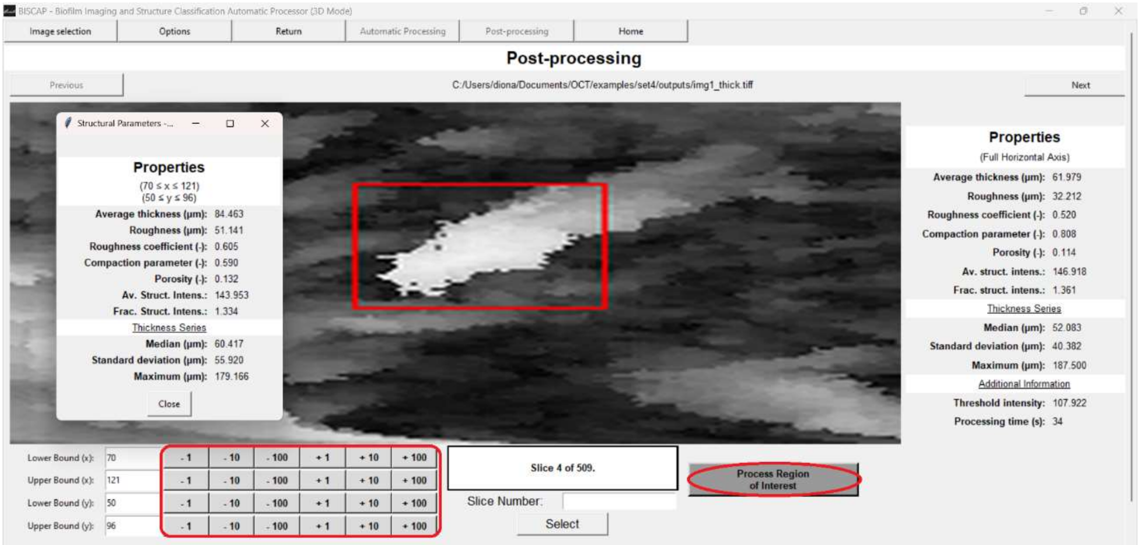

8) **(Post-processing: constant 2D slices)** From the Options button\window, select *Structure* and the vertical axis z. Then browse their slices using the Next button. At first, no biofilm voxels are observed, and as the view approaches the substratum, the biofilm density increases. A slice at $z = 115$ is displayed below.

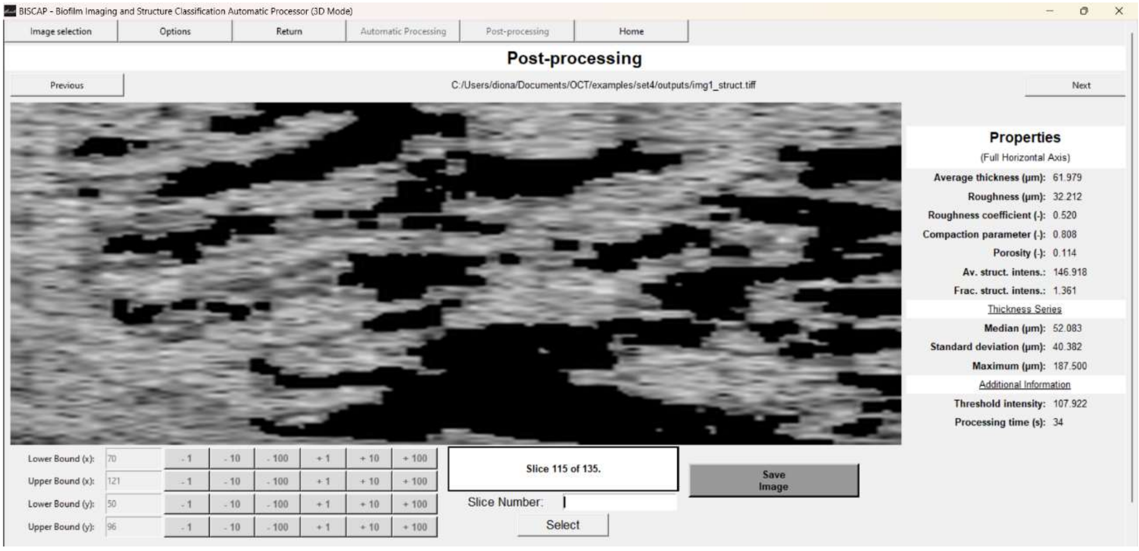

9) **(Post-processing: topography)** From the Options button, select *Topography*. The voxels at the top interface (water side) are displayed in a 3D representation.

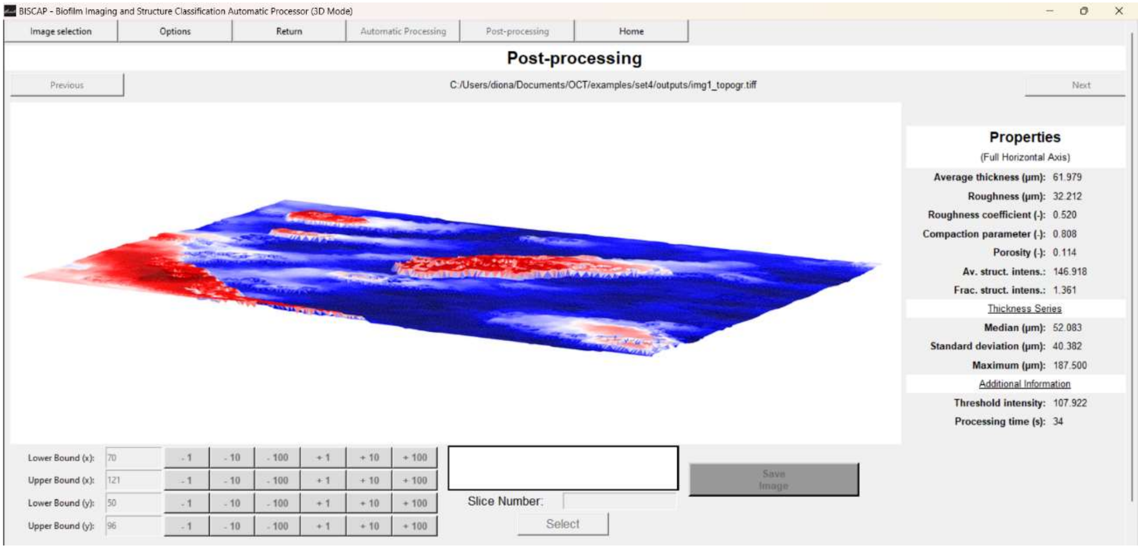
BISCAP - Biofilm Imaging and Structure Classification Automatic Processor (3D Mode)
Image selection
Options
Return
Automatic Processing
Post-processing
Home
Post-processing
Previous
C:/Users/diona/Documents/OCT/examples/set4/outputs/img1_topogr.tiff
Next
Properties
(Full Horizontal Axis)
Average thickness (µm): 61.979
Roughness (µm): 32.212
Roughness coefficient (-): 0.520
Compaction parameter (-): 0.808
Porosity (-): 0.114
Av. struct. intens.: 146.918
Frac. struct. intens.: 1.361
Thickness Series
Median (µm): 52.083
Standard deviation (µm): 40.382
Maximum (µm): 187.500
Additional Information
Threshold intensity: 107.922
Processing time (s): 34
Lower Bound (x): 70
Upper Bound (x): 121
Lower Bound (y): 50
Upper Bound (y): 96
- 1
- 10
- 100
+ 1
+ 10
+ 100
Save Image
Slice Number:
Select
